# On Sampling-Times-Independent Identification of Relaxation Time and Frequency Spectra Models of Viscoelastic Materials Using Stress Relaxation Experiment Data

**DOI:** 10.3390/ma18184403

**Published:** 2025-09-21

**Authors:** Anna Stankiewicz, Sławomir Juściński, Marzena Błażewicz-Woźniak

**Affiliations:** 1 Department of Technology Fundamentals, Faculty of Production Engineering, University of Life Sciences in Lublin, 20-612 Lublin, Poland; 2 Department of Power Engineering and Transportation, Faculty of Production Engineering, University of Life Sciences in Lublin, 20-612 Lublin, Poland; slawomir.juscinski@up.lublin.pl; 3 Institute of Horticultural Production, University of Life Sciences in Lublin, 20-612 Lublin, Poland; marzena.wozniak@up.lublin.pl

**Keywords:** viscoelasticity, relaxation spectrum, linear relaxation modulus, stress relaxation test, experiment randomization, Lipchitz models, noise robustness

## Abstract

Viscoelastic relaxation time and frequency spectra are useful for describing, analyzing, comparing, and improving the mechanical properties of materials. The spectra are typically obtained using the stress or oscillatory shear measurements. Over the last 80 years, dozens of mathematical models and algorithms were proposed to identify relaxation spectra models using different analytical and numerical tools. Some models and identification algorithms are intended for specific materials, while others are general and can be applied for an arbitrary rheological material. The identified relaxation spectrum model always depends on the identification method applied and on the specific measurements used in the identification process. The stress relaxation experiment data consist of the sampling times used in the experiment and the noise-corrupted relaxation modulus measurements. The aim of this paper is to build a model of the spectrum that asymptotically does not depend on the sampling times used in the experiment as the number of measurements tends to infinity. Broad model classes, determined by a finite series of various basis functions, are assumed for the relaxation spectra approximation. Both orthogonal series expansions based on the Legendre, Laguerre, and Chebyshev functions and non-orthogonal basis functions, like power exponential and modified Bessel functions of the second kind, are considered. It is proved that, even when the true spectrum description is entirely unfamiliar, the approximate sampling-times-independent spectra optimal models can be determined using modulus measurements for appropriately randomly selected sampling times. The recovered spectra models are strongly consistent estimates of the desirable models corresponding to the relaxation modulus models, being optimal for the deterministic integral weighted square error. A complete identification algorithm leading to the relaxation spectra models is presented that requires solving a sequence of weighted least-squares relaxation modulus approximation problems and a random selection of the sampling times. The problems of relaxation spectra identification are ill-posed; solution stability is ensured by applying Tikhonov regularization. Stochastic convergence analysis is conducted and the convergence with an exponential rate is demonstrated. Simulation studies are presented for the Kohlrausch–Williams–Watts spectrum with short relaxation times, the uni- and double-mode Gauss-like spectra with intermediate relaxation times, and the Baumgaertel–Schausberger–Winter spectrum with long relaxation times. Models using spectrum expansions on different basis series are applied. These studies have shown that sampling times randomization provides the sequence of the optimal spectra models that asymptotically converge to sampling-times-independent models. The noise robustness of the identified model was shown both by analytical analysis and numerical studies.

## 1. Introduction

Spectra of relaxation times and frequencies are the basic mechanical characteristics of rheological materials, especially polymers [1,2,3]. The spectra, providing a deep insight into the viscoelastic complex behavior, are fundamental for constitutive models used to describe, analyze, and shape the mechanical properties of materials [3,4,5,6]. However, the spectra are immeasurable and must be determined from the oscillatory shear or the stress relaxation experiment data. The problems of relaxation spectra identification are the numerical tasks of solving systems of first-kind Fredholm integral equations describing the data of the relaxation or dynamic moduli measurements [7], which are well-known ill-posed inverse problems [8]. Therefore, to solve the numerically difficult problem of recovering the relaxation spectra, special, stable algorithms are necessary, which are different for data collected in the time and frequency domains.

Since the late 1940s, when the first studies on the determination of relaxation spectra were carried out [9,10], many methods and algorithms of relaxation spectra identification have been proposed. Motivated by different identification techniques and experimental studies conducted for miscellaneous materials, based on diverse analytical and numerical tools, different classes of algorithms have been developed using both the relaxation modulus [11,12,13,14,15,16,17,18] and the storage and loss moduli [4,19,20,21,22,23,24,25,26,27,28,29] measurements. Reviews of the spectra models and identification algorithms from different perspectives can be found in many articles, for example, [24,30,31] and most recently in [18,28,32,33].

The first studies on the relaxation spectra recovery [9,10,34], from the late 1940s and early 1950s, are intended for relaxation modulus data and are inspired by the known Laplace transform rules. Alfrey and Doty [35,36] developed a simple differential model that resulted from the first-order Post–Widder formula [37] of the inverse Laplace transform. The approximated spectrum model developed by Ter Haar [34] is given by the product of the modulus and relaxation frequency inverse, which is essentially an implementation of the zero-order Post–Widder formula. The idea of the Post–Widder rule application has returned half a century later in the Bažant and Yunping [11] and Zi and Bažant [12] approach of fitting the relaxation modulus measurements via multiple-differentiable models and, next, by applying the Post–Widder rule to the determination of the spectrum model. For example, in [11], a logarithmic–exponential model is introduced, such that the authors accept a third-order Post–Widder approximation to be satisfactory to achieve the effectiveness of this approach. Recently, Cho et al. [33] applied the Post–Widder rule to the numerical calculation of the relaxation spectrum based on a double-logarithmic-series-type model of the modulus obtained by the optimal B-spline regression [38], which can be used for an arbitrarily high order of approximation. The use of the differential Post–Widder rule, which can be directly applied to the definition formula of the spectrum, will probably become an inspiration for further relaxation spectra identification algorithms that we can expect in the near future.

Also, the concept of identifying relaxation spectrum models using the familiar pairs of Laplace transforms, inaugurated in the mid-20th century by Macey [9], refers directly to the spectrum definition formula. Macey [9] described the modulus of a ceramic material by the modified zero-order second-kind Bessel function; a corresponding exponential–hyperbolic model of the spectrum was determined. When describing the mechanical properties of polyisobutylene, Sips [10] introduced a simple model of the relaxation spectrum given by the difference in two exponential functions, which means approximating the modulus with a logarithmic model. Then, Yamamoto [39], studying the viscoelasticity of the plant cell wall, extended this model by taking into account the long-term modulus.

The Post–Widder rule and Laplace transform pair algorithms assumed rather narrow classes of models; an exception is [33], where the series power-logarithmic model was used. Much richer model classes were applied in the next type of spectra recovery schemes—the algorithms of the regularized least-squares optimal approximation of the spectra in the class of models given by a series of various basis functions [16,17,40]. Both orthogonal [16] and not-orthogonal [17,40] basis functions were applied, and both relaxation frequency [16,40] and time [17,40] relaxation spectra were considered. Classic generalized cross-validation [8,41] was used for the regularization parameter selection [16,17], and a smoothing constraint method was applied [40] to ensure the identification scheme stability and model smoothness. By minimizing the spectrum’s integral square norm, in [18], the best smoothed relaxation time spectrum model was found, which reproduces the modulus measurements with an acceptably low error for the modulus approximation. This model was proved to be given by a series of exponential–hyperbolic basis functions [40].

Since the 1980s, in parallel, research has been developing on the recovery of relaxation spectra from oscillatory shear experiment data. Baumgaertel and Winter [19] applied a nonlinear least-squares identification for determining discrete spectra of relaxation and retardation times using oscillatory shear data, in which, during the successive iterations, the number of relaxation/retardation times were adjusted to increase the accuracy of the model without losing the well-posedness of the scheme. Honerkamp and Weese, using frequency–domain measurements, combined Tikhonov regularization with the linear least-squares method for discrete spectrum identification [20], and nonlinear least-squares identification for the calculation of the continuous spectrum [22]. Malkin [42] described a continuous relaxation spectrum with the following constants: slope in the logarithmic scale, the maximum relaxation time, and form factor. Next, Malkin et al. [43] proposed an algorithm for spectrum recovery using the Mellin integral transform. An approach by Stadler and Bailly [24] approximated the continuous spectrum by piece-wise cubic Hermite splines. Davies and Goulding [24] modeled the relaxation spectrum by a sum of appropriately located kernel functions. Next, two double-logarithmic power series models were adopted for the relaxation time spectrum identification by Cho [44] and Bae and Cho [45]. Cho [44] applied the power series of the logarithm of relaxation time to the spectrum modeling, while Bae and Cho [45] approximated the logarithm of the spectrum by a power series of the logarithm of relaxation time. In both algorithms [44,45], the Levenberg–Marquardt method was applied for the least-squares optimization, taking into account the ill-posedness of the spectrum recovery task. Anderssen et al. [4] and Davies et al. [46] considered derivative-based approximations of the continuous relaxation spectra using dynamic moduli experiment data; the approximations use exact inversion formula. High-order differentiations of oscillatory shear measurements were obtained using a stable iterative algorithm of the Gureyer type [4] and wavelet smoothing [46]. Determination of the relaxation spectra using the singular value decomposition combined with the Akaike information criterion estimator [47], application of the Bayesian inference [48,49], and the hierarchical Bayesian approach [50] are examples of a class of algorithms using mathematical, statistical, and stochastic tools. These and many other papers motivated and opened new directions of research on relaxation spectra identification using frequency–domain measurement data, which are being carried out by, e.g., [28,29,51,52].

In summary, many different models, methods, algorithms, and computational techniques have been developed over the last 80 years for relaxation spectra identification. Numerous analytical and numerical mathematical tools and system identification ideas have been applied to obtain the best relaxation spectrum model. However, the identified spectrum model is never a true description of the relaxation spectrum of the real material, since the model will always differ from the exact description of the modeled characteristic. Thus, the model usually only approximates the true relaxation spectrum. Model parameters are, in general, determined by the ‘best possible’ approximation of the measurements. Most of the known relaxation spectra identification methods use the mean sum of the errors between the relaxation or dynamic moduli models and the respective experiment data as a model quality criterion. The pure least-squares identification or regularized least squares were applied. The model determined obviously depends on the identification criterion, and, if the real relaxation spectrum does not belong to the pre-assumed model set, this dependence cannot be avoided. But the model parameters usually also depend on the experimental data.

This paper deals with the identification of the relaxation time and frequency spectra using noise-corrupted discrete measurements of the relaxation modulus recorded in a two-phase stress relaxation experiment, where time-dependent shear stress is measured for the unit step strain [1,2,3]. The data set is composed of the experiment’s sampling times and on relaxation modulus measurements. The goal of this paper is to build the optimal relaxation spectrum model, which is asymptotically independent of the specific sampling times used in the experiment, when the number of measurements tends to infinity.

Previously, in [53], the problem of weighted least-squares relaxation modulus approximation was formulated and solved under the assumption that only the noise-corrupted modulus measurements are available. It was shown in [53] that, for differentiable and Lipschitz continuous models, approximate optimal model parameters, being asymptotically independent on the sampling times, can be obtained by appropriate experiment randomization even in the case of a completely unknown description of the real modulus. In the current paper, this concept of the sampling times randomization is applied to relaxation spectra determination.

A broad class of models describing the spectra of relaxation frequencies and times by a series of various basis functions is considered. Orthogonal basis functions, like Legendre, Chebyshev, and Laguerre functions, and series expansions using non-orthogonal basis functions, e.g., power exponential and modified Bessel functions of the second kind, are considered. The technique of expanding relaxation spectra into a series of functions has been successfully used for identifying relaxation spectra in both time [16,17,32,40] and frequency [24,27,33,44,45] domains.

The main results concern the relaxation time and frequency models determined asymptotically with the number of measurements tending to infinity. It is demonstrated that approximate models of the spectra that are asymptotically independent on sampling times can be determined if the sampling times are chosen randomly according to an appropriate simple randomization of the experiment, and certain, not very restrictive, conditions are met regarding the basis functions and the experiment conditions. It is shown that the most commonly used basis functions satisfy these conditions. The approximate optimal spectra models are proved to be a strongly consistent estimate of the sampling- time-independent model. The stochastic convergence analysis is carried out for the relaxation modulus disturbed measurements and the exponential convergence is demonstrated. The results of the asymptotic properties and noise robustness numerical studies of the identified relaxation spectra models, carried out for weak and strong measurement disturbances, are presented. In the context of an ill-posed relaxation spectra identification problem, simulation studies based on the pre-assumed known real relaxation spectrum description allow us, apart from the analytical analysis, to demonstrate the algorithm effectiveness. In these studies, the identified spectrum is known, so the effectiveness of the identification algorithm can be assessed. The simulations were carried out for the Kohlrausch–Williams–Watts spectrum with short relaxation times, uni- and double-mode Gauss-like spectra with intermediate relaxation times, and the Baumgaertel–Schausberger–Winter spectrum with long relaxation times.

From the methodological point of view, our approach fits both into the class of Tikhonov-based methods and Laplace inversion-based methods used to recover relaxation spectra from measurements collected in the time domain. The approach, combining regularized least-squares identification with a random selection of sampling times, lies, in fact, in the widely understood Tikhonov regularization and Monte Carlo framework. Tikhonov-regularized least-squares identification using models described by a series of different basis functions has been previously applied to determine relaxation time and frequency spectra [16,17,32,40]. However, the concept of experiment randomization—that the identified models are asymptotically independent on sampling times—is used here to determine spectra models for the first time.

Both our approach and the newer inverse Laplace methods [11,12,33] use, in fact, a two-stage identification scheme, according to which, in the first stage, a relaxation modulus model that optimally approximates the measurement data is determined, and only in the second stage a relaxation spectrum model is derived. In our approach, similarly as in the previous papers [16,17,40], the identified relaxation spectrum model directly corresponds to the optimal model of the relaxation modulus, i.e., is given by the exact inverse Laplace transform of the modulus model. Therefore, in the second stage of the scheme, no approximation error is made when determining the relaxation spectrum model. In the inverse Laplace methods, in the second stage, the spectrum model is determined as a Post–Widder approximation of the assumed order of the inverse Laplace transform from the modulus model. Therefore, only an approximation of the spectrum corresponding to the optimal modulus model is determined, the error of which depends on the order adopted for the Post–Widder formula. Therefore, both stages of the inverse Laplace algorithms are subject to approximation errors, while, in our approach, the approximation occurs only in the first stage of the scheme.

Some of the tables and figures are moved to the Supplementary Materials. In Appendix A and the Supplementary Materials, the exemplary relaxation spectra and modulus models are presented using different basis functions, and their properties important for the identification concept are discussed. In Appendix B, the abbreviations and main symbols are summarized.

## 2. Materials and Methods

The assumptions of the rheological material and classes of models describing the relaxation frequency and time spectra are given and discussed.

A class of relaxation spectra models given by a finite series of basis functions is introduced. A quadratic identification index, defined in terms of the relaxation modulus measurements, is taken as a measure of the model accuracy. Then, the problem of determining the modulus model approximating the real, completely unknown modulus in a manner independent of particular sampling times is formulated by minimizing the weighted integral square error.

The concept of relaxation test randomization is presented, according to which randomly selected sampling times are sampled independently with identical probability distribution, with appropriate assumptions regarding the probability distribution and measurement noise.

### 2.1. Material

We consider a linear viscoelastic material subjected to small deformations for which the uniaxial and isotropic stress–strain relation is described by a Boltzmann integral equation [1,2,3]:

(1) $$\sigma \left(t\right)=\int_{-\infty }^{t}G\left(t-\tau \right)\dot{\varepsilon }\left(\tau \right)d\tau ,$$

with $\sigma \left(t\right)$ and $\varepsilon \left(t\right)$ denoting the stress and strain, and $G\left(t\right)$ being the linear relaxation modulus. The modulus $G\left(t\right)$ is the stress generated in material (1) after applying the unit step strain $\varepsilon \left(t\right)$. We assume that the modulus $G\left(t\right)$ exact description is entirely unknown. However, the real modulus $G\left(t\right)$ is measurable and can be recorded with some accuracy for any time t∈T. Here, $T=\left[t_{0},T\right]$ with T<∞, or $T=\left[t_{0},\right.\left.\infty \right)$, where the initial time $t_{0}\geq 0$. The factors determining the time interval T will be discussed further.

### 2.2. Spectra of Relaxation Frequencies and Times

It is assumed in rheology [1,4] that the modulus $G\left(t\right)$ has two equivalent integral representations given by

(2) $$G\left(t\right)=\int_{0}^{\infty }\frac{H\left(\tau \right)}{\tau }e^{-t/\tau }d\tau =\int_{0}^{\infty }\frac{H\left(v\right)}{v}e^{-tv}dv,$$

where relaxation frequency $H\left(v\right)$ and time $H\left(\tau \right)$ spectra, that characterize distributions of continuous relaxation frequencies v and times τ, are related as follows:

(3) $$H\left(\tau \right)=H\left(\frac{1}{\tau }\right),\;\;\;\;\;\;H\left(v\right)=H\left(\frac{1}{v}\right).$$

Following [16,32], the modified spectrum is defined as

(4) $$H^{M}\left(v\right)=\frac{H\left(v\right)}{v},$$

where, by the right equation in (2), we have

(5) $$G\left(t\right)=\int_{0}^{\infty }H^{M}\left(v\right)e^{-tv}dv,$$

i.e., the modulus $G\left(t\right)$ is the Laplace transform of $H^{M}\left(v\right)$. The upper index in $H^{M}\left(v\right)$ means ‘modified’.

### 2.3. Series Models

Let $H^{M}\left(v\right)\in L^{2}\left(0,\right.\left.\infty \right)$ and $H\left(\tau \right)\in L^{2}\left(0,\right.\left.\infty \right)$, where $L^{2}\left(0,\right.\left.\infty \right)$ is the space of real-valued square-integrable functions on $\left(0,\right.\left.\infty \right)$. From Theorems 3 and 4 in [54], the respective sufficient conditions can be easily derived.

Assume that the sets of the linearly independent functions $\left\{h_{1}\left(v\right),h_{2}\left(v\right),h_{3}\left(v\right),\ldots \right\}$ and $\left\{h_{1}\left(\tau \right),h_{2}\left(\tau \right),h_{3}\left(\tau \right),\ldots \right\}$ constitute bases of $L^{2}\left(0,\right.\left.\infty \right)$. Then, there exists such parameters $g_{k}$ that the spectrum of relaxation times can be written as

(6) $$H\left(\tau \right)=\sum_{k=1}^{\infty }g_{k}h_{k}\left(\tau \right).$$

For orthogonal functions $h_{k}\left(\tau \right)$, the so-called Fourier coefficients $g_{k}$ are given by [55]

(7) $$g_{k}=\int_{0}^{\infty }H\left(\tau \right)h_{k}\left(\tau \right)d\tau .$$

Analogously, for the relaxation frequency modified spectrum, we have

(8) $$H^{M}\left(v\right)=\sum_{k=1}^{\infty }g_{k}h_{k}\left(v\right),$$

with the formula for Fourier coefficients $g_{k}$ similar to (7) when basis functions $h_{k}\left(v\right)$ are orthogonal; examples of such basic functions will be considered below.

Replacing the infinite summation in (6) and (8) with a finite one of K first terms, we approximate the spectra $H\left(\tau \right)$ and $H^{M}\left(v\right)$ by finite series models as follows:

(9) $$H_{K}\left(\tau ,g_{K}\right)=\sum_{k=1}^{K}g_{k}h_{k}\left(\tau \right),$$

and

(10) $$H_{K}^{M}\left(v,g_{K}\right)=\sum_{k=1}^{K}g_{k}h_{k}\left(v\right),$$

where $g_{K}={\left[\begin{array}{ccc} g_{1} & \ldots & g_{K} \end{array}\right]}^{T}$ is vector of real parameters $g_{k}$ of models (9) and (10); the lower indices are the numbers of model summands.

When the relaxation spectra models (9) and (10) are applied, by Equations (2) and (5), the respective modulus models are also given by finite series as follows:

(11) $$G_{K}\left(t,g_{K}\right)=\int_{0}^{\infty }\frac{H_{K}\left(\tau ,g_{K}\right)}{\tau }e^{-t/\tau }d\tau =\sum_{k=1}^{K}g_{k}\phi_{k}\left(t\right),$$

and

(12) $$G_{K}\left(t,g_{K}\right)=\int_{0}^{\infty }H_{K}^{M}\left(v,g_{K}\right)e^{-tv}dv=\sum_{k=1}^{K}g_{k}\phi_{k}\left(t\right),$$

with the basis functions $\phi_{k}\left(t\right)$ of model (9) defined by

(13) $$\phi_{k}\left(t\right)=\int_{0}^{\infty }\frac{h_{k}\left(\tau \right)}{\tau }e^{-t/\tau }d\tau ,$$

while those defined by model (10) are

(14) $$\phi_{k}\left(t\right)=\int_{0}^{\infty }h_{k}\left(v\right)e^{-tv}dv.$$

Series models of the relaxation time spectrum have been, for example, considered in [17], where the spectrum was modeled by a series of power-exponential functions, that yielded basis functions $\phi_{k}\left(t\right)$ (13) given by products of the modified Bessel functions of the second kind and power of time. In turn, in [40], power-square-exponential basis functions were used for describing the frequency spectrum, which resulted in recurrence formulae with products of power of time and complementary error functions describing the relaxation modulus. Orthogonal expansions of the modified relaxation frequency spectrum have been considered in [16], where the Legendre, Hermite, Laguerre, and Chebyshev functions were considered in detail. Recently, a series of exponential basis functions was used to approximate the modified spectrum $H^{M}\left(v\right)$ [32].

Based on [16,17,32,40], the exemplary formulae for functions $h_{k}\left(\tau \right)$, $h_{k}\left(v\right)$, and $\phi_{k}\left(t\right)$ are presented in Appendix A.1, Appendix A.2, Appendix A.3 and Appendix A.4, which are used in the numerical studies; in Supplementary Materials S.1 and S.2, two additional examples are presented. The properties, in particular the monotonicity and definiteness of these functions, has been discussed in detail in the source papers. The units of the model parameters $g_{k}$ depend on the units of the basis functions, the units of which are specified in Appendix A.1, Appendix A.2, Appendix A.3 and Appendix A.4 and Supplementary Materials.

It is convenient to express the modulus models (11) and (12) in the following compact form:

(15) $$G_{K}\left(t,g_{K}\right)=g_{K}^{T}\phi_{K}\left(t\right),$$

where, analogously, for the relaxation modulus models (9) and (10), we have

(16) $$H_{K}\left(\tau ,g_{K}\right)=g_{K}^{T}k_{K}\left(\tau \right),$$

and

(17) $$H_{K}^{M}\left(v,g_{K}\right)=g_{K}^{T}h_{K}\left(v\right),$$

where the vector functions $\phi_{K}\left(t\right)$, $k_{K}\left(\tau \right)$, and $h_{K}\left(v\right)$ are composed of the respective model basis functions, as follows:

(18) $$\phi_{K}\left(t\right)=\left[\begin{array}{c} \phi_{1}\left(t\right)\\ \vdots \\ \phi_{K}\left(t\right) \end{array}\right],\;k_{K}\left(\tau \right)=\left[\begin{array}{c} h_{1}\left(\tau \right)\\ \vdots \\ h_{K}\left(\tau \right) \end{array}\right],\;h_{K}\left(v\right)=\left[\begin{array}{c} h_{1}\left(v\right)\\ \vdots \\ h_{K}\left(v\right) \end{array}\right]$$

Models (15)–(17) are linear functions of $g_{K}$. We will assume the real space $R^{K}$ as the set of admissible model parameters; however, we will show that, for the purposes of the convergence analysis, in the optimal identification tasks considered below, the condition $g_{K}\in R^{K}$ can be restricted to some compact subset $G\subset R^{K}$ (c.f., Proposition 1 below).

### 2.4. Least-Squares Regularized Identification Task

Suppose a stress relaxation experiment [1,2,3], performed for the sample of tested material, resulted in a set of measurements $\left\{\bar{G}\left(t_{i}\right)=G\left(t_{i}\right)+z\left(t_{i}\right)\right\}$ at the sampling times $t_{i}$, i=1,…,N, with $z\left(t_{i}\right)$ denoting additive measurement noise. By assumption, N≥K.

Identification consists of the selection, within the assumed model classes defined by (9) and (11) or (10) and (12), of such a model that establishes the best fit to the measurement data. The accuracy of models (11) or (12) is measured by the quadratic index, as follows:

(19) $$Q_{N}\left(g_{K}\right)=\frac{1}{N}\sum_{i=1}^{N}{\left[\bar{G}\left(t_{i}\right)-G_{K}\left(t_{i},g_{K}\right)\right]}^{2},$$

which is the basis for the least-squares identification of models (11) and (12).

The identification index (19) can be rewritten as

(20) $$Q_{N}\left(g_{K}\right)=\frac{1}{N}{\left\|{\bar{G}}_{N}-\Phi_{N,K}g_{K}\right\|}_{2}^{2},$$

where

(21) $$\Phi_{N,K}=\left[\begin{array}{ccc} \phi_{1}\left(t_{1}\right) & \ldots & \phi_{K}\left(t_{1}\right)\\ \vdots & \ddots & \vdots \\ \phi_{1}\left(t_{N}\right) & \ldots & \phi_{K}\left(t_{N}\right) \end{array}\right],\;\;\;\;\;\;{\bar{G}}_{N}=\left[\begin{array}{c} \bar{G}\left(t_{1}\right)\\ \vdots \\ \bar{G}\left(t_{N}\right) \end{array}\right]$$

and ${\left\|\cdot \right\|}_{2}$ is the Euclidean norm in $R^{N}$. The optimal relaxation spectrum identification relies on determining the model parameter $g_{K}$ that solves the optimization task:

(22) $$\underset{g_{K}\in R^{K}}{\min}\frac{1}{N}{\left\|{\bar{G}}_{N}-\Phi_{N,K}\;g_{K}\right\|}_{2}^{2}.$$

This problem is, generally, ill-posed [8,56] because matrix $\Phi_{N,K}$ is ill-conditioned. Whence, even small fluctuations in relaxation modulus measurements ${\bar{G}}_{N}$ can result in dramatically large artifacts in the $g_{K}$ solving task (22). Therefore, Tikhonov regularization [56] is applied, according to which the source problem (22) is replaced by

(23) $$\underset{g_{K}\in R^{K}}{\min}\left[\frac{1}{N}{\left\|{\bar{G}}_{N}-\Phi_{N,K}\;g_{K}\right\|}_{2}^{2}+\lambda {\left\|g_{K}\right\|}_{2}^{2}\right]=\underset{g_{K}\in R^{K}}{\min}\left[Q_{N}\left(g_{K}\right)+\lambda {\left\|g_{K}\right\|}_{2}^{2}\right],$$

with a regularization parameter λ>0 equilibrating both terms of (23). The regularized task (23) is well-posed; its unique solution is as follows:

(24) $${\bar{g}}_{N,K}^{\lambda }={\left(\Phi_{N,K}^{T}\Phi_{N,K}+\lambda NI_{K,K}\right)}^{-1}\Phi_{N,K}^{T}{\bar{G}}_{N}.$$

Regularized parameter ${\bar{g}}_{N,K}^{\lambda }$ (24) depends continuously on both $\Phi_{N,K}$ and the measurements vector ${\bar{G}}_{N}$. Here $I_{K,K}$ denotes the K-dimensional identity matrix.

Matrix $\Phi_{N,K}$ depends on the sampling times $\left\{t_{i}\right\}$ applied in the experiment; therefore, vector ${\bar{g}}_{N,K}^{\lambda }$ is affected both by $\left\{t_{i}\right\}$ and by the modulus measurements collected in ${\bar{G}}_{N}$. Therefore, for the best parameter ${\bar{g}}_{N,K}^{\lambda }$ (24), both $\left\{t_{i}\right\}$ and ${\bar{G}}_{N}$ determine the best spectra and modulus models (15)–(17). It was proved in [53] that, even when the real relaxation modulus description is unknown, the optimal model parameters can be found using the measurements ${\bar{G}}_{N}$ collected for appropriate sampling times $\left\{t_{i}\right\}$ whenever the class of models satisfy some applicability conditions. Below, we formulate these assumptions in terms of the models (11) and (12) to guarantee the applicability of this experiment randomization concept to the relaxation spectra identification task (23).

### 2.5. Assumptions

Following [53], we take the following assumptions:

**Assumption 1.**  *The relaxation modulus $G\left(t\right)$ of the material is bounded on T, $\underset{t\in T}{\sup}G\left(t\right)\leq M_{1}<\infty$*.

**Assumption 2.**  *The noises $z\left(t_{i}\right)$ are bounded, $\left|z\left(t_{i}\right)\right|\leq \delta <\infty$, i=1,…,N*.

**Assumption 3.**  *The basis functions $\phi_{k}\left(t\right)\;of\;the\;models\;G_{K}\left(t,g_{K}\right)$ (11) and (12) (jointly, (15)) are such that $\underset{t\in T}{\sup}{\left\|\phi_{K}\left(t\right)\right\|}_{2}{\leq M}_{2}<\infty$, where $\phi_{K}\left(t\right)$ is defined in (18)*.

**Assumption 4.**  *The basis functions $h_{k}\left(\tau \right)$ and $h_{k}\left(v\right)$ of the relaxation spectra models $H_{K}\left(\tau ,g_{K}\right)$ (16) and $H_{K}^{M}\left(v,g_{K}\right)$ (17) are such that $\underset{\tau \geq \tau_{0}}{\sup}{\left\|k_{K}\left(\tau \right)\right\|}_{2}{\leq M}_{\tau }<\infty$ and $\underset{v\geq v_{0}}{\sup}{\left\|h_{K}\left(v\right)\right\|}_{2}{\leq M}_{v}<\infty$, where $\tau_{0}\geq 0$, $v_{0}\geq 0$ and $k_{K}\left(\tau \right)$, and $h_{K}\left(v\right)$ are defined in (18)*.

Assumptions 1 and 2 are identical with Assumptions 1 and 6 in [53]. The boundness of the relaxation modulus and measurement noises is natural. For any t∈T the linear, differentiable with respect to $g_{K}$ model $G_{K}\left(t,g_{K}\right)$ (15) is such that $G_{K}\left(t,0_{K}\right)$, with $0_{K}$ being a K-dimensional zero vector in $R^{K}$; thus, Assumption 3 from [53] is fulfilled. In turn, Assumption 4 from [53] for $G_{K}\left(t,g_{K}\right)$ (15), being the linear function of $g_{K}$, is the current Assumption 3, and means that the gradient of $G_{K}\left(t,g_{K}\right)$ with respect to $g_{K}$ is uniformly bounded for t∈T. Thus, $G_{K}\left(t,g_{K}\right)$ is a Lipschitz function of $g_{K}$ for any t∈T. In turn, if Assumption 4 holds for the basis functions of the spectra models, then these models are Lipschitz functions of $g_{K}$ for any positive relaxation time and frequency. Assumptions 3 and 4 do not concern the material, but rather its models, which can be chosen freely to a certain extent. In Appendix A.1, Appendix A.2, Appendix A.3 and Appendix A.4 and Supplementary Materials S.1 and S.2, it is demonstrated that these assumptions are fulfilled for the exemplary models of relaxation spectra given by series expansions (see Properties A.1–A.4 and Supplementary Materials S.1 and S.2). The verification of Assumptions 3 and 4 should be performed individually for each basis function. As in Appendix A.1, Appendix A.2, Appendix A.3 and Appendix A.4 and Supplementary Materials S.1 and S.2, various known algebraic inequalities and known properties of certain special functions can be used. In summary, the above assumptions should not be a restriction.

We now return to the factors determining the set T. A quick review of these properties shows that, for the basis functions $\phi_{k}\left(t\right)$ (A11), (A15), (A22), (S9)–(S12), and (S26)–(S28) of the relaxation frequency spectrum model using exponential, Legendre, Laguerre, Chebyshev, and complementary error functions, respectively, the lower limit $t_{0}$ of the time interval T can be zero, while for the functions $\phi_{k}\left(t\right)$ (A3) using Bessel functions, a non-zero initial time $t_{0}$ must be assumed. If the basis functions $\phi_{k}\left(t\right)$ tend to a bounded constant—usually, to zero—as t→∞, then, for Assumption 3 to be satisfied, no condition needs to be imposed on the upper bound of the time interval T. All the basis functions described in Appendix A.1, Appendix A.2, Appendix A.3 and Appendix A.4 and Supplementary Materials S.1 and S.2 are asymptotically bounded.

To summarize, the set T, bounded $T=\left[t_{0},T\right]$ with T<∞ or unbounded $T=\left[t_{0},\right.\left.\infty \right)$, with the initial time $t_{0}>0$ or $t_{0}=0$, depends on the basis functions $\phi_{k}\left(t\right)$ of the relaxation modulus model (i.e., on the basis functions of the relaxation spectra models) and on the conditions of the relaxation experiment determining the times for which the relaxation modulus is available for measurement. In particular, the non-zero initial time may result from experimental conditions and/or from properties of the class of models that force $t_{0}>0$ in order to guarantee the required asymptotic properties of the spectra and modulus models. In turn, the finite or non-finite upper bound of the interval T is related, in addition to the conditions of a specific rheological experiment, to the choice of the model quality index. This issue will be discussed below.

### 2.6. Constrained Least-Squares Regularized Identification Task

In the source paper [53], the identification task was considered under the assumption that the set of admissible model parameters is compact. To exploit directly the convergence results of [53], in Appendix A.5, the following proposition was proved, which states the equivalence between the un-constrained regularized task (23) and a respective constrained optimization task.

**Proposition 1.**  *Let* λ>0 *. If Assumptions 1–3 are satisfied, then there exists a compact subset of* $R^{K}$ *:*

(25) $$G=\left\{g_{K}\in R^{K}:{\left\|g_{K}\right\|}_{2}\leq M_{3}<\infty \right\},$$

*with*

(26) $$M_{3}=\frac{M_{2}\left(M_{1}+\delta \right)}{\lambda }>0,$$

*such that*

$$\underset{g_{K}\in R^{K}}{min}Q_{N}\left(g_{K}\right)+\lambda {\left\|g_{K}\right\|}_{2}^{2}=\underset{g_{K}\in G}{min}Q_{N}\left(g_{K}\right)+\lambda {\left\|g_{K}\right\|}_{2}^{2}.$$

Similar result can be derived from Lemma 1 in [53], the proof of which was based on the Schwarz for the vectors’ inner product; however, resulting from this lemma, the upper bound ${\left\|g_{K}\right\|}_{2}\leq 2M_{3}$ in the definition (25) is weaker.

In view of the above, the next property is obtained, which consists in reducing the set of model admissible parameters to a compact set, which will allow direct adoption of the convergence results proved in [53] to the considered relaxation spectra identification task.

**Property 1.**  *The set of admissible model parameters can be reduced to G (25), being the compact subset of $R^{K}$*.

For any $g_{K}\in G$**,** the estimation holds ${\left\|g_{K}\right\|}_{2}\leq M_{3}<\infty$. Since, by the Schwarz inequality for the vectors’ inner product

$$\left|G_{K}\left(t,g_{K}\right)\right|=\left|g_{K}^{T}\phi_{K}\left(t\right)\right|\leq {\left\|g_{K}\right\|}_{2}{\left\|\phi_{K}\left(t\right)\right\|}_{2},$$

Assumption 4 directly implies

(27) $$\underset{t\in T,g\in G}{\sup}\left|G_{K}\left(t,g_{K}\right)\right|\leq M_{2}M_{3}=M_{4}<\infty ,$$

which means the uniform on T×G saturation imposed on the relaxation modulus model, and together with Assumption 1, yields the estimate

(28) $$\underset{t\in T,g\in G}{\sup}\left|G\left(t\right)-G_{K}\left(t,g_{K}\right)\right|\leq M_{1}+M_{4}<\infty .$$

### 2.7. The Optimal Relaxation Modulus Approximation

We consider the task of determining the modulus model $G_{K}\left(t,g_{K}\right)$ (15) that minimizes the integral approximation error [53]:

(29) $$Q\left(g_{K}\right)=\int_{T}{\left[G\left(t\right)-G_{K}\left(t,g_{K}\right)\right]}^{2}\rho \left(t\right)dt,$$

where the selected weight function, such that $0\leq \rho \left(t\right)\leq M_{0}<\infty$, is a density on the set T, i.e., $\int_{T}\rho \left(t\right)dt=1$. In view of (28), the function ${\left[G\left(t\right)-G_{K}\left(t,g_{K}\right)\right]}^{2}$ is bounded uniformly for $\left(t,g_{K}\right)\in T\times G$. Therefore, both for the bounded or unbounded domain T, the above integral is absolutely integrable, uniformly on G, as the product of a function bounded uniformly on T×G and the absolutely integrable function $\rho \left(t\right)$.

The problem of the real modulus $G\left(t\right)$ optimal approximation in the class of models defined by $G_{K}\left(t,g_{K}\right)$ (15) and the set G (25) consists in finding that parameter which minimizes $Q\left(g_{K}\right)$ on G. By (29) and (15), we have

$$Q\left(g_{K}\right)=\int_{T}{\left[G\left(t\right)\right]}^{2}\rho \left(t\right)dt+g_{K}^{T}{\bar{\Phi }}_{K}\;g_{K}-2{\bar{\phi }}_{K}^{T}g_{K},$$

where the K×K matrix ${\bar{\Phi }}_{K}$ and K-dimensional vector ${\bar{\phi }}_{K}$ are defined by the integrals

$${\bar{\Phi }}_{K}=\int_{T}\phi_{K}\left(t\right)\phi_{K}^{T}\left(t\right)\rho \left(t\right)dt,\;\;\;\;\;\;{\bar{\phi }}_{K}=\int_{T}G\left(t\right)\phi_{K}\left(t\right)\rho \left(t\right)dt.$$

with $\phi_{K}\left(t\right)$ defied by Equation (18).

If ${\bar{\Phi }}_{K}$ is ill-conditioned, the minimization of $Q\left(g_{K}\right)$ is Hadamard ill-posed. Then, a stable approximate solution can be determined by the minimization of the index composed by the sum of $Q\left(g_{K}\right)$ and a penalty term $\lambda {\left\|g_{K}\right\|}_{2}^{2}$, which results in the following regularized optimal relaxation modulus approximation task:

(30) $$\underset{g_{K}\in G}{min}Q\left(g_{K}\right)+\lambda {\left\|g_{K}\right\|}_{2}^{2}=Q\left({\bar{g}}_{K}^{*\lambda }\right)+\lambda {\left\|{\bar{g}}_{K}^{*\lambda }\right\|}_{2}^{2}.$$

Due to the positive definiteness of $\left({\bar{\Phi }}_{K}+\lambda I_{K,K}\right)$ for any λ>0, the unique solution to (30) is as follows:

(31) $${\bar{g}}_{K}^{*\lambda }={\left({\bar{\Phi }}_{K}+\lambda I_{K,K}\right)}^{-1}{\bar{\phi }}_{K},$$

which depends both on the functions defining the model $g_{K}^{T}\phi_{K}\left(t\right)$ (15), the weight function $\rho \left(t\right)$, and on the completely unknown real modulus $G\left(t\right)$, i.e., on the unknown spectra $H\left(\tau \right)$ and $H^{M}\left(v\right)$.

### 2.8. Random Selection of the Sampling Instants

By analogy to [53], we will assume that $T_{1},\ldots ,T_{N}$ are independent random variables with a common probability density function $\rho \left(t\right)$, with T being the support of $\rho \left(t\right)$. This means that the bounded non-negative weight function $\rho \left(t\right)$, applied in the definition of the integral $Q\left(g_{K}\right)$ (29), uniquely determines the probability distribution of the random variables $T_{1},\ldots ,T_{N}$. If the support T is bounded, i.e., $T=\left[t_{0},T\right]$, where T<∞, then we can assume a uniform distribution of the probability density function

$$\rho \left(t\right)=\frac{1}{T-t_{0}}1_{\left[t_{0},T\right]}\left(t\right),$$

where

$$1_{T}\left(t\right)=\left\{\begin{array}{c} 1\;\;\;\;\;\;if\;\;t\in T\\ 0\;\;\;\;\;\;if\;\;t\notin T \end{array}\right.,$$

is the indicator function (characteristic function) of a subset T of the set R. This means that, when defining the index $Q\left(g_{K}\right)$ (29), we give the same weight to the accuracy (error) of the modulus model for each t∈T. In turn, if the support $T=\left[t_{0},\right.\left.\infty \right)$, then the exponential distribution

$$\rho \left(t\right)=\beta e^{-\beta \left(t-t_{0}\right)}1_{\left[t_{0},\right.\left.\infty \right)}\left(t\right),$$

with the rate parameter β>0, can be taken. This means that the integral index $Q\left(g_{K}\right)$ attaches more importance to the relaxation modulus model error for small times, when the dynamics of the decreasing relaxation modulus is faster, than for large times, when the relaxation modulus decreases much slower.

Let the sampling times $t_{1},\ldots ,t_{N}$ applied in the stress relaxation experiment be selected from the set T independently according to the common probability distribution of the density function $\rho \left(t\right)$. This means that $t_{1},\ldots ,t_{N}$ are realizations of the random variables $T_{1},\ldots ,T_{N}$. The related modulus of the material $G\left(t_{i}\right)$ are realizations of random variables $G\left(T_{i}\right)$, i=1,…,N. No other assumptions about $\left\{t_{i}\right\}$ were made.

It is worth noting that the choice of function $\rho \left(t\right)$ affects the integral index $Q\left(g_{K}\right)$ (29) and affects the randomly generated sampling times $\left\{t_{i}\right\}$, but does not directly affect the definition of the empirical identification index $Q_{N}\left(g_{K}\right)$ (19), although the value of $Q_{N}\left(g_{K}\right)$ depends on $\rho \left(t\right)$ through the values of $\left\{t_{i}\right\}$. Consequently, the optimal model parameters ${\bar{g}}_{N,K}^{\lambda }$ (24) and ${\bar{g}}_{K}^{*\lambda }$ (31) depend on $\rho \left(t\right)$.

Let modulus measurements be disturbed by additive noises $Z_{i}$, for which, as in [53], the following assumption is taken as being natural for the modulus measurements:

**Assumption 5.**  *The noise $\left\{Z_{i}\right\}$ is, independent of the variables $\left\{T_{i}\right\}$, a sequence of independent identically distributed (i.i.d.) zero-mean random variables, $E\left[Z_{i}\right]=0$, having a common variance $E\left[Z_{i}^{2}\right]=\sigma^{2}<\infty$*.

Thus, for any i=1,…,N, the measurement noise $z\left(t_{i}\right)$, corrupting relaxation modulus measurement $\bar{G}\left(t_{i}\right)=G\left(t_{i}\right)+z\left(t_{i}\right)$, is a realization of the random variable $Z_{i}$.

## 3. Results and Discussion

The studies of the asymptotic properties of the identified relaxation spectra and modulus models, when the number of measurements tends to infinity, are conducted in this section. The convergence rate of these models to optimal sampling-times-independent models is estimated. The regularization parameter selection is discussed, and the generalized cross-validation rule is recommended. The algebraic background of computations using singular value decomposition is described. The identification algorithm is presented; some remarks concerning the stress relaxation experiment are also enclosed.

Next, analytically demonstrated asymptotic properties of the spectra models are confirmed by numerical studies. Three types of the “real” spectra, Kohlrausch–Williams–Watts, Gaussian-type, and Baumgaertel–Schausberger–Winter spectra, have been assumed. Both uni- and double-mode Gauss-like relaxation spectra are considered and used to describe the mechanical properties of materials, specifically polymers and biopolymers, examples of which are cited below. Based on the simulated experiment data, the optimal spectra models are identified using different series models defined by Bessel, Laguerre, and Legendre, as well as exponential functions. The convergence rate of the identified models to the sampling-times-independent model is analyzed and the noise robustness is studied.

### 3.1. The Optimal Model Convergence

With the replacement of the integral in $Q\left(g_{K}\right)$ (29) by a finite sum of squares of the model errors, index $Q_{N}\left(g_{K}\right)$ (19) is obtained, which is common practice in model identification.

For i=1,…,N, in view of Assumptions 5, the expected value

$$E{\left[G\left(T_{i}\right)+Z_{i}-G_{K}\left(T_{i},g_{K}\right)\right]}^{2}=Q\left(g_{K}\right)+\sigma^{2},$$

whence, by (19), the expected value

(32) $$E\left[Q_{N}\left(g_{K}\right)+\lambda {\left\|g_{K}\right\|}_{2}^{2}\right]=Q\left(g_{K}\right)+\lambda {\left\|g_{K}\right\|}_{2}^{2}+\sigma^{2}.$$

To directly explore the asymptotic properties of the regularized identification task (23), some results derived in [53] can be applied. As already indicated, all of the assumptions made in [53] are fulfilled here; the regularizer $R\left(g\right)$ from [53] is reduced here to the basis form of the Tikhonov regularizer, i.e., $R\left(g\right)=g^{T}g$.

Proposition 1 from [53], rewritten in terms of $Q_{N}\left(g_{K}\right)$ and $Q\left(g_{K}\right)$, implies the next proposition.

**Proposition 2.**  *Let *λ>0*. If Assumptions 1*−3 *and 5 hold, then*

(33) $$\underset{g_{K}\in G}{sup}\left|\left[Q\left(g_{K}\right)+\lambda {\left\|g_{K}\right\|}_{2}^{2}+\sigma^{2}\right]-\left[Q_{N}\left(g_{K}\right)+\lambda {\left\|g_{K}\right\|}_{2}^{2}\right]\right|\to 0\;w.p.1\;\;as\;N\to \infty ,$$

*with*w.p.1*meaning “with probability one”.*

Thus, the regularized empirical identification index in the optimization task (23) is arbitrarily close—uniformly in $g_{K}$ over the parameters set G (25)—to its measurement-independent expected value (compare (32)) as N→∞. This enables us to relate ${\bar{g}}_{N,K}^{\lambda }$, that solves the regularized identification task (23) for $Q_{N}\left(g_{K}\right)$, to ${\bar{g}}_{K}^{*\lambda }$, which minimize the regularized deterministic index $Q\left(g_{K}\right)$ in (30). Specifically, in view of the uniform in $g_{K}\in G$ convergence in (33) for the arbitrary λ>0, the following proposition can be concluded as being an analogue of Proposition 2 in [53]:

**Proposition 3.**  *Let* λ>0 *. If Assumptions 1–3 and 5 hold, and* $T_{1},\ldots ,T_{N}$ *are independently randomly selected from* T *, with the probability distribution having the density* $\rho \left(t\right)$ *, then*

(34) $${\bar{g}}_{N,K}^{\lambda }\to {\bar{g}}_{K}^{*\lambda }\;w.p.1\;\;as\;N\to \infty$$

*and for each* t∈T

(35) $$G_{K}\left(t,{\bar{g}}_{N,K}^{\lambda }\right)\to G_{K}\left(t,{\bar{g}}_{K}^{*\lambda }\right)\;w.p.1\;\;as\;N\to \infty .$$

*If, additionally, Assumption 4 holds, then for all* $\tau \geq \tau_{0}$

(36) $$H_{K}\left(\tau ,{\bar{g}}_{N,K}^{\lambda }\right)\to H_{K}\left(\tau ,{\bar{g}}_{K}^{*\lambda }\right)\;w.p.1\;as\;N\to \infty ,$$

*while, for all* $v\geq v_{0}$

(37) $$H_{K}^{M}\left(v,{\bar{g}}_{N,K}^{\lambda }\right)\to H_{K}^{M}\left(v,{\bar{g}}_{K}^{*\lambda }\right)\;w.p.1\;as\;N\to \infty .$$

The above result means that, under the taken assumptions, for any fixed λ>0, the parameter ${\bar{g}}_{N,K}^{\lambda }$ is a strongly consistent estimate of ${\bar{g}}_{K}^{*\lambda }$ being independent of the sampling times and the measurements ${\bar{G}}_{N}$. The model $G_{K}\left(t,g_{K}\right)$ (15), being linear with respect to $g_{K}$, according to Assumption 3, is Lipschitz on G uniformly in t∈T. Analogously, according to Assumption 4, the models $H_{K}\left(\tau ,g_{K}\right)$ (16) and $H_{K}^{M}\left(v,g_{K}\right)$ (17), also linear with respect to $g_{K}$, are Lipschitz on G uniformly in $\tau \geq \tau_{0}$ and $v\geq v_{0}$, respectively. Then, by the Schwartz inequality, the almost-sure convergence of ${\bar{g}}_{N,K}^{\lambda }$ to ${\bar{g}}_{K}^{*\lambda }$, stated in (34), implies that

(38) $$\underset{t\in T}{sup}\left|G_{K}\left(t,{\bar{g}}_{N,K}^{\lambda }\right)-G_{K}\left(t,{\bar{g}}_{K}^{*\lambda }\right)\right|\leq M_{2}{\left\|{\bar{g}}_{N,K}^{\lambda }-{\bar{g}}_{K}^{*\lambda }\right\|}_{2}\to 0\;w.p.1\;\;as\;N\to \infty ,$$

(39) $$\underset{\tau \geq \tau_{0}}{sup}\left|H_{K}\left(\tau ,{\bar{g}}_{N,K}^{\lambda }\right)-H_{K}\left(\tau ,{\bar{g}}_{K}^{*\lambda }\right)\right|\leq M_{\tau }{\left\|{\bar{g}}_{N,K}^{\lambda }-{\bar{g}}_{K}^{*\lambda }\right\|}_{2}\to 0w.p.1\;\mathrm{as}\;N\to \infty ,$$

and

(40) $$\underset{v\geq v_{0}}{sup}\left|H_{K}^{M}\left(v,{\bar{g}}_{N,K}^{\lambda }\right)-H_{K}^{M}\left(v,{\bar{g}}_{K}^{*\lambda }\right)\right|\leq M_{v}{\left\|{\bar{g}}_{N,K}^{\lambda }-{\bar{g}}_{K}^{*\lambda }\right\|}_{2}\to 0\;w.p.1\;\;as\;N\to \infty ,$$

where the constant $M_{2}$ is defined in Assumption 3, while $M_{\tau }$ and $M_{v}$ are introduced in Assumption 4.

Thus, according to (38), model $G_{M}\left(t,{\bar{g}}_{N,K}^{\lambda }\right)$ is a strongly uniformly consistent estimate of the relaxation modulus model with the parameter ${\bar{g}}_{K}^{*\lambda }$ (31). This means that, when Assumptions 1–3 and 5 hold, the arbitrarily precise approximation of the best model $G_{K}\left(t,{\bar{g}}_{K}^{*\lambda }\right)$ is achievable (almost everywhere) as N grows significantly, although the true modulus characteristic is unknown.

In view of (39) and (40), models $H_{K}\left(\tau ,{\bar{g}}_{N,K}^{\lambda }\right)$ and $H_{K}^{M}\left(v,{\bar{g}}_{N,K}^{\lambda }\right)$ are strongly uniformly consistent estimates of the models with ${\bar{g}}_{K}^{*\lambda }$ (31), and the arbitrarily precise approximations can be obtained for N growing to infinity when Assumptions 1–5 are in force. However, models $H_{K}\left(\tau ,{\bar{g}}_{K}^{*\lambda }\right)$ and $H_{K}^{M}\left(v,{\bar{g}}_{K}^{*\lambda }\right)$ do not necessarily provide the best approximation to the real relaxation spectra in the sense of any specific measure related directly to the unknown spectra. These models are the best in the sense of the regularized index $Q\left(g_{K}\right)$, which is related to the relaxation modulus as being available by experimentation. However, relating the model quality indices to measurement-available process variables is common for the inverse problems of rheology, both when the stress relaxation experiment data [13,14,15,16,17,40] and the dynamic moduli measurements [4,19,20,21,22,23,24,25,26,27,28,29] are used for identification. Therefore, according these methods, the quality of the spectra models must be evaluated only in an indirect manner, using the measures related to the relaxation dynamic moduli models. Recently, a different approach was used in [32] that used approximation of the real spectrum by a series of functions, and applied an integral square error between the real spectrum and its model as a measure of the model quality. The basis functions considered in [32] are discussed in Appendix A.2. However, the concept of direct spectra identification [32] is an exception in the relaxation spectra identification area and cannot be applied for sampling-times-independent model identification.

### 3.2. Rate of Convergence

The analysis of the convergence rate in (34)–(37) is based on the assessment made in [53] of the speed of the convergence of the parameter ${\bar{g}}_{N,K}^{\lambda }$ to ${\bar{g}}_{K}^{*\lambda }$ as N grows. Following [53], we examine the disagreement between ${\bar{g}}_{N,K}^{\lambda }$ and ${\bar{g}}_{K}^{*\lambda }$ by examining how fast, for an assumed ε>0, the probability $P\left\{J\left({\bar{g}}_{N,K}^{\lambda },{\bar{g}}_{K}^{*\lambda }\right)\geq \varepsilon \right\}$ strives to zero as N increases, where the index

$$J\left({\bar{g}}_{N,K}^{\lambda },{\bar{g}}_{K}^{*\lambda }\right)=\left|\left[Q\left({\bar{g}}_{N,K}^{\lambda }\right)+\lambda {\left\|{\bar{g}}_{N,K}^{\lambda }\right\|}_{2}^{2}\right]-\left[Q\left({\bar{g}}_{K}^{*\lambda }\right)+\lambda {\left\|{\bar{g}}_{K}^{*\lambda }\right\|}_{2}^{2}\right]\right|,$$

is defined by difference in the regularized integral identification index.

In [53] (Appendix A.1), for any ε>0 and any λ>0, the following estimation is given:

(41) $$P\left\{J\left({\bar{g}}_{N,K}^{\lambda },{\bar{g}}_{K}^{*\lambda }\right)\geq \varepsilon \right\}\leq 2exp\left(\frac{-N\varepsilon^{2}}{8{\hat{M}}^{2}}\right),$$

where the positive constant

(42) $$\hat{M}=2\left(1+\delta \right)\left(M_{1}+M_{4}\right)+\sigma^{2}+\delta^{2}$$

characterizes how N and the disturbance parameters affect the convergence speed; the constant $M_{1}$, the upper bound δ, and the noise variance σ are introduced in Assumptions 1, 2, and 5, respectively, and $M_{4}$ is defined in Equation (27). In particular, for fixed ε, the bounds for $P\left\{J\left({\bar{g}}_{N,K}^{\lambda },{\bar{g}}_{K}^{*\lambda }\right)\geq \varepsilon \right\}$ exponentially decline to zero when N grows. The convergence rate is the higher, while the lower is $\hat{M}$ (42), i.e., the measurement noises are weaker. In turn, according to (42), for stronger disturbances, the rate of the probability convergence is reduced. The larger δ and σ are, the greater the speed drop. This is expected, since, with large noises, the measurements are not very accurate. Similarly, the larger the $M_{1}+M_{4}$, i.e., by inequality (28), the greater the inaccuracy between the real modulus and the model $G_{K}\left(t,g_{K}\right)$, the worse the convergence.

### 3.3. Selecting the Regularization Parameter

Both the integral (30) and empirical (23) regularized approximation tasks are linear quadratic optimization problems, with the solutions given by Equations (31) and (24), respectively. These minimization tasks and their solutions depend on the regularization parameter. It is commonly accepted that, for ill-posed problems, an appropriate choice of the respective regularization parameter is important to identify the satisfactory model. There are well-studied techniques for computing a good regularization parameter, being generalized cross-validation [8,41] and L-curve techniques [8], which are probably the most frequently used and described in detail in the literature. Both of these techniques can be used for our problems, in particular for the empirical task (23). In the previous paper [53], the rule of guaranteed model approximation is considered, which was first applied by Stankiewicz [57] for the identification of the relaxation time spectrum; for a description, see [57] or [53]. Here, for the numerical studies, the generalized cross-validation (GCV) method is applied to decide on a suitable choice for the regularization parameter, which does not depend on a priori knowledge about the noise variance, and has previously been successfully used for the relaxation time [17] and frequency [16] spectra. For details, we refer the reader to the source papers [8,41] or descriptions of the GCV technique application to identifying the relaxation spectra [16,17]. When designing the algorithm described below, it was assumed that the regularization parameter was selected only once for the selected value of the number of measurements, and used for the subsequent values of N. However, a re-selection can also be made for another different value of N if the identification results—measured, for example, by the empirical index $Q_{N}\left(g_{K}\right)$ (19)—prove to be unsatisfactory. This new regularization parameter should be kept for further increasing values of N to guarantee model convergence to measurement-independent models. In the numerical studies, the regularization parameter, once selected, was kept constant to ensure the validity of the convergence results (34)–(37).

### 3.4. Algebraic Background of Computations

Let the singular value decomposition (SVD) of the N×K-dimensional matrix $\Phi_{N,K}$ be as follows [58]:

(43) $$\Phi_{N,K}=U\;\Sigma \;V^{T},$$

with diagonal matrix $\Sigma =diag\left(\sigma_{1},\ldots ,\sigma_{r},0,\ldots ,0\right)\epsilon R^{N,K}$ containing the non-zero singular values $\sigma_{1},\ldots ,\sigma_{r}$ of $\Phi_{N,K}$ [58], and the orthogonal matrices $V\in R^{K,K}$ and $U\in R^{N,N}$, where $r=rank\left(\Phi_{N,K}\right)\leq K$. The diagonal structure of Σ, jointly with the orthogonality of matrices V and U, imply the following formula for the regularized optimal parameter ${\bar{g}}_{N,K}^{\lambda }$ (24):

(44) $${\bar{g}}_{N,K}^{\lambda }=V{\left(\Sigma^{T}\Sigma +\lambda NI_{K,K}\right)}^{-1}\Sigma^{T}U^{T}\;{\bar{G}}_{N}={V\Lambda }_{\lambda }\;U^{T}\;{\bar{G}}_{N},$$

where K×N matrix $\Lambda_{\lambda }$ is diagonal, as follows:

$$\Lambda_{\lambda }=diag\left(\sigma_{1}/\left(\sigma_{1}^{2}+\lambda N\right),\ldots ,\sigma_{r}/\left(\sigma_{r}^{2}+\lambda N\right),0,\ldots ,0\right).$$

Applying the generalized cross-validation to the regularized solution ${\bar{g}}_{N,K}^{\lambda }$ (24) relies on choosing the regularization parameter λ that minimizes the GCV function [8,41]:

(45) $$V_{GCV}\left(\lambda \right)=\frac{{\left\|\varrho \left(\lambda \right)\right\|}_{2}^{2}}{{tr\left[\Xi \left(\lambda \right)\right]}^{2}},$$

where $tr\left[\Xi \left(\lambda \right)\right]$ denotes the trace of the square K×K matrix defined as follows:

$$\Xi \left(\lambda \right)={I_{N,N}-\Phi_{N,K}\left(\Phi_{N,K}^{T}\Phi_{N,K}+\lambda {NI}_{K,K}\right)}^{-1}\Phi_{N,K}^{T},$$

and the residual vector $\varrho \left(\lambda \right)$ for (24) is given by

$$\varrho \left(\lambda \right)=\Xi \left(\lambda \right){\bar{G}}_{N}={\bar{G}}_{N}-\Phi_{N,K}{\bar{g}}_{N,K}^{\lambda }.$$

The problem of choosing the optimal regularization parameter is as follows:

(46) λGCV=minλ: λ=arg minλ≥0VGCVλ.

Using SVD (43), the GCV function (45) can be expressed as

$$V_{GCV}\left(\lambda \right)=\left[\sum_{i=1}^{r}\frac{\lambda^{2}N^{2}y_{i}^{2}}{{\left(\sigma_{i}^{2}+\lambda N\right)}^{2}}+\sum_{i=r+1}^{N}\;y_{i}^{2}\right]/{\left[N-r+\sum_{i=1}^{r}\frac{\lambda N}{\left(\sigma_{i}^{2}+\lambda N\right)}\right]}^{2},$$

where $y_{i}$ compose the N-dimensional vector $Y=U^{T}{\bar{G}}_{N}$.

### 3.5. Identification Algorithm

Based on (34)–(37), the calculation of the approximate value ${\bar{g}}_{N,K}^{\lambda }$ of the best model parameter ${\bar{g}}_{K}^{*\lambda }$ includes the following steps:

1. Perform the preliminary stress relaxation test [1,2,3] and record the relaxation modulus measurements $\bar{G}\left(t_{i}\right)$, i=1,…,N, for preselected time instants $t_{i}$, for example, those taken with a constant sampling period.
2. Choose the model parameters α and K, comparing, for different values of α, a few functions from the sequence $\left\{\phi_{k}\left(t\right)\right\}$ of the basis functions creating model $G_{K}\left(t,g_{K}\right)$ (15) with the measurements $\left\{\bar{G}\left(t_{i}\right)\right\}$. Set p=1.
3. Randomly select sampling times $t_{1},\ldots ,t_{N}$, generating each $t_{i}$ independently from T according to the probability distribution with density $\rho \left(t\right)$, identical with the weighting function assumed in $Q\left(g_{K}\right)$ (29).
4. Run the stress relaxation test and collect the relaxation modulus measurements $\bar{G}\left(t_{i}\right)$ at the times $t_{i}$, i=1,…,N.
5. Select the regularization parameter λ for the regularized optimal identification task (23) using the selected rule.
6. Compute parameter ${\bar{g}}_{N,K}^{\lambda }$ using the Equation (24).
7. If N is the first number of the measurements used, go to step 9. Otherwise, go to step 8.
8. In order to ascertain if ${\bar{g}}_{\bar{N},K}^{\lambda }$ satisfactorily approximates ${\bar{g}}_{K}^{*\lambda }$, check if ${\left\|{\bar{g}}_{\bar{N},K}^{\lambda }-{\bar{g}}_{N,K}^{\lambda }\right\|}_{2}<\varepsilon_{1}$ for the small positive $\varepsilon_{1}$. If not, go again to step 9. Otherwise, stop the iterations and take ${\bar{g}}_{\bar{N},K}^{\lambda }$ as the approximation of ${\bar{g}}_{K}^{*\lambda }$.
9. Put $\bar{N}=N$ and ${\bar{g}}_{\bar{N},K}^{\lambda }={\bar{g}}_{N,K}^{\lambda }$. Select the new $N\gg \bar{N}$ to expand the measurements data set.
10. For the new N, repeat steps 3, 4, 6, and 8; that is, select new sampling times, perform again the stress relaxation test for the next sample of the same material, and compute the new model parameter ${\bar{g}}_{N,K}^{\lambda }$ given by (24) for the previously selected regularization parameter λ.

For parameter ${\bar{g}}_{K}^{*\lambda }$, the best models $G_{K}\left(t,{\bar{g}}_{K}^{*\lambda }\right)$, $H_{K}\left(\tau ,{\bar{g}}_{K}^{*\lambda }\right)$, and $H_{K}^{M}\left(v,{\bar{g}}_{K}^{*\lambda }\right)$ are given by Equations (15), (16), and (17), respectively.

**Figure 1:**
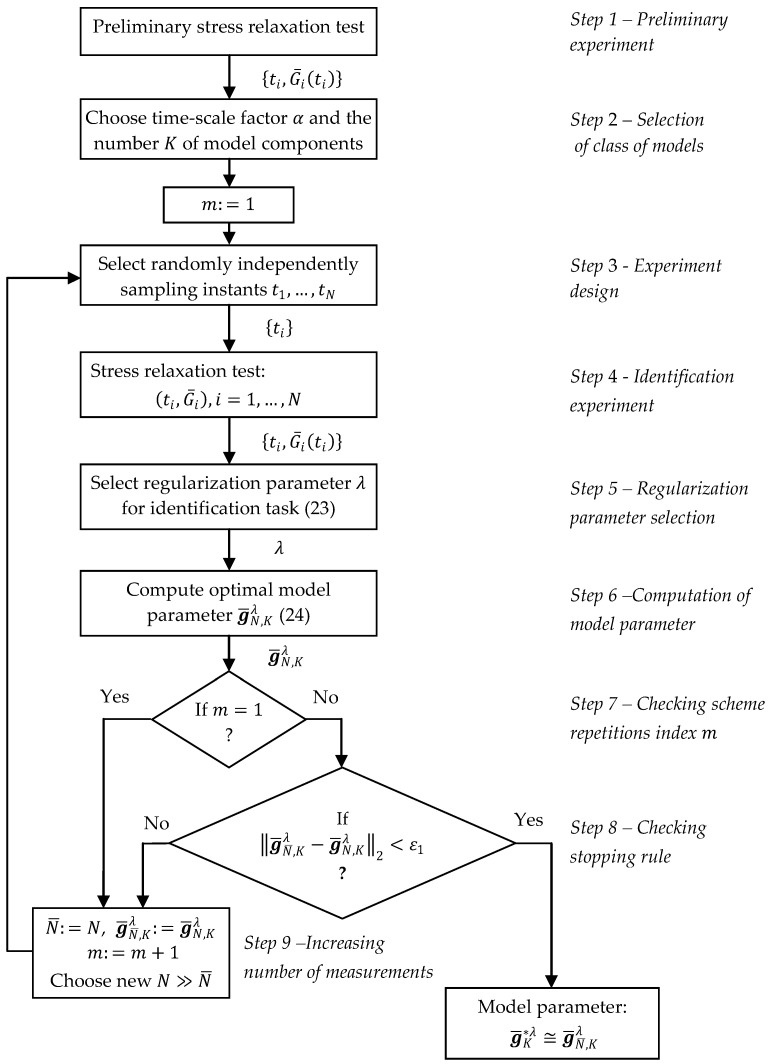
The block diagram of the algorithm for the relaxation spectra model identification.

A schematic flow diagram of the identification procedure, illustrating the communication between the steps of determining the optimal parameters of the regularized model and performing the relaxation test for subsequent numbers of measurements N, is shown in Figure 1. The additional integer variable m is the index of the repetitions of the stress relaxation experiment.

### 3.6. Remarks

1. Preliminary steps 1 and 2, which consist in selecting the time-scaling factor α and the number of model components K, are typical for approximation algorithms based on the expansion of the model into function series. The details of this technique have been discussed earlier in [16,32,40]. The two-level scheme of the optimal time-scale factor selection presented in [17] can also be applied here.
2. According the approach proposed, the measurements $\bar{G}\left(t_{i}\right)$ of the relaxation modulus at the sampling instants $t_{1},\ldots ,t_{N}$ randomly selected in step 3 are needed for model identification. They must be measured and recorded in the stress relaxation test performed in step 4. However, modern testing machines offer very high data acquisition rates, e.g., Instron Universal Testing Systems, Instron Worldwide, Norwood, MA, USA: 6800 Series—up to 5000 Hz, 3400 Series and Industrial Series—up to 1000 Hz, and 5980 Series—up to 2500 Hz, while in the case of Zwick/Roell ProLine Series Testing Machines, ZwickRoell GmbH & Co. KG, Ulm, Germany, the sampling rate is up to 2000 Hz. Therefore, in practice, it is enough to select from such a densely sampled and recorded large data set only those data $\bar{G}\left(t_{i}\right)$ that correspond to previously randomly selected sampling times $t_{i}$, and then use only these measurement data for further model identification.
3. In step 5, the formulae needed for the selection of the regularization parameter are not specified so to leave the user free to choose their favorite method. The application of the GCV method was discussed in earlier works on relaxation spectra identification [16,17]. The parameter λ is chosen only once (step 5) and then used for the next measurements $\left\{\bar{G}\left(t_{i}\right)\right\}$ whenever steps 3, 4, 6, and 8 are repeated.
4. For the Equation (24) computations, the SVD (43) can be used, as described in previous papers [16,17,40], resulting in Equation (44).
5. The stopping rule (step 8) can be changed to a less restrictive one based on applying index $Q_{N}\left(g_{K}\right)$ (20), i.e., on examining whether $\left|Q_{\bar{N}}\left({\bar{g}}_{\bar{N},K}^{\lambda }\right)-Q_{N}\left({\bar{g}}_{N,K}^{\lambda }\right)\right|<\varepsilon_{1}$ is satisfied.

### 3.7. Applicability to the Models Using Expansions into Different Basis Series

It is demonstrated in Appendix A.1, Appendix A.2, Appendix A.3 and Appendix A.4 and Supplementary Materials S.1 and S.2 that known relaxation spectra models based on the exponential, power-exponential, and the Laguerre, Legendre, and Chebyshev functions satisfy Assumptions 3 and 4 related to the models applied for identification.

The choice of specific basis functions has been discussed in detail in previous articles [16,17,18,32,40], which presented the applicability ranges of particular classes of models. Some basis functions can be successfully used for small and even very small relaxation times/frequencies, while others are recommended for large and very large relaxation times/frequencies. For orthogonal basis functions (Laguerre, Legendre, Hermite, and Chebyshev), detailed information can be found in [16], Section 3.7 ‘Choice of the Basis Functions’, and especially in [16], (Table A1), where the applicability ranges in terms of the relaxation frequency v are given, ranging from two to five decades for different values of the time-scale factor α and the number of model summands K. The use of different types of exponential functions is described in the articles by [17,32,40]. The possibility of directly applying exponential functions to modeling the relaxation spectra of different types is characterized in [32], Section 3.11. The application of the power-exponential functions of the relaxation time spectrum, for which the basis functions of the modulus use modified Bessel functions of the second kind, is discussed in [17], Section 2.2.5; the respective applicability ranges of the relaxation times, which reach up to six decades, depending on the time-scale factor and number of model summands, are specified in [17], Tables 1 and A1. In [40], Section 2.3.4 is devoted to the suitability of power-square-exponential basis functions for modeling the frequency spectrum when the modulus is described by the products of power of time and complementary error functions; the corresponding relaxation frequency ranges are given in [4], Tables 1 and B1. These ranges reach up to five decades depending on the model parameters α and K.

The numerical results of simulation studies conducted for uni- and double-mode spectra with short, medium, and long relaxation times, presented in the following sections, validate the applicability. The choice of specific basis functions in the examples presented below stems from previous studies that have already used these classes of models for these spectra. In this paper, we extended the spectra modeling scheme to include randomly selected sampling times. Therefore, we investigated the asymptotic properties of the models without focusing on the accuracy of the models themselves. This implies that better spectra models might exist if a different class of models were used. However, determining this was not the aim of this paper.

### 3.8. Simulated Materials

Below, simulation results are presented regarding the applicability of the proposed approach for three types of relaxation spectra: Kohlrausch–Williams–Watts (KWW), Baumgaertel–Schausberger–Winter (BSW), and uni- and double-mode Gaussian spectra.

The KWW model, describing stretched exponential relaxation, can model viscoelastic properties of, e.g., polymer melts [59], bone and bone collagen [60], annealed metallic and polymer glasses [61], and many other materials. Examples of other KWW-type relaxation processes occurring in materials are given in [32].

The Gaussian-like relaxation distributions have been used to describe the rheological characteristics of many polymers [62,63,64]; references to Gaussian spectra of biopolymers are discussed in [32]. Gaussian spectra illustrate the usefulness of new identification methods in [24,25,46] and also in our studies [16,17,32,40,53].

The material described by the Baumgaertel, Schausberger, and Winter (BSW) spectrum [21,65] is also considered, which is often used to describe the viscoelasticity of long-relaxation-time polymers [42,43,66,67].

This means that the short times of KWW relaxation, the medium times of the Gaussian-type relaxation, and the long times of BSW relaxation are taken into account.

### 3.9. Simulational Studies

First, for each simulated material, the assumed class of models, and the weakest noises, the preliminary stress relaxation experiment was performed for the preselected N. Then, the time-scale factor α and the number of model summands K were taken by comparing the courses of recorded $\left\{\bar{G}\left(t_{i}\right)\right\}$ and functions $\phi_{k}\left(t\right)$ (13), as well as (14) for 1≤k≤K and a few K (steps 1 and 2). Only for the KWW spectrum example, the time-scaling factor was optimally selected using the scheme presented in [17].

The real materials and functions $h_{k}\left(v\right)$ and $\phi_{k}\left(t\right)$ were simulated in Matlab R2023b, The Mathworks, Inc., Natick, MA, USA, with the application of the special functions *erfc*, *legendreP, besselk*, and *laguerreL*; the *svd* procedure was also applied.

#### 3.9.1. Studies of the Asymptotic Properties

Next, for each simulated material, assumed class of models, and the pair $\left(N,\sigma \right)$, the simulated relaxation test was performed for sampling instants $t_{1},\ldots ,t_{N}$, selected randomly according to the uniform distribution on T, and randomly generated measurement noises $z_{i}$ (steps 3 and 4). However, for the double-mode Gauss-like relaxation spectrum, Assumption 3 holds for any t>0, and for the other simulated spectra is satisfied for every t≥0. In the numerical simulations, finite time intervals $T=\left[t_{0},T\right]$ were assumed, with the upper limit T depending on the rheological properties of the material (characterized below), because this implies a constant weight function $\rho \left(t\right)$ for the integral index $Q\left(g_{K}\right)$ (29) and an appropriate uniform distribution used for sampling $t_{i}$. A normal distribution with zero mean and variance $\sigma^{2}$ was used to independently generate disturbances $\left\{z_{i}\right\}$.

Using measurement data obtained for N=100, the parameter λ was chosen by the GCV rule (46), i.e., $\lambda =\lambda_{GCV}$ (step 5). Next, for any applied number of measurements, the regularized optimal model parameter ${\bar{g}}_{N,K}^{\lambda }$ (24) was computed (step 6). The SVD technique is applied for numerical computations. The optimal spectra $H_{K}\left(\tau ,{\bar{g}}_{N,K}^{\lambda }\right)$ (9) and $H_{K}^{M}\left(v,{\bar{g}}_{N,K}^{\lambda }\right)$ (10) and modulus $G_{K}\left(t,{\bar{g}}_{N,K}^{\lambda }\right)$ (11) models were determined, and are presented in the figures below and in the Supplementary Materials. For successive values of N, the indices $Q_{N}\left({\bar{g}}_{N,K}^{\lambda }\right)$ (19) and $Q\left({\bar{g}}_{N,K}^{\lambda }\right)$ (29), and the Euclidean norm of the parameter ${\bar{g}}_{N,K}^{\lambda }$ (compare (A26)), are determined and given in successive tables. The dimensionless mean relative error of the real modulus approximation

(47) $$Q_{Nrel}\left(g_{K}\right)=\frac{1}{N}\sum_{i=1}^{N}{\left[\frac{{G\left(t_{i}\right)-G}_{K}\left(t_{i},g_{K}\right)}{G\left(t_{i}\right)}\right]}^{2}=\frac{1}{N}\sum_{i=1}^{N}{\left[1-\frac{G_{K}\left(t_{i},g_{K}\right)}{G\left(t_{i}\right)}\right]}^{2},$$

where $G\left(t_{i}\right)$ is the real modulus, is also determined and given in these tables; they are expressed as percentages. The above index allows for the assessment of the model fit regardless of the value of the real modulus.

For successive N, the quality of the optimal sampling-times-independent ${\bar{g}}_{K}^{*\lambda }$ (31) approximation is measured by the following relative percentage error:

(48) $$ERR={\left\|{\bar{g}}_{N,K}^{\lambda }-{\bar{g}}_{K}^{*\lambda }\right\|}_{2}^{2}/{\left\|{\bar{g}}_{K}^{*\lambda }\right\|}_{2}^{2}\cdot 100\%.$$

#### 3.9.2. Noise Robustness Analysis

To examine the noise robustness of the regularized identification algorithm, for each pair $\left(N,\sigma \right)$, the simulation experiment was repeated n=50 times with $\left\{z_{i}\right\}$ generated, in each repetition, independently and randomly with the zero-mean normal distribution of variance $\sigma^{2}$. Three variances for any simulated material are used.

To generalize the relaxation modulus empirical approximation error $Q_{N}\left(g_{K}\right)$ (19) for the n-element sample, we define

(49) $$ERRQ_{N}=\frac{1}{n}\sum_{j=1}^{n}Q_{N}\left({\bar{g}}_{N,K,j}^{\lambda }\right),$$

where ${\bar{g}}_{N,K,j}^{\lambda }$ denotes parameter (24) computed for the *j*-th experiment repetition for $\left(N,\sigma \right)$, j=1,…,n.

Analogously, the mean integral index

(50) $$ERRQ=\frac{1}{n}\sum_{j=1}^{n}Q\left({\bar{g}}_{N,K,j}^{\lambda }\right)$$

was defined and computed for each pair $\left(N,\sigma \right)$, where $Q\left(g_{K}\right)$ is defined by (29).

The next index generalizes the relative error ERR (48), estimating the distance between vectors ${\bar{g}}_{N,K,j}^{\lambda }$ and ${\bar{g}}_{K}^{*\lambda }$ (31), for an n element sample as follows:

(51) $$MERR=\frac{1}{n}\sum_{j=1}^{n}{\left\|{\bar{g}}_{N,K,j}^{\lambda }-{\bar{g}}_{K}^{*\lambda }\right\|}_{2}^{2}/{\left\|{\bar{g}}_{K}^{*\lambda }\right\|}_{2}^{2}\cdot 100\%$$

where this is a measure of the sampling-times-independent parameter ${\bar{g}}_{K}^{*\lambda }$ approximation.

The mean square norm of the regularized optimal model parameter (24) was evaluated by index

(52) $$MNORM=\frac{1}{n}\sum_{j=1}^{n}{\left\|{\bar{g}}_{N,K,j}^{\lambda }\right\|}_{2}$$

as a measure of the parameter vector smoothness.

### 3.10. KWW Relaxation Spectrum

The KWW model [68,69]

(53) $$G\left(t\right)=G_{0}e^{-{\left(\frac{t}{\tau_{r}}\right)}^{\beta }},$$

with the stretching exponent 0<β<1, where $\tau_{r}$ and $G_{0}>0$ are the relaxation time and the initial shear modulus, has a uni-modal relaxation time spectrum given by the infinite series [68,69]

$$H\left(\tau \right)=\frac{G_{0}}{\pi }\;\sum_{k=1}^{\infty }\frac{{\left(-1\right)}^{k+1}}{k!}\sin\left(\pi \beta k\right)\;\Gamma \left(\beta k+1\right)\;{\left(\frac{\tau }{\tau_{r}}\right)}^{\beta k}.$$

Assumption 1, concerning the boundness of the real modulus, is satisfied for $T=\left[0,T\right]$, where an arbitrary T>0.

For β=0.5, spectrum $H\left(\tau \right)$ is described by the following simple formula [32,68]:

(54) $$H\left(\tau \right)=\frac{G_{0}}{2\sqrt{\pi }}\sqrt{\frac{\tau }{\tau_{r}}}\;e^{-\frac{\tau }{4\tau_{r}\;}},$$

which we will take here following [32]. According to the right equation in (3) and (4), the modified spectrum corresponding to (54)

(55) $$H^{M}\left(v\right)=\frac{G_{0}}{2\sqrt{\pi }}\sqrt{\frac{1}{\tau_{r}\cdot v^{3}}}e^{-\frac{1}{4\tau_{r}\cdot v}}.$$

The exponent β=0.5 has been reported, for example, for some polymers [70] and silicate glasses [71] (Table I). The exponents β close to 0.5 have been also obtained for some polymers and glass forms [70,71]. Chen et al. [72], for modeling relaxation in crosslinked polystyrene, used the KWW model of the following parameters: $G_{0}=0.78\;MPa$, β=0.59, and $\tau_{r}=1.08\;s$ [72]. Following [32], for our studies, we substitute β=0.59 with β=0.5 (for the justification, see [32]).

Following [32], relaxation spectra model $H_{K}^{M}\left(v,g_{K}\right)$ (10) using exponential functions $h_{k}\left(v\right)$ (A9) are assumed, which means the approximation of the modulus by models $G_{K}\left(t,g_{K}\right)$ (12) composed by functions $\phi_{k}\left(t\right)$ (A11). According to Property A.2, Assumption 3 is satisfied for any t≥0 and Assumption 4 holds for any relaxation frequency v≥0.

Based on the previous studies [32], bounded interval $T=\left[0,\;50\right]$ seconds is adopted to apply the weight function $\rho \left(t\right)=\frac{1}{50}1_{\left[0,\;50\right]}\left(t\right)s^{-1}$. Following [32], N=400 sampling instants were generated with the constant period in $=\left[0,\;50\right]\;s$ in the preliminary experiment. A simulated stress relaxation test was performed for KWW material $G\left(t\right)$ (53); the measurement noises $z\left(t_{i}\right)$ were generated randomly according to the zero-mean normal distribution of standard deviation σ=0.0005 MPa. K=20 model summands were adopted, and the time-scale factor α=1.97 s of the functions $h_{k}\left(v\right)$ (A9) were optimally selected by applying the scheme presented in [16].

Next, for N=50, the sampling times $t_{1},\ldots ,t_{N}$ were generated from the set T according to the uniform distribution of the density function $\rho \left(t\right)=\frac{1}{50}1_{\left[0,\;50\right]}\left(t\right)$, and a simulated stress relaxation test was performed for normal distribution noises of standard deviation σ=0.0005 MPa. Applying the GCV rule, the regularization parameter $\lambda =8.3\times 10^{-8}\;s^{-2}$ was selected (step 5) and, next, used together with α=1.97 s and K=20 for the subsequent simulations.

#### 3.10.1. Asymptotic Properties

The experiment was repeated for eighteen values of N, varying from 20 to 10,000, and σ=0.0005, 0.001, 0.003 MPa of the noises. The indices $Q_{N}\left({\bar{g}}_{N,K}^{\lambda }\right)$ (19), $Q\left({\bar{g}}_{N,K}^{\lambda }\right)$ (29), ${\left\|{\bar{g}}_{N,K}^{\lambda }\right\|}_{2}$, and ERR (48) were determined, and are given in Table 1 for the weakest noises, and in Tables S1 and S2, moved to the Supplementary Materials, for intermediate and strong noises.

**Figure 2:**
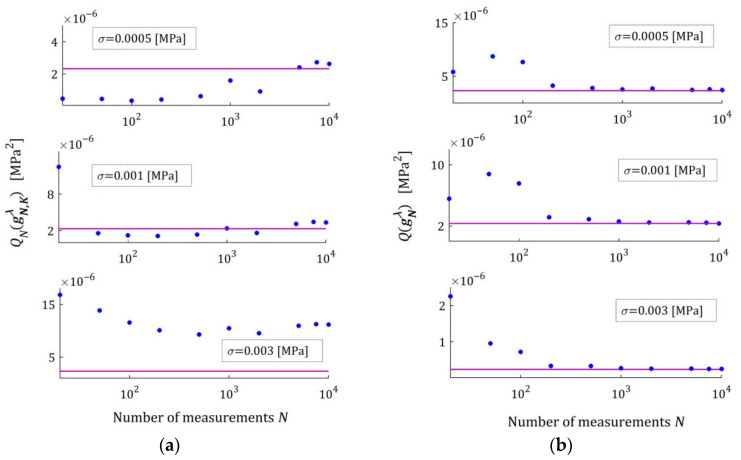
The identification indices (blue dots) of the KWW modulus $G\left(t\right)$ (53) approximation by model $G_{K}\left(t,g_{K}\right)$ (12) with basis functions $\phi_{k}\left(t\right)$ (A11), corresponding to exponential functions $h_{k}\left(v\right)$ (A9) of the time-scale factor α=1.97 s and K=20 model components, determined for the regularization parameter $\lambda =8.3\times 10^{-8}\;s^{-2}$: (**a**) empirical $Q_{N}\left({\bar{g}}_{N,K}^{\lambda }\right)$ (19); (**b**) integral $Q\left({\bar{g}}_{N,K}^{\lambda }\right)$ (29) for an increasing number of measurements N and the noises σ=0.0005, 0.001, 0.003 MPa. The horizontal purple lines mark the optimal integral index $Q\left({\bar{g}}_{K}^{*\lambda }\right)$ defined in Equation (30).

Figure 2 illustrates the asymptotic properties by juxtaposing the indices $Q_{N}\left({\bar{g}}_{N,K}^{\lambda }\right)$ (19) and $Q\left({\bar{g}}_{N,K}^{\lambda }\right)$ (29) as functions of N for the noises considered; the optimal index $Q\left({\bar{g}}_{K}^{*\lambda }\right)=2.324634\times 10^{-6}$ ${MPa}^{2}$ is marked with violet horizontal lines; a logarithmic scale is applied on the horizontal axes. In these and subsequent figures, the makron over the vectors ${\bar{g}}_{N,K}^{\lambda }$ has been excluded to simplify the description of the vertical axes. The values $Q_{N}\left({\bar{g}}_{N,K}^{\lambda }\right)$ of the empirical index tend towards the constant value only for N≥5000, while the values $Q\left({\bar{g}}_{N,K}^{\lambda }\right)$ of the integral index noticeably stabilize already for N≥500. It is this index, being independent of the specific experimental results, that is the main measure of the model quality. Thus, the asymptotic properties of the identification algorithm were confirmed. Already for N≥500 measurements, the relative error of approximation of the real modulus $Q_{Nrel}\left({\bar{g}}_{N,K}^{\lambda }\right)$ does not exceed 0.4%; for the weakest noises, even for N≥200.

**Figure 3:**
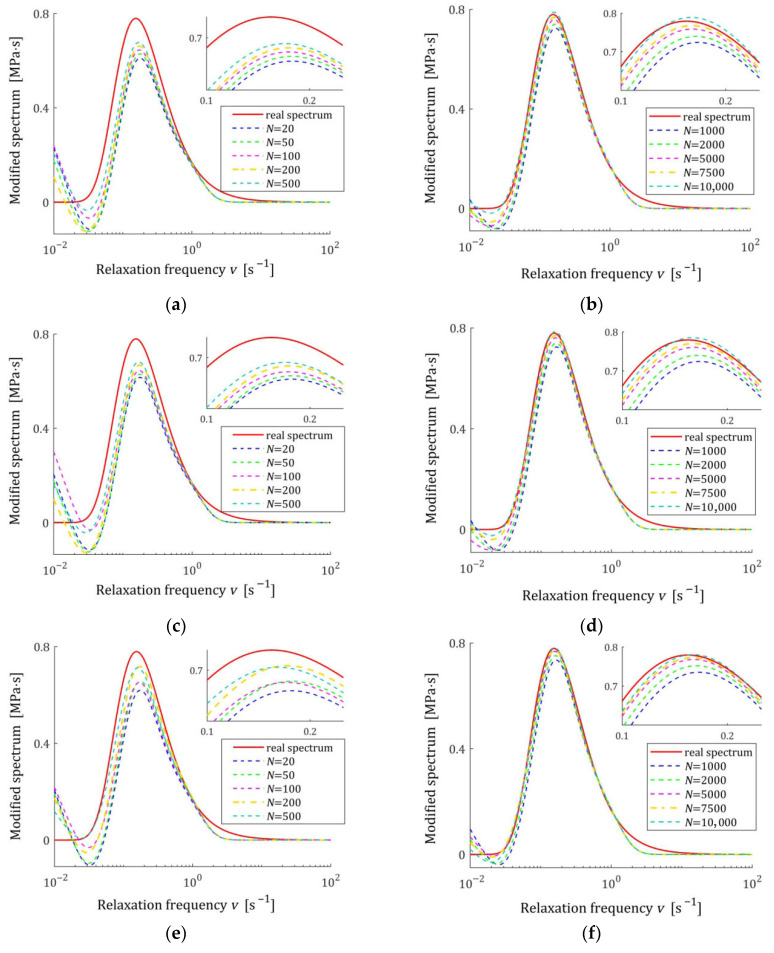
Modified KWW relaxation frequency spectrum $H^{M}\left(v\right)$ (55) and the approximated models $H_{K}^{M}\left(v,{\bar{g}}_{N,K}^{\lambda }\right)$ (10), with exponential basis functions $h_{k}\left(v\right)$ (A9) of the time-scale factor α=1.97 s and K=20 model components, determined for the regularization parameter $\lambda =8.3\times 10^{-8}\;s^{-2}$ for selected values of N and noises of standard deviations: (**a**,**b**) σ=0.0005 MPa; (**c**,**d**) σ=0.001 MPa; (**e**,**f**) σ=0.003 MPa.

The spectra models $H_{K}^{M}\left(v,{\bar{g}}_{N,K}^{\lambda }\right)$, given by Equation (10), were determined and plotted in Figure 3 for σ=0.0005, 0.001, 0.003 MPa. We see that increasing the number of measurements noticeably improves the relaxation spectra models, which increasingly approximate the real spectrum even for the strongest disturbances.

**Figure 4:**
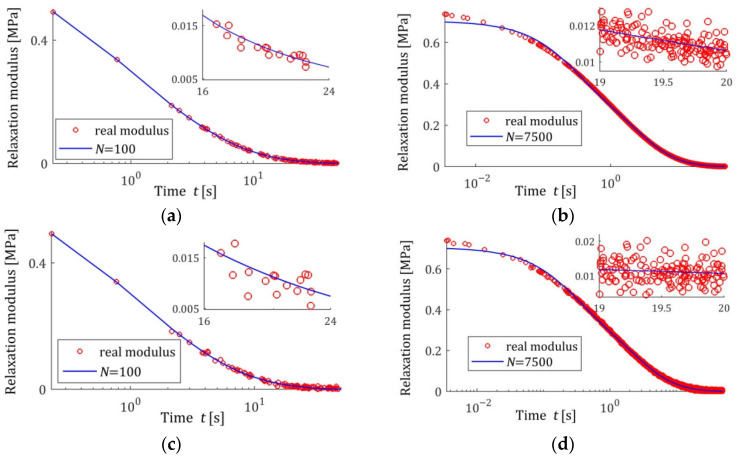
The KWW relaxation modulus $G\left(t\right)$ (53) measurements $\bar{G}\left(t_{i}\right)$(red points) and the series models $G_{K}\left(t,{\bar{g}}_{N,K}^{\lambda }\right)$ (12) (blue lines), with basis functions $\phi_{k}\left(t\right)$ (A11) corresponding to exponential functions $h_{k}\left(v\right)$ (A9) of the time-scale factor α=1.97 s and K=20 components, determined for the regularization parameter $\lambda =8.3\times 10^{-8}\;s^{-2}$, and N=100 and N=7500 measurements corrupted by additive zero-mean normal-distribution noises $z\left(t_{i}\right)$ with standard deviation: (**a,b**) σ=0.0005 MPa and (**c**,**d**) σ=0.003 MPa.

Figure 4 presents the courses of the modulus models $G_{K}\left(t,{\bar{g}}_{N,K}^{\lambda }\right)$ (15) for N=100 and N=7500 measurements corrupted by the weakest and the strongest disturbances; measurements $\bar{G}\left(t_{i}\right)$ are marked. Despite the model’s average excellent fit to the real relaxation modulus, measured for N=7500 with a relative approximation error $Q_{Nrel}\left({\bar{g}}_{N,K}^{\lambda }\right)$ (47), equal to 0.19% and 0.40%, for the weakest and strongest disturbances, respectively, the optimal modulus model $G_{K}\left(t,{\bar{g}}_{N,K}^{\lambda }\right)$ does not accurately reproduce the measurements for small times t<0.025 s. These errors are a consequence of the properties of the spectrum model basis functions and the resulting basis functions of the modulus adopted in this example. Changing the class of models can, of course, improve the fit of the modulus model. Generally, however, the selection of appropriate classes of models for a specific type of modeled spectrum is not the subject of this paper, which is primarily focused on the asymptotic properties and noise robustness of the identification algorithm and the family of obtained models, regardless of their class. For N=100, the randomly selected sampling times are such that $t_{i}>0.23\;s$; therefore, the accuracy of the modulus approximation for smaller times is unclear. In the next sampling, or when using a different probability distribution, the sampling times $t_{i}$ will change. However, N=100 measurements are definitely too small to achieve independence of the model from the sampling times, so the simulation experiment was performed here, and also in the next examples, for such a small number of measurements only to illustrate the discussed asymptotic properties.

#### 3.10.2. Noise Robustness

**Figure 5:**
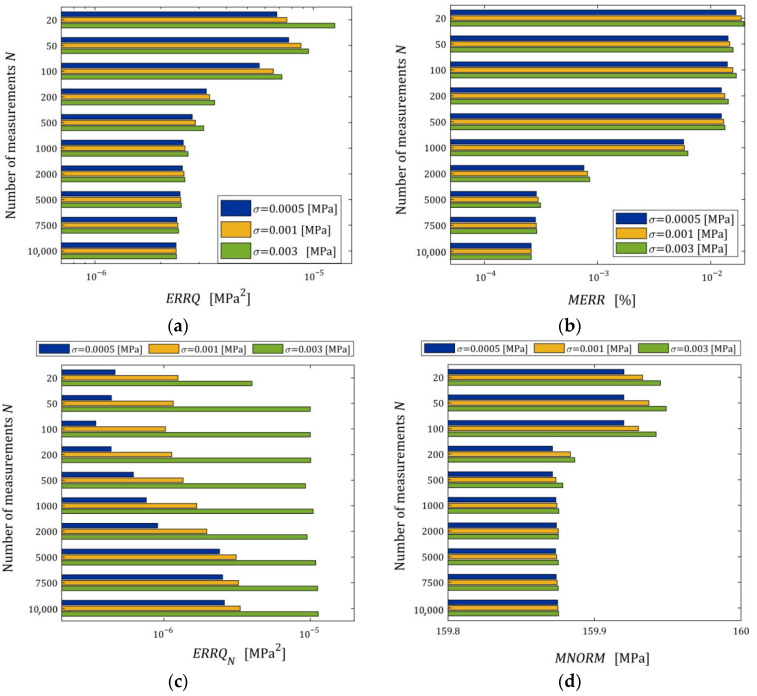
For the KWW relaxation spectra $H\left(\tau \right)$ (54), $H^{M}\left(v\right)$ (55) and series models $H_{K}\left(\tau ,g_{K}\right)$ (9) and $H_{K}^{M}\left(v,g_{K}\right)$ (10), composed of exponential functions $h_{k}\left(\tau \right)$ (A10) and $h_{k}\left(v\right)$ (A9), with time-scale factor α=1.97 s and K=20 model components, the following indices: (**a**) mean integral square error ERRQ (50) of the modulus approximation; (**b**) mean relative percentage error MERR (51) of the sampling-times-independent optimal vector ${\bar{g}}_{K}^{*\lambda }$ (31) approximation; (**c**) mean modulus approximation error $ERRQ_{N}$ (49); and (**d**) mean square norm MNORM (52) of the regularized optimal model parameter ${\bar{g}}_{N,K}^{\lambda }$ (24) as the functions of N for the noises of standard deviations σ=0.0005, 0.001, 0.003 MPa; the regularization parameter $\lambda =8.3\times 10^{-8}\;s^{-2}$.

The impact of disturbances on the relaxation spectra models, studied by n=50 times repetition of the simulated stress relaxation test and the model identification procedure for each $\left(N,\sigma \right)$, is illustrated by the courses of the indices defined above, i.e., $ERRQ_{N}$ (49), ERRQ (50), MERR (51), and MNORM (52), and illustrated in Figure 5 as the functions of N and σ by the bar plots. Logarithmic scales are applied for the index axes.

Index ERRQ (50) decreases with an increasing number of measurements, and increases with the intensity of disturbances, as seen in Figure 5a. These properties are clear in view of the earlier convergence analysis. According to inequality (41), the index $Q\left(g_{K}\right)$, penalized by the regularization summand $\lambda {\left\|g_{K}\right\|}_{2}^{2}$, converges exponentially with the increasing N and with decreasing variance $\sigma^{2}$ of the noises; for details, compare the definition of $\hat{M}$ in Equation (42). For greater N, when $Q\left({\bar{g}}_{N,K}^{\lambda }\right)$ is close to the sampling-times-independent optimal index $Q\left({\bar{g}}_{K}^{*\lambda }\right)$, the influence of $\sigma^{2}$ on $Q\left({\bar{g}}_{N,K}^{\lambda }\right)$ decreases significantly and is negligibly small for N≥2000 (see Figure 5a). This is consistent with the estimate from inequality (41), where the probability estimated by the right-hand side of (41) decreases exponentially to zero and proves the model noise robustness.

The decreasing, with increasing N, influence of noise intensity on the mean relative error MERR (51) and the mean square norm of the regularized optimal model parameter MNORM (52), i.e., on the smoothness of the model parameter ${\bar{g}}_{N,K}^{\lambda }$, also proves the noise robustness. The error MERR does not exceed 0.019%, even for the strongest noises, regardless of the number of measurements, which proves an excellent approximation of the sampling-times-independent parameter ${\bar{g}}_{K}^{*\lambda }$ by the identified parameter ${\bar{g}}_{N,K}^{\lambda }$ even for the strongest noises.

We see that, for the smallest and intermediate noises, the mean modulus approximation error $ERRQ_{N}$ decreases with increasing N only for a very small number of measurements N≤100, for 100<N<5000 increases; for N≥5000 measurements, the index $ERRQ_{N}$ does not depend significantly on N, the further increase in which does not improve the quality of the modulus approximation. From the inspection of Figure 5c, we can also see that, for the strongest noises and N≥50, the empirical index $ERRQ_{N}$ does not depend essentially on the number of measurements varying between $1.137308\times 10^{-5}$ $M{Pa}^{2}$ (for N=1000) and $9.285899\times 10^{-6}$ $M{Pa}^{2}$ (for N=500), with the mean value for N≥50 equal to $1.032176\times 10^{-5}$ $M{Pa}^{2}$. This is not difficult to explain by having in mind Equation (32) and calculating the last term of its right-hand side, i.e., the noise variance $\sigma^{2}$ equal to $2.5\times 10^{-7}$ $M{Pa}^{2}$, $1.0\times 10^{-6}$ $M{Pa}^{2}$, and $9.0\times 10^{-6}$ $M{Pa}^{2}$ for σ=0.0005, 0.001, 0.003 MPa, respectively. Since the optimal $\left({\bar{g}}_{K}^{*\lambda }\right)=2.324634\times 10^{-6}$ ${MPa}^{2}$, the noise variance $\sigma^{2}=9.0\times 10^{-6}\;M{Pa}^{2}$ constitutes, on average, 87.19% of the index $ERRQ_{N}$ value. But even for large noises, the average mean relative error of the real modulus approximation $Q_{Nrel}\left({\bar{g}}_{N,K}^{\lambda }\right)$ (47) does not exceed 0.4% for N≥500, which means that the algorithm ensures very good modulus approximation. For small noises and N≥500, the average index $Q_{Nrel}\left({\bar{g}}_{N,K}^{\lambda }\right)$ does not exceed 0.22%, i.e., an excellent fit to $G\left(t\right)$ (53) is obtained.

### 3.11. Uni-Mode Gauss-like Spectrum

The uni-mode Gauss-like relaxation frequencies distribution [17,18,32,40]

(56) $$H\left(v\right)=\vartheta ve^{-{\left(v-m\right)}^{2}/q},$$

with the parameters [17,18,32] ϑ=31.52 kPa·s, $m=0.0912\;s^{-1}$, and $q=3.25\times 10^{-3}\;s^{-2}$, is considered, which corresponds to

(57) $$H^{M}\left(v\right)=\vartheta e^{-{\left(v-m\right)}^{2}/q}.$$

The ‘real’ modulus [18,32]

(58) $$G\left(t\right)=\frac{\vartheta \sqrt{\pi q}}{2}e^{\frac{1}{4}t^{2}q-mt}erfc\left(\frac{\frac{1}{2}tq-m}{\sqrt{q}}\right)$$

is expressed by the complementary error function [73]

(59) $$erfc\left(x\right)=\frac{2}{\sqrt{\pi \;}\;}\int_{x}^{\infty }e^{{-z}^{2}}dz,$$

and is bounded on $T=\left[0,T\right]$ for any T>0, i.e., Assumption 1 is fulfilled.

Based on the research [16] on the use of various orthogonal bases for uni-mode Gauss-like spectrum modeling, functions $h_{k}\left(v\right)$ (A12) of the spectrum model using Legendre functions were adopted. In view of Property A.3, Assumption 4 holds for $v_{0}=0$, and Assumption 3 is fulfilled for the related functions $\phi_{k}\left(t\right)$ (A15) for t≥0. Following [17,18,32], $T=\left[0,200\right]$ seconds was chosen.

In the preliminary experiment (steps 1 and 2), N=500 equidistant sampling times $t_{i}$ were generated in T. The noises $z\left(t_{i}\right)$ were selected randomly with uniform distribution on the interval $\left[-0.001,\;0.001\right]\;kPa$. The factor α=15 s was selected for K=12 components of the model $G_{K}\left(t,g_{K}\right)$ (15) by comparing, for different values of α, a few first $\phi_{k}\left(t\right)$ with $\bar{G}\left(t_{i}\right)$.

Next, for N=100, the sampling times $t_{1},\ldots ,t_{N}$ were generated from T according to the uniform distribution of density function $\rho \left(t\right)=\frac{1}{200}1_{\left[0,\;200\right]}\left(t\right)$, and a simulated experiment was performed for the material $G\left(t\right)$ (58); normal distribution additive noises of σ=0.001 kPa were applied. Using the GCV rule, the regularization parameter $\lambda =1\times 10^{-6}\;s^{-1}$ (step 5) was determined; next, it was applied together with α=15 s and K=12 for successive simulations. For the noises’ of σ=0.001, 0.005, 0.01 kPa, the experiment was repeated for fourteen values of N, starting from 50 to 25,000. The parameters ${\bar{g}}_{N,K}^{\lambda }$ were determined, and the indices evaluating model quality $Q_{N}\left({\bar{g}}_{N,K}^{\lambda }\right)$ (19), $Q\left({\bar{g}}_{N,K}^{\lambda }\right)$ (29), $Q_{Nrel}\left({\bar{g}}_{N,K}^{\lambda }\right)$ (47), ${\left\|{\bar{g}}_{N,K}^{\lambda }\right\|}_{2}$, and ERR (48) are summarized in Table 2 for the weakest noises, and in Tables S3 and S4 (Supplementary Materials) for other noises.

#### 3.11.1. Asymptotic Properties

**Figure 6:**
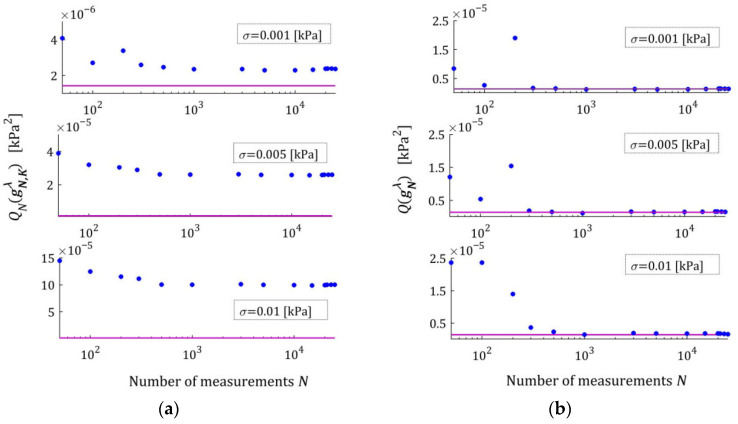
The identification indices (blue dots) of the uni-mode Gauss-like modulus $G\left(t\right)$ (58) approximation by model $G_{K}\left(t,g_{K}\right)$ (15), with basis functions $\phi_{k}\left(t\right)$ (A15), corresponding to the frequency spectrum model using Legendre functions, with time-scale factor α=15 s and K=12 model components, determined for the regularization parameter $\lambda =1\times 10^{-6}\;s^{-1}$: (**a**) mean-square index $Q_{N}\left({\bar{g}}_{N,K}^{\lambda }\right)$ (19) and (**b**) integral approximation index $Q\left({\bar{g}}_{N,K}^{\lambda }\right)$ (29) as the functions of N for σ=0.001, 0.005, 0.01 kPa; the horizontal purple lines marks $Q\left({\bar{g}}_{K}^{*\lambda }\right)$ defined in (30).

Figure 6, where the indices $Q_{N}\left({\bar{g}}_{N,K}^{\lambda }\right)$ (19) and $Q\left({\bar{g}}_{N,K}^{\lambda }\right)$ (29) are depicted for the growing number of measurements N, illustrates their asymptotic convergence for the noises of σ=0.001, 0.005, 0.01 kPa. In any subplot, the value of the optimal sampling-times-independent integral index $Q\left({\bar{g}}_{K}^{*\lambda }\right)=1.426269\times 10^{-6}$ ${kPa}^{2}$ is plotted with horizontal violet lines. We see that, for N≥1000, the fluctuations of the empirical index $Q_{N}\left({\bar{g}}_{N,K}^{\lambda }\right)$ are negligibly small, while $Q\left({\bar{g}}_{N,K}^{\lambda }\right)$ stabilizes already for N≥500 even for the strongest disturbances. Even for large noises, the mean relative error of the real modulus approximation $Q_{Nrel}\left({\bar{g}}_{N,K}^{\lambda }\right)$ (47) does not exceed 0.07% for N≥100, which means that the algorithm guarantees an excellent fit to $G\left(t\right)$ (58).

**Figure 7:**
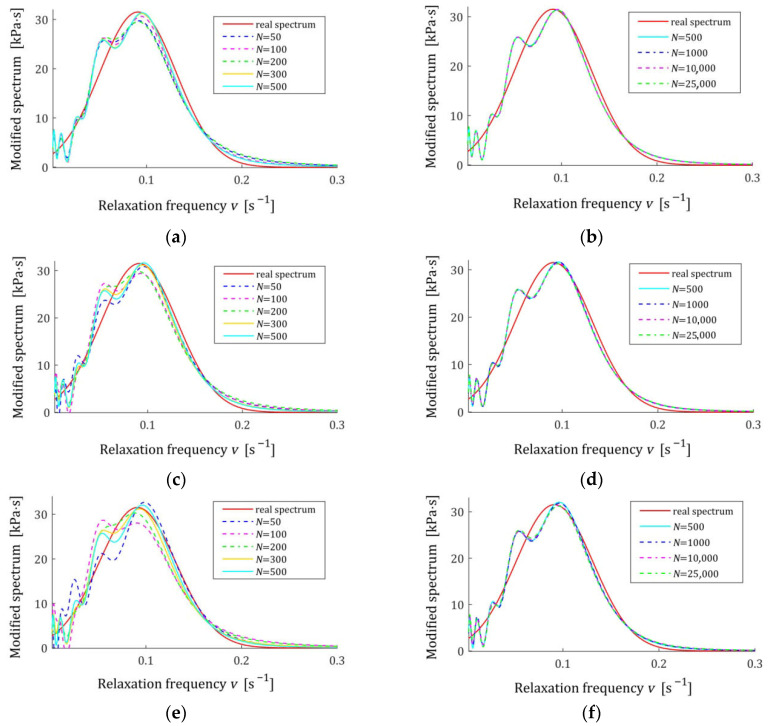
Optimal models $H_{K}^{M}\left(v,{\bar{g}}_{N,K}^{\lambda }\right)$ (10) defined by Legendre basis functions $h_{k}\left(v\right)$ (A12), of time-scale factor α=15 s and K=12 model components of spectrum $H^{M}\left(v\right)$ (57), determined for the regularization parameter $\lambda =1\times 10^{-6}\;s^{-1}$ for selected values of N and noises of standard deviations: (**a**,**b**) σ=0.001 kPa; (**c**,**d**) σ=0.005 kPa; (**e**,**f**) σ=0.01 kPa.

The optimal relaxation frequency models $H_{K}^{M}\left(v,{\bar{g}}_{N,K}^{\lambda }\right)$ (10) with Legendre basis functions $h_{k}\left(v\right)$ (A12) and the modified spectrum $H^{M}\left(v\right)$ (57) are depicted in Figure 7 for some N; the related models $H_{K}^{M}\left(v,{\bar{g}}_{N,K}^{\lambda }\right)v$ of the spectrum $H\left(v\right)$ (56) are plotted in Figure S1 (Supplementary Materials). The optimal models $G_{K}\left(t,{\bar{g}}_{N,K}^{\lambda }\right)$ (15), defined by basis functions $\phi_{k}\left(t\right)$ (A15), are plotted in Figure S2 for selected N; the measurements $\bar{G}\left(t_{i}\right)$ of $G\left(t\right)$ (58) are also marked. Only extreme, the strongest and weakest, noises are included here to limit figure sizes. These plots confirm an excellent measurement data fitting, as indicated by the $Q_{N}\left({\bar{g}}_{N,K}^{\lambda }\right)$ values.

The plots of the spectra models $H_{K}^{M}\left(v,{\bar{g}}_{N,K}^{\lambda }\right)$ in Figure 7 confirm the asymptotic properties described by the variability of $Q_{N}\left({\bar{g}}_{N,K}^{\lambda }\right)$ and $Q\left({\bar{g}}_{N,K}^{\lambda }\right)$. For N≥500, the models $H_{K}^{M}\left(v,{\bar{g}}_{N,K}^{\lambda }\right)$ are also almost visually identical for the strongest noises; negligible small changes in both the empirical and integral model approximation indices confirm it. For the weakest noises σ=0.001 kPa, the modified models $H_{K}^{M}\left(v,{\bar{g}}_{N,K}^{\lambda }\right)$ are almost identical already for N≥400; c.f., Figure 7a. This regularity also applies to the models $H_{K}^{M}\left(v,{\bar{g}}_{N,K}^{\lambda }\right)v$ from Supplementary Figure S1. We see that these models of the spectrum $H\left(v\right)$ (56) are better smoothed than models $H_{K}^{M}\left(v,{\bar{g}}_{N,K}^{\lambda }\right)$ and provide a much better approximation to the real spectrum, which is excellent for N≥500. In summary, in this case, the models $H_{K}^{M}\left(v,{\bar{g}}_{N,K}^{\lambda }\right)$ have a lower quality fit to the real spectrum $H^{M}\left(v\right)$ (57) than the models $H_{K}^{M}\left(v,{\bar{g}}_{N,K}^{\lambda }\right)v$ approximating spectrum $H\left(v\right)$ (56).

The errors ERR (48) of ${\bar{g}}_{K}^{*\lambda }$ and ${\bar{g}}_{N,K}^{\lambda }$ divergence falls below 1% already for N≥300, even for the strongest noises, and does not exceed 0.11% when N≥500, i.e., is much smaller than for the KWW spectrum, with very short relaxation times. This also proves much faster convergence in the case considered here than for the previously considered KWW spectrum.

#### 3.11.2. Noise Robustness

**Figure 8:**
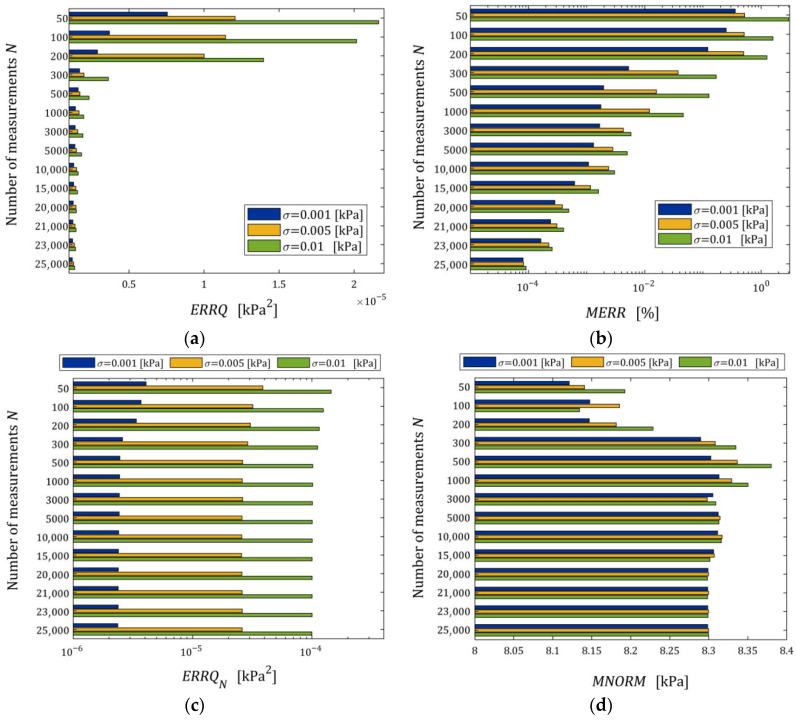
For spectrum $H^{M}\left(v\right)$ (57) and series models $H_{K}^{M}\left(v,g_{K}\right)$ (10) composed of Legendre basis functions $h_{k}\left(v\right)$ (A12),, with the time-scale factor α=15 s and K=12 model components, the indices (**a**) mean integral square error ERRQ (50), (**b**) mean relative error MERR (51) of the sampling-times-independent optimal vector ${\bar{g}}_{K}^{*\lambda }$ (31) approximation, (**c**) mean error $ERRQ_{N}$ (49), and (**d**) mean square norm MNORM (52) of the parameter ${\bar{g}}_{N,K}^{\lambda }$ (24), as the functions of N for disturbances of standard deviations σ=0.001, 0.005, 0.01 kPa; regularization parameter $\lambda =1\times 10^{-6}\;s^{-1}$.

As above, the impact of disturbances on the spectrum $H^{M}\left(v\right)$ (57) models, studied by n=50 times repetition of the simulated experiment and the model identification procedure for each pair $\left(N,\sigma \right)$, is illustrated by the courses of the indices $ERRQ_{N}$ (49), ERRQ (50), MERR (51), and MNORM (52), and illustrated in Figure 8 by the bar plots. Linear scales are used for the MNORM and ERRQ axes, and logarithmic scales for the remaining index axes.

The integral index ERRQ (50) shows a very fast exponential decrease with an increasing number of measurements, as seen in Figure 8a, which confirms the analytical analysis carried out above based on inequality (41). As previously, for greater N≥1000, when $Q\left({\bar{g}}_{N,K}^{\lambda }\right)$ is close to the sampling-times-independent optimal index $Q\left({\bar{g}}_{K}^{*\lambda }\right)=1.426269\times 10^{-6}$ $k{Pa}^{2}$, the effect of the noise variance $\sigma^{2}$ decreases significantly, which proves the noise robustness.

We see that the mean empirical approximation error $ERRQ_{N}$ decreases with an increasing number of measurements both for weak and strong noises; for N≥500, it does not depend significantly on N, i.e., the further increase in N does not improve the quality of the relaxation approximation, which is congruent with the earlier asymptotic property analyses of the models $H_{K}^{M}\left(v,{\bar{g}}_{N,K}^{\lambda }\right)$ (10) and $H_{K}\left(v,{\bar{g}}_{N,K}^{\lambda }\right)=H_{K}^{M}\left(v,{\bar{g}}_{N,K}^{\lambda }\right)v$ (see also Figure 7 and Figure S1). As previously, the explanation follows from Equation (32). Here, the optimal $\left({\bar{g}}_{K}^{*\lambda }\right)=1.426269\times 10^{-6}$ ${kPa}^{2}$. For N≥500, the mean values of index $ERRQ_{N}$ are $2.409703\times 10^{-6}\;k{Pa}^{2}$, $2.585406\times 10^{-5}\;k{Pa}^{2}$, and $1.005519\times 10^{-6}\;k{Pa}^{2}$ for the noises σ=0.001,0.005,0.01kPa, respectively. Therefore, the noise variance $\sigma^{2}$, being equal to 1 $\times \;10^{-6}$ $k{Pa}^{2}$, 2.5$\;\times \;10^{-5}k{Pa}^{2}$, and 1$\;\times \;10^{-4}$ $k{Pa}^{2}$, constitute, on average, 41.49% of the index $ERRQ_{N}$ value for the weak noises, and even 96.7% and 99.45% for the strongest noises (σ=0.005, 0.01 kPa), respectively. For small noises and N≥100, index $ERRQ_{N}$ does not exceed $3.8\times 10^{-6}$ $k{Pa}^{2}$, i.e., an excellent accuracy of the measurement data fit is obtained, as illustrated by Figure S2a,b. Even for large noises and N≥500, index $ERRQ_{N}$ does not exceed $1\times 10^{-4}$ $k{Pa}^{2}$, which also means very good measurement approximation (see Figure S2c,d).

The error MERR (51) exponentially depends on the increasing N (logarithmic MERR scale); the effect of noise intensity also decreases with increasing N, which confirms the good noise robustness. For N≥100, the errors MERR do not exceed 0.25% and 0.51% for the weakest and intermediate disturbances, and 1.59% for the strongest noises, which proves an excellent approximation of the sampling-times-independent model parameter ${\bar{g}}_{K}^{*\lambda }$ by the identified parameter ${\bar{g}}_{N,K}^{\lambda }$.

The decreasing influence, when the number of measurements increases, of the noise intensity on the parameter ${\bar{g}}_{N,K}^{\lambda }$ smoothing, whose measure is the mean square norm MNORM (52), also proves the noise robustness.

### 3.12. Double-Mode Gauss-like Spectrum

Let us consider the double-mode Gauss-like spectrum described by [17,18,32,40,64]:

(60) $$H\left(\tau \right)=\left[\vartheta_{1}e^{-{\left(\frac{1}{\tau }-m_{1}\right)}^{2}/q_{1}}+\vartheta_{2}e^{-{\left(\frac{1}{\tau }-m_{2}\right)}^{2}/q_{2}}\right]/\tau ,$$

where the following coefficients [17,32,40]: $\vartheta_{1}=467\;Pa\cdot s$, $\vartheta_{2}=39\;Pa\cdot s$, $m_{1}=0.0037\;s^{-1}$, $m_{2}=0.045\;s^{-1}$, $q_{1}=1.124261\times 10^{-6}\;s^{-2}$, and $q_{2}=1.173\times 10^{-3}\;s^{-2}$ are motivated by polyethylene data from [64] (Table 1; Figure 8b). With (58), the related real modulus is described by

(61) $$G\left(t\right)=\frac{\sqrt{\pi }}{2}\left[\vartheta_{1}\sqrt{q_{1}}e^{\frac{1}{4}t^{2}q_{1}-m_{1}t}erfc\left(\frac{\frac{1}{2}tq_{1}-m_{1}}{\sqrt{q_{1}}}\right)+\vartheta_{2}\sqrt{q_{2}}e^{\frac{1}{4}t^{2}q_{2}-m_{2}t}erfc\left(\frac{\frac{1}{2}tq_{2}-m_{2}}{\sqrt{q_{2}}}\right)\right],$$

and, like uni-mode Gauss-like material, satisfies Assumption 1 for $T=\left[0,T\right]$, where an arbitrary T>0.

Taking into account the results of [17], the relaxation time spectrum (60) was modeled by a series of power-exponential basis functions, $h_{k}\left(\tau \right)$ (A1), and (A2), resulting in the modulus basis functions $\phi_{k}\left(t\right)$ (A3), and (A4) using Bessel functions. By Property A.1, Assumption 4 holds for functions $h_{k}\left(\tau \right)$ whenever $\tau_{0}=0$, while Assumption 3 is fulfilled by $\phi_{k}\left(t\right)$ (A3), and (A4), forming the modulus models $G_{K}\left(t,g_{K}\right)$ for an arbitrary t>0.

Following [17,40,53], the interval $T=\left[1,1551\right]$ seconds was taken for numerical simulations in view of the singularities of the basic functions $\phi_{k}\left(t\right)$ (A3) at t=0, i.e., $t_{0}=1\;s$. Therefore, the weighting function is $\rho \left(t\right)=\frac{1}{1550}1_{\left[1,\;1550\right]}\left(t\right)s^{-1}$.

Based on the experiments from [17] and a preliminary test performed for N=5000 equidistant sampling times from T, with the modulus measurements corrupted by the additive $z\left(t_{i}\right)$ selected independently with a zero-mean normal distribution of σ=0.002 Pa, the model of K=12 components was assumed with the factor $\alpha =0.04\;s^{-1}$. Next, for N=100, the sampling instants $t_{1},\ldots ,t_{N}$ were generated from the set T according to the uniform distribution of the assumed density function $\rho \left(t\right)=\frac{1}{1550}1_{\left[1,\;1550\right]}\left(t\right)$, and a simulated stress relaxation experiment was performed for $G\left(t\right)$ (61) and the additive independent noises of the zero-mean normal distribution with standard deviation σ=0.002 Pa. By applying the GCV rule, the dimensionless parameter $\lambda =3\times 10^{-6}$ was chosen, being applied together with $\alpha =0.04\;s^{-1}$ and K=12, for successive simulations.

Assuming standard deviations σ=0.002, 0.005, 0.01 Pa of the measurement noises, the experiment was repeated for ten values of N, starting from 100 to 25,000. The optimal parameters ${\bar{g}}_{N,K}^{\lambda }$ were determined and the indices evaluating model quality, $Q_{N}\left({\bar{g}}_{N,K}^{\lambda }\right)$ (19), $Q\left({\bar{g}}_{N,K}^{\lambda }\right)$ (29), $Q_{Nrel}\left({\bar{g}}_{N,K}^{\lambda }\right)$ (47), ${\left\|{\bar{g}}_{N,K}^{\lambda }\right\|}_{2}$, and ERR (48), were computed. They are given in Table 3 for the weakest noises, and in Tables S5 and S6 for the intermediate and strong noises.

#### 3.12.1. Asymptotic Properties

**Figure 9:**
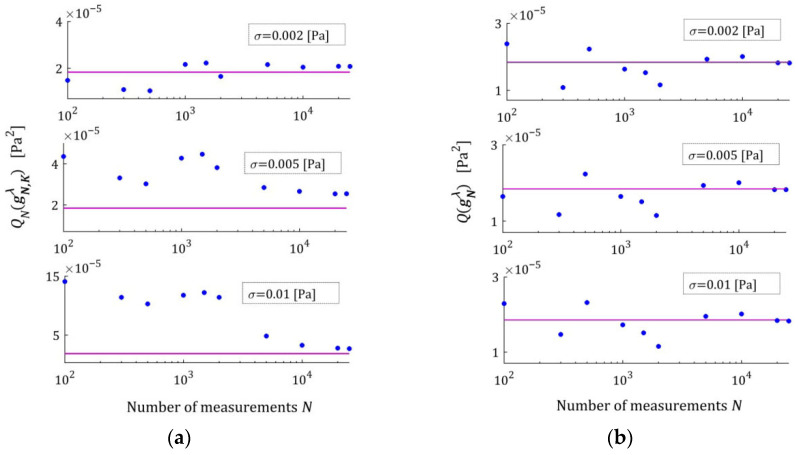
The identification indices (blue dots) of the double-mode Gauss-like modulus $G\left(t\right)$ (61) approximation by model $G_{K}\left(t,g_{K}\right)$ (15) with the basis functions $\phi_{k}\left(t\right)$ (A3) and (A4), using Bessel functions of the time-scale parameter $\alpha =0.04\;s^{-1}$ and K=12 model components, determined with the regularization parameter $\lambda =3\times 10^{-6}$: (**a**) mean-square identification index $Q_{N}\left({\bar{g}}_{N,K}^{\lambda }\right)$ (19) and (**b**) integral approximation index $Q\left({\bar{g}}_{N,K}^{\lambda }\right)$ (29) as the functions of N for disturbances of σ=0.002, 0.005, 0.01 Pa; the horizontal purple lines mark $Q\left({\bar{g}}_{K}^{*\lambda }\right)$, as defined in (30).

The indices $Q_{N}\left({\bar{g}}_{N,K}^{\lambda }\right)$ (19) and $Q\left({\bar{g}}_{N,K}^{\lambda }\right)$ (29), as the functions of N, are illustrated in Figure 9; the value of the optimal sampling-times-independent index is plotted with horizontal violet lines, where $Q\left({\bar{g}}_{K}^{*\lambda }\right)=1.772535\times 10^{-5}$ ${Pa}^{2}$.

**Figure 10:**
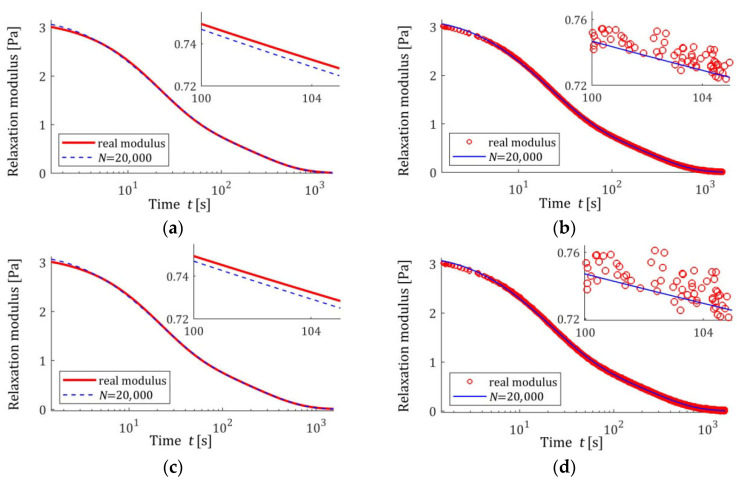
Optimal models $G_{K}\left(t,{\bar{g}}_{N,K}^{\lambda }\right)$ (15) with basis functions $\phi_{k}\left(t\right)$ (A3) and (A4), using Bessel functions of the time-scale parameter $\alpha =0.04\;s^{-1}$ and K=12 model components, determined with the regularization parameter $\lambda =3\times 10^{-6}$, approximating for N= 20,000 the measurements $\bar{G}\left(t_{i}\right)$ (red points) of the real relaxation modulus $G\left(t\right)$ (61) for noises of standard deviations: (**a**,**b**) σ=0.005 Pa; (**c**,**d**) σ=0.01 Pa.

We see that the integral index $Q\left({\bar{g}}_{N,K}^{\lambda }\right)$ stabilizes for N≥5000 even for the strongest disturbances; the fluctuations of the empirical index $Q_{N}\left({\bar{g}}_{N,K}^{\lambda }\right)$ for the weak and intermediate disturbances stabilize for N≥5000; for the strongest noises, only for N≥20,000. The relative error $Q_{Nrel}\left({\bar{g}}_{N,K}^{\lambda }\right)$ is much larger here than in the two previous examples, regardless of N and the power of the noises. Index $Q_{Nrel}\left({\bar{g}}_{N,K}^{\lambda }\right)$ changes from 1.306% to 1.79%; its average value is 1.62%, which means a significantly worse real modulus approximation than in the previous examples. For N=20,000 measurements, and the intermediate and strong noises, the measurements data fitting by models $G_{K}\left(t,{\bar{g}}_{N,K}^{\lambda }\right)$ (15) is illustrated by Figure 10b,d, while Figure 10a,c illustrate the real modulus $G\left(t\right)$ (61) approximation. The same characteristics for only N=100 measurements are illustrated in Figure S3. An insightful analysis of Figure 10a,c shows discrepancies between $G_{K}\left(t,{\bar{g}}_{N,K}^{\lambda }\right)$ and the real relaxation modulus $G\left(t\right)$; compare both characteristics for small times t≤2.5 s and small subfigures. The fit of the models to the measurements $\bar{G}\left(t_{i}\right)$, illustrated in Figure 10b,d, shows similar discrepancies. For N=20,000, these discrepancies do not depend on the noise intensity; compare especially the small subfigures, which indicate the noise robustness of the identification scheme. The significantly different discrepancies between the model and the real relaxation modulus for σ=0.005 Pa and σ=0.01 Pa, illustrated by Figure S3a,c, are a consequence of too few measurement points N=100, even though the relative fit indices $Q_{Nrel}\left({\bar{g}}_{N,K}^{\lambda }\right)$ are noticeably smaller here than for N=20,000.

**Figure 11:**
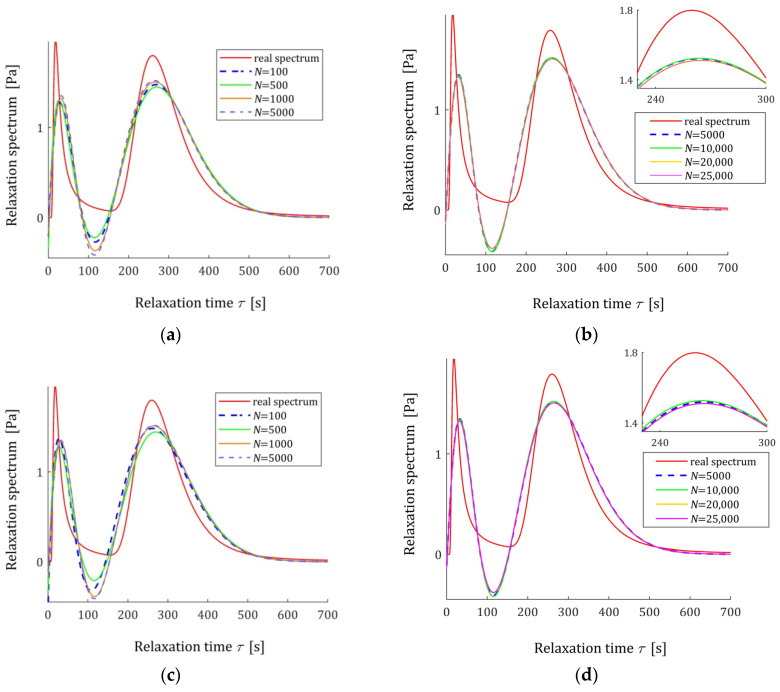
Double-mode Gauss-like spectrum $H\left(\tau \right)$ (60) and the model $H_{K}\left(\tau ,{\bar{g}}_{N,K}^{\lambda }\right)$ (9), with power exponential basis functions $h_{k}\left(\tau \right)$ (A1) and (A2) of the time-scale coefficient $\alpha =0.04\;s^{-1}$ and K=12 model components for selected values of the number of measurements N and noises of standard deviations: (**a**,**b**) σ=0.002 Pa; (**c**,**d**) σ=0.01 Pa; regularization parameter $\lambda =3\times 10^{-6}$.

Selected values of N spectra models $H_{K}\left(\tau ,{\bar{g}}_{N,K}^{\lambda }\right)$ (9) are plotted in Figure 11 for the weakest and strongest noises, and in Figure S4 for the intermediate noise. For successive N≥5000, the optimal models $H_{K}\left(\tau ,{\bar{g}}_{N,K}^{\lambda }\right)$ practically coincide with each other; therefore, further increasing (above 5000) the number of measurements does not noticeably change the relaxation spectra models. See especially the small subplots in Figure 11b,d, as well as Figure S4b, illustrating the real spectrum and models in the near neighborhood of the second maximum, where only for the strongest noises can small differences between the models be noticed.

#### 3.12.2. Noise Robustness

**Figure 12:**
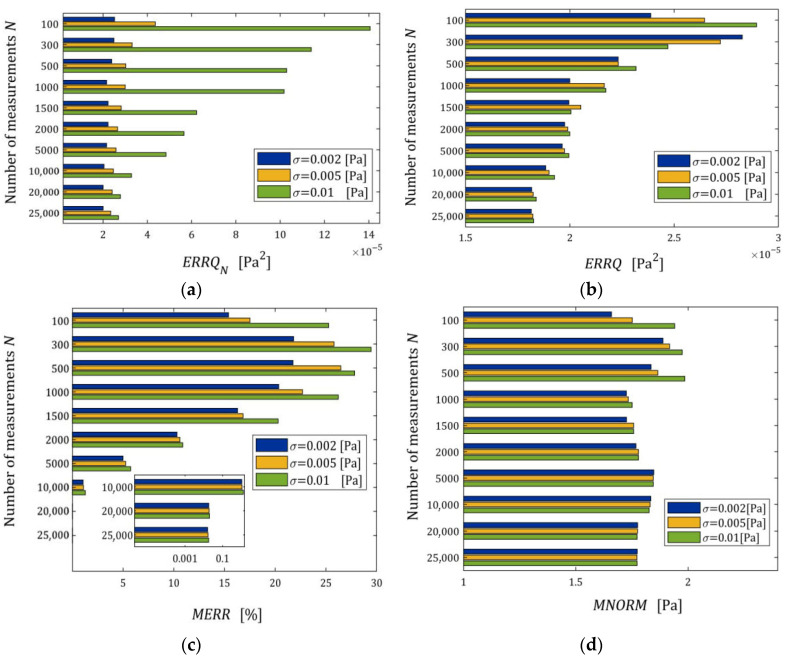
For the double-mode Gauss-like spectrum $H\left(\tau \right)$ (60) and the model $H_{K}\left(\tau ,{\bar{g}}_{N,K}^{\lambda }\right)$ (9), with the power-exponential basis functions $h_{k}\left(\tau \right)$ (A1) and (A2) of the time-scale coefficient $\alpha =0.04\;s^{-1}$ and K=12 model components, determined with the regularization parameter $\lambda =3\times 10^{-6}$, the model quality indices (**a**) $ERRQ_{N}$ (49), (**b**) ERRQ (50), (**c**) mean relative percentage error MERR (51) of the sampling-times-independent optimal vector ${\bar{g}}_{K}^{*\lambda }$ (31) approximation, and (**d**) mean square norm MNORM (52) of ${\bar{g}}_{N,K}^{\lambda }$ (24) as the functions of N for noises of standard deviations σ.

The indices $ERRQ_{N}$ (49), ERRQ (50), MERR (51), and MNORM (52), evaluating the impact of the measurement noises on the relaxation spectrum $H\left(\tau \right)$ (60) models, as examined by the n=50 times repetition of the simulated experiment and the identification procedure for any pair $\left(N,\sigma \right)$, are illustrated in Figure 12.

The integral index ERRQ (50) exponentially decreases with an increasing number of measurements, as seen in Figure 12b. For N≥5000, the relative square error between ERRQ (50) and the optimal sampling-times-independent integral index $Q\left({\bar{g}}_{K}^{*\lambda }\right)=1.772535\times 10^{-5}$ ${Pa}^{2}$, defined as

(62) $$RERRQ=\frac{{\left[ERRQ-Q\left({\bar{g}}_{K}^{*\lambda }\right)\right]}^{2}}{{\left[Q\left({\bar{g}}_{K}^{*\lambda }\right)\right]}^{2}},$$

do not exceed 1.6% even for the strongest noises, while, for N≥10,000, the RERRQ error drops below 0.75%. When ERRQ is so close to the index $Q\left({\bar{g}}_{K}^{*\lambda }\right)$, the effect of the noise variance $\sigma^{2}$ decreases significantly (see Figure 12b), which proves the noise robustness.

From the quick inspection of Figure 12a, we see that the mean empirical approximation error $ERRQ_{N}$ monotonically decreases with growing N for the noises; for weak and intermediate noises, it does not depend significantly on the number of measurements for N≥2000; for the strongest noises, only for N≥10,000, the further increase in N does not improve the quality of the relaxation modulus approximation. However, the earlier asymptotic analysis of the model $H_{K}\left(\tau ,{\bar{g}}_{N,K}^{\lambda }\right)$ (9) combined with the small values of $ERRQ_{N}$, which does not exceed $5.0\times 10^{-5}{Pa}^{2}$ already for N≥5000, even for the strongest noises, allows us to assume that N≥5000 measurements are enough for a satisfactory approximation of $H_{K}\left(\tau ,{\bar{g}}_{K}^{*\lambda }\right)$. For N=5000, the mean relative error MERR (51) of ${\bar{g}}_{K}^{*\lambda }$ approximation does not exceed 5.75% even for the strongest noises. However, for N≥10,000, the error MERR, which, for N≥500, exponentially decreases with the increasing number of measurements (see Figure 12c), does not exceed 1.28%, falling below 0.02% for N≥20,000, which proves an excellent approximation of ${\bar{g}}_{K}^{*\lambda }$ by ${\bar{g}}_{N,K}^{\lambda }$. Simultaneously, for N≥20,000, the effect of the measurement noise intensity on the error MERR noticeably decreases, confirming the noise robustness. The decreasing influence, with the increasing number of measurements, of noise intensity on the model parameter ${\bar{g}}_{N,K}^{\lambda }$ smoothness (i.e., on the mean square norm MNORM), already for N≥1000, also proves the noise robustness.

### 3.13. The Baumgaertel, Schausberger, and Winter Relaxation Spectrum

In this example, we consider the identification of the BSW modified spectrum [16,21,65]:

(63) $$H^{M}\left(v\right)=\frac{1}{v}\left\{\beta_{1}{\left(\frac{1}{v\tau_{c}}\right)}^{\rho_{1}}+\beta_{2}{\left(\frac{1}{v\tau_{c}}\right)}^{\rho_{2}}\right\}e^{-\frac{1}{{v\tau }_{max}}}$$

with the coefficients [16,27] $\tau_{c}=2.481\;s$, $\tau_{max}=2.564\times 10^{4}\;s$, $\beta_{1}=6.276\times 10^{-2}\;MPa$, $\rho_{1}=0.25$, $\beta_{2}=0.127\;MPa$, and $\rho_{2}=-0.5$. With (5), $H^{M}\left(v\right)$ (63) uniquely defines the BSW modulus $G\left(t\right)$. In view of the singularity of the BSW relaxation modulus at t=0, it is assumed that $t_{0}>0$ guarantees its boundness, i.e., the fulfilment of Assumption 1. Following previous studies [16], $T=\left[1,10^{7}+1\right]$ seconds was assumed; thus, the weighting function was $\rho \left(t\right)=\frac{1}{10^{7}}1_{\left[1,10^{7}+1\right]}\left(t\right)s^{-1}$.

In [16], identification of the BSW spectrum (63) was performed using four different orthonormal bases, among which the Laguerre basis provides the best model quality, cf. [16], Table 2, and Figures 7 and 8. Therefore, the Laguerre model, using $h_{k}\left(v\right)$ (A20) and $\phi_{k}\left(t\right)$ (A22), was adopted for spectrum $H^{M}\left(v\right)$ modeling. In view of Property A.4, Assumption 3 is satisfied for $\phi_{k}\left(t\right)$ (A22) and t≥0; Assumption 4 holds for $h_{k}\left(v\right)$ and v≥0.

Based on the results presented in [16] and the preliminary experiment performed for N=500 measurements obtained for the equidistant sampling times, the time-scale factor α=8000 s and K=14 model components were selected. For N=100 relaxation modulus measurements corrupted by noises of σ=0.0005 MPa, the regularization parameter $\lambda =3.2\cdot 10^{-11}\;s^{-1}$ was selected, which was maintained in further calculations.

#### 3.13.1. Asymptotic Properties

A uniform distribution on T was used to select sampling times $t_{i}$. Disturbances $z\left(t_{i}\right)$ were selected randomly with the normal zero-mean distribution of the standard deviation  σ=0.0005, 0.001, 0.003 MPa. For every pair $\left(N,\sigma \right)$, where N varies from 100 to 25,000 measurements, parameters ${\bar{g}}_{N,K}^{\lambda }$ were determined, and the indices $Q_{N}\left({\bar{g}}_{N,K}^{\lambda }\right)$ (19), $Q\left({\bar{g}}_{N,K}^{\lambda }\right)$ (29), $Q_{Nrel}\left({\bar{g}}_{N,K}^{\lambda }\right)$ (47), ${\left\|{\bar{g}}_{N,K}^{\lambda }\right\|}_{2}$, and ERR (48) are summarized in Table 4 for the weakest noises, and in Tables S6 and S7 for intermediate and strong noises.

**Figure 13:**
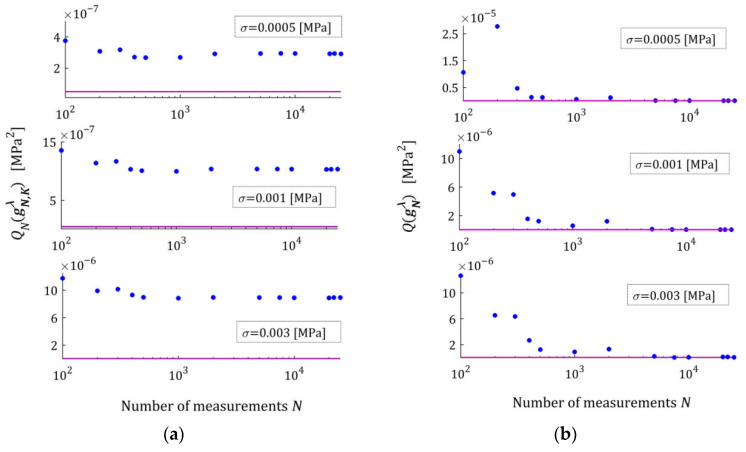
For the BSW modulus $G\left(t\right)$ related to $H^{M}\left(v\right)$ (63), approximated by the optimal model $G_{K}\left(t,g_{K}\right)$ (15) with basis functions $\phi_{k}\left(t\right)$ (A22), corresponding to Laguerre functions $h_{k}\left(v\right)$ (A20), of the time-scale factor α=8000 s and K=14 model components, determined with the regularization parameter $\lambda =3.2\times 10^{-11}\;s^{-1}$, are the following indices (blue dots): (**a**) mean-square identification index $Q_{N}\left({\bar{g}}_{N,K}^{\lambda }\right)$ (19); (**b**) integral index $Q\left({\bar{g}}_{N,K}^{\lambda }\right)$ (29) for different number of measurements N and noises σ=0.0005, 0.001, 0.003 MPa; the horizontal violet lines correspond to the optimal integral index $Q\left({\bar{g}}_{K}^{*\lambda }\right)$ defined in Equation (30).

Figure 13 illustrates, for varying N, the indices $Q_{N}\left({\bar{g}}_{N,K}^{\lambda }\right)$ (19) and $Q\left({\bar{g}}_{N,K}^{\lambda }\right)$ (29), confirming the proved asymptotic properties; in any subplot, the value of $Q\left({\bar{g}}_{K}^{*\lambda }\right)=4.889951\times 10^{-8}$ ${MPa}^{2}$ is plotted with horizontal purple lines. Even for the strongest disturbances, the integral index $Q\left({\bar{g}}_{N,K}^{\lambda }\right)$ stabilizes for N≥5000, while the fluctuations of the empirical mean-square index $Q_{N}\left({\bar{g}}_{N,K}^{\lambda }\right)$ are negligibly small for N≥2000. The mean relative errors of the real modulus approximation $Q_{Nrel}\left({\bar{g}}_{N,K}^{\lambda }\right)$ (47) do not exceed 1% for N≥2000 regardless of the disturbance intensity.

**Figure 14:**
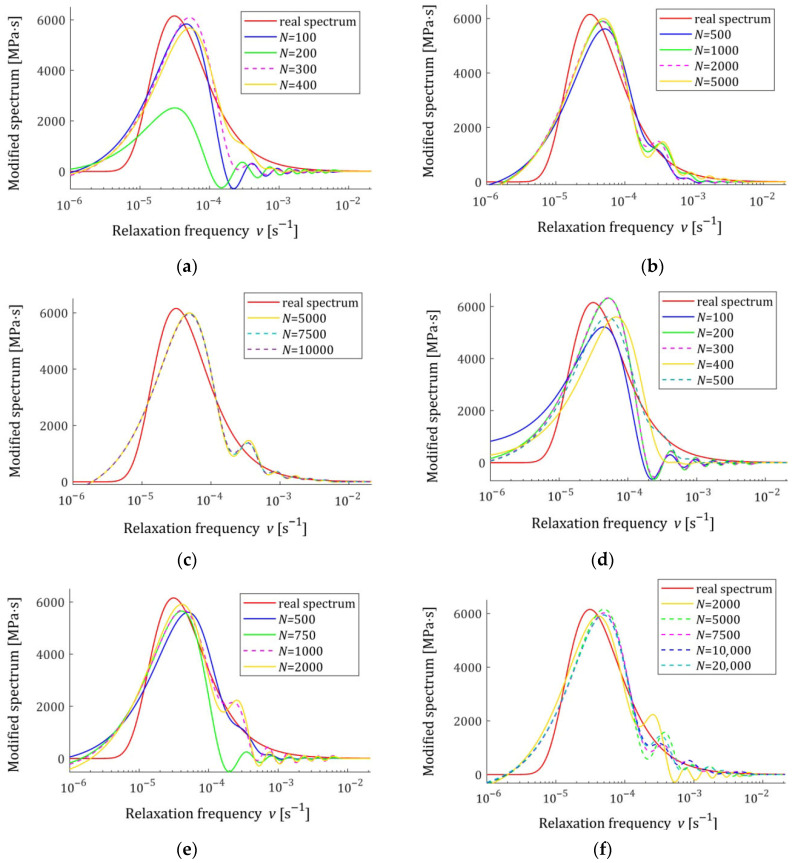
Optimal models $H_{K}^{M}\left(v,{\bar{g}}_{N,K}^{\lambda }\right)$ (10) defined by Laguerre basis functions $h_{k}\left(v\right)$ (A20) of the time-scale factor α=8000 s and K=14 model components and the BSW spectrum $H^{M}\left(v\right)$ (63) for selected numbers of measurements N and noises of standard deviations: (**a**–**c**) σ=0.0005 MPa; (**d**–**f**) σ=0.003 MPa; regularization parameter $\lambda =3.2\times 10^{-11}\;s^{-1}$.

For the weakest and strongest disturbances, the optimal models $H_{K}^{M}\left(v,{\bar{g}}_{N,K}^{\lambda }\right)$ (10) given by Laguerre basis functions $h_{k}\left(v\right)$ (A20) along with the BSW spectrum $H^{M}\left(v\right)$ (63) are depicted in Figure 14 for selected N. The same characteristics for the intermediate noises are plotted in Figure S5. For the weak and intermediate noises, these plots confirm the asymptotic properties described by variability of $Q_{N}\left({\bar{g}}_{N,K}^{\lambda }\right)$ and $Q\left({\bar{g}}_{N,K}^{\lambda }\right)$. For N≥5000, the models $H_{K}^{M}\left(v,{\bar{g}}_{N,K}^{\lambda }\right)$ are visually almost identical, see Figure 14c and Figure S5c. For the strongest noises, σ=0.003 MPa and N≥5000, the optimal models $H_{K}^{M}\left(v,{\bar{g}}_{N,K}^{\lambda }\right)$ are close; they are almost identical already for N≥10,000 (c.f., Figure 14f).

The optimal models $G_{K}\left(t,{\bar{g}}_{N,K}^{\lambda }\right)$ (15) based on the functions $\phi_{k}\left(t\right)$ (A22) of the BSW modulus are plotted in Figure S6 for the weakest and strongest disturbances for two values of N. These plots confirm excellent measurement data fitting, indicated by the values of $Q_{N}\left({\bar{g}}_{N,K}^{\lambda }\right)$, which not exceed 9.0 × 10^−6^ for N≥500 regardless of the power of the noises.

The errors ERR (48) of ${\bar{g}}_{K}^{*\lambda }$ and ${\bar{g}}_{N,K}^{\lambda }$ discrepancy for N≥5000, and the weak and medium disturbances, do not exceed 0.66%; for stronger disturbances, they grow and reach 2.3%, which is reflected in the noticeable differentiation of the models $H_{K}^{M}\left(v,{\bar{g}}_{N,K}^{\lambda }\right)$ even for N≥5000; compare Figure 14c,f.

#### 3.13.2. Noise Robustness

**Figure 15:**
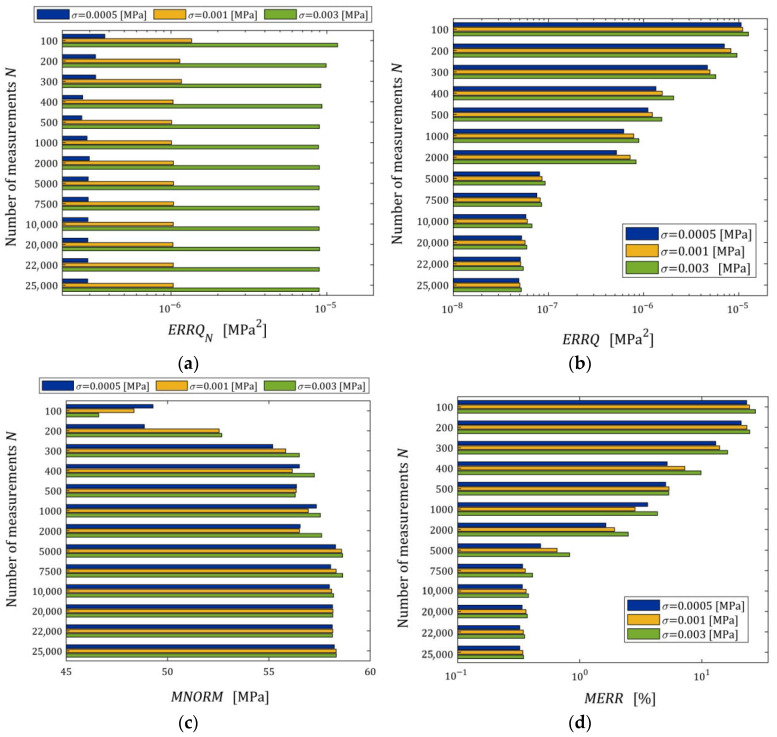
For the BSW spectrum $H^{M}\left(v\right)$ (63), approximated by models $H_{K}^{M}\left(v,{\bar{g}}_{N,K}^{\lambda }\right)$ (10), defined by Laguerre functions $h_{k}\left(v\right)$ (A20) with time-scale factor α=8000 s and K=14 model components, are the indices: (**a**) mean modulus approximation error $ERRQ_{N}$ (49); (**b**) mean integral square error ERRQ (50) of the relaxation modulus approximation; (**c**) mean square norm MNORM (52) of parameter ${\bar{g}}_{N,K}^{\lambda }$ (24), and (**d**) mean relative error MERR (51) of the sampling-times-independent optimal vector ${\bar{g}}_{K}^{*\lambda }$ (31) approximation as functions of N for the noises of standard deviations σ; regularization parameter $\lambda =3.2\times 10^{-11}\;s^{-1}$.

Four indices, $ERRQ_{N}$ (49), ERRQ (50), MERR (51), and MNORM (52), are illustrated in Figure 15.

The integral index ERRQ (50) exponentially decreases with an increasing number of measurements. The relative square error between ERRQ and the optimal sampling-times-independent integral index $Q\left({\bar{g}}_{K}^{*\lambda }\right)=4.889951\times 10^{-8}$ ${MPa}^{2}$, defined by the formula RERRQ (62) for N≥10,000, do not exceed 3.5% and 5.3% for the weak and intermediate noises, respectively; for the strongest noises, they fall below 4.2% only for N≥20,000. For the weak disturbances, the error RERRQ does not exceed 0.52% for N≥20,000, which means excellent model $H_{K}^{M}\left(v,{\bar{g}}_{K}^{*\lambda }\right)$ approximation. For N≥5000, the effect of the noise variance $\sigma^{2}$ decreases significantly for all disturbances (c.f., Figure 15b), which proves the noise robustness.

From the inspection of Figure 15a, we see that the mean empirical error $ERRQ_{N}$ stabilizes for N≥2000 with an increasing N for all noises; with the further increase in N, the quality of the modulus approximation does not improve. However, the error MERR (51) of ${\bar{g}}_{K}^{*\lambda }$ approximation stabilizes only for N≥10,000 for weak and intermediate noises, and for N≥20,000 when the noises are the strongest. Generally, for N≥5000, MERR does not exceed 0.83%, and for N≥10,000, index MERR≤0.4% even for the strongest noises, when the effect of the measurement noise intensity on MERR noticeably decreases, thus confirming the noise robustness. Also, the mean square norm MNORM stabilizes for N≥10,000; the reduced influence of the noise intensity on the model parameter ${\bar{g}}_{N,K}^{\lambda }$ smoothness proves the noise robustness. In summary, for N≥10,000, the optimal model $H_{K}^{M}\left(v,{\bar{g}}_{N,K}^{\lambda }\right)$ is nearly independent of the sampling times for the weak and intermediate noises; however, N≥20,000 measurements are needed to obtain satisfactory approximation of the sampling-times-independent model $H_{K}^{M}\left(v,{\bar{g}}_{K}^{*\lambda }\right)$ for the strongest noises.

## 4. Conclusions

Optimal models of relaxation time and frequency spectra, asymptotically independent of sampling times chosen randomly according to the appropriate randomization used in the experiment, were constructed. The unknown spectra are modeled by a finite series of bounded basis functions and evaluated using the square index related to the relaxation modulus measurements. Therefore, the original inverse ill-posed problem of the continuous spectrum identification is reduced to simple linear-quadratic static minimization task, which is well-posed due to the application of Tikhonov regularization.

The only requirements of the identification scheme applicability are a priori independent random selection of the sampling times from the assumed set according to a stationary rule and the boundness of the basis functions. These conditions are related primarily to the designed experiment and spectrum model, not to the real spectrum; thus, the resulting identification scheme is simple and useful in application. Both analytical analysis and comprehensive numerical simulations performed for real relaxation spectra of a very wide range of relaxation frequencies/times, and four different types of relaxation processes, showed fast convergence and excellent noise robustness.

The identified spectra models are a strongly consistent estimate of the models, corresponding to the relaxation modulus models being optimal in the sense of the deterministic integral weighted square error, since the square empirical identification index is related to the relaxation modulus, which is accessible by experiment. The convergence results, obtained for the relaxation modulus models, can be transferred to relaxation spectra models due to their linear form with respect to the parameters.

In the numerical studies, a uniform probability distribution was used for the random selection of sampling times, which means that the model error was given the same weight over the entire time range. The subject of future research will be to investigate the asymptotic properties of the modulus and relaxation spectra models, and their noise robustness under different probability distributions applied to the random choosing of the sampling times. Of particular interest is the use of an exponential probability distribution, which means that the weight we assign to the model error when defining the sampling-times-independent integral index decreases with a decrease in the falling speed of the relaxation modulus.

The relaxation spectra of real materials are a non-negative definite; the problem of spectra identification, taking into account this natural requirement, was formulated and solved in [40]. The extension of the concept of sampling-times-independent relaxation spectra identification for the task of identifying non-negative definite spectra will be the subject of further research.

Recently, a new approach of relaxation spectrum direct approximation was proposed in [32], where, as a measure of the model accuracy, the integral quadratic error between the real, completely unknown spectrum and its model was taken. The modified relaxation frequency spectrum was approximated by a series of exponential functions. The subject of future research will be to combine the idea of sampling-times-independent identification with the spectra direct identification.

However, in [74], the concept of sampling-times-independent identification was successfully applied for the optimal determination of fractional Maxwell model parameters; its applicability to other fractional-order model identification remains an open question. Another interesting challenge is how to transfer this concept to the area of nonlinear viscoelastic model identification.

## Figures and Tables

**Table 1:** The results of simulations for KWW spectrum $H^{M}\left(v\right)$ (55) and model $H_{K}^{M}\left(v,{\bar{g}}_{N,K}^{\lambda }\right)$ (10) with exponential basis functions $h_{k}\left(v\right)$ (A9), with time-scale factor α=1.97 s and K=20 model components determined for the regularization parameter $\lambda =8.3\times 10^{-8}\;s^{-2}$: empirical $Q_{N}\left({\bar{g}}_{N,K}^{\lambda }\right)$ (19), integral approximation index $Q\left({\bar{g}}_{N,K}^{\lambda }\right)$ (29), relative error of the modulus approximation $Q_{Nrel}\left({\bar{g}}_{N,K}^{\lambda }\right)$ (47), norm of the optimal model parameter ${\left\|{\bar{g}}_{N,K}^{\lambda }\right\|}_{2}$, and relative percentage square error ERR (48) of the sampling-times-independent model parameter ${\bar{g}}_{K}^{*\lambda }$ approximation for N modulus measurements corrupted by zero-mean normal distribution noises of standard deviation σ=0.0005 MPa.

N	$Q_{N}$(${\bar{g}}_{N,K}^{\lambda }$) [MPa^2^]	Q(${\bar{g}}_{N,K}^{\lambda }$) [MPa^2^]	$Q_{Nrel}$(${\bar{g}}_{N,K}^{\lambda }$) [%]	${\left\|{\bar{g}}_{N,K}^{\lambda }\right\|}_{2}$ [MPa·s]	ERR [%]
20	4.629072 × 10^−7^	5.811239 × 10^−6^	2.75751	1.599201 × 10^2^	1.67420 × 10^−2^
50	4.476169 × 10^−7^	8.712334 × 10^−6^	0.84818	1.599199 × 10^2^	1.42023 × 10^−2^
100	3.343028 × 10^−7^	7.655098 × 10^−6^	1.60721	1.599199 × 10^2^	1.40089 × 10^−2^
200	4.064603 × 10^−7^	3.237333 × 10^−6^	0.23437	1.598713 × 10^2^	1.24116 × 10^−2^
500	6.185628 × 10^−7^	2.789343 × 10^−6^	0.19354	1.598713 × 10^2^	1.24116 × 10^−2^
1000	1.587661 × 10^−6^	2.537891 × 10^−6^	0.35183	1.598736 × 10^2^	5.75952 × 10^−3^
2000	9.058092 × 10^−7^	2.712719 × 10^−6^	0.16589	1.598740 × 10^2^	4.56557 × 10^−4^
5000	2.398905 × 10^−6^	2.453493 × 10^−6^	0.20772	1.598734 × 10^2^	2.87684 × 10^−4^
7500	2.721371 × 10^−6^	2.569209 × 10^−6^	0.19446	1.598828 × 10^2^	2.82558 × 10^−4^
10,000	2.622259 × 10^−6^	2.418777 × 10^−6^	0.23976	1.598833 × 10^2^	2.78423 × 10^−4^

**Table 2:** The results of simulations for the uni-mode Gauss-like spectrum $H^{M}\left(v\right)$ (57) and model $H_{K}^{M}\left(v,{\bar{g}}_{N,K}^{\lambda }\right)$ (10) with the Legendre basis functions $h_{k}\left(v\right)$ (A12), with time-scale factor α=15 s and K=12 model components, determined for the regularization parameter $\lambda =1\times 10^{-6}\;s^{-1}$: empirical index $Q_{N}\left({\bar{g}}_{N,K}^{\lambda }\right)$ (19), integral index $Q\left({\bar{g}}_{N,K}^{\lambda }\right)$ (29), mean relative error of the modulus approximation $Q_{Nrel}\left({\bar{g}}_{N,K}^{\lambda }\right)$ (47), norm ${\left\|{\bar{g}}_{N,K}^{\lambda }\right\|}_{2}$, and relative square error ERR (48) of the sampling time-independent parameter ${\bar{g}}_{K}^{*\lambda }$ approximation for N measurements corrupted by additive independent noises of the normal distribution of σ=0.001 kPa.

N	$Q_{N}\left({\bar{g}}_{N,K}^{\lambda }\right)$ [kPa^2^]	$Q\left({\bar{g}}_{N,K}^{\lambda }\right)$ [kPa^2^]	$Q_{Nrel}\left({\bar{g}}_{N,K}^{\lambda }\right)$ [%]	${\left\|{\bar{g}}_{N,K}^{\lambda }\right\|}_{2}$ [kPa·$s^{\frac{1}{2}}$]	ERR [%]
50	4.076986 × 10^−6^	8.455236 × 10^−6^	1.05247 × 10^−2^	8.120974	0.358793
100	2.705659 × 10^−6^	2.687802 × 10^−6^	8.35536 × 10^−3^	8.247319	8.50293 × 10^−2^
200	3.383251 × 10^−6^	1.898586 × 10^−6^	1.29264 × 10^−2^	8.146651	0.521292
300	2.593473 × 10^−6^	1.700482 × 10^−6^	5.91079 × 10^−3^	8.289512	5.254343 × 10^−3^
500	2.460837 × 10^−6^	1.570943 × 10^−6^	7.83666 × 10^−3^	8.302449	1.119529 × 10^−3^
1000	2.347083 × 10^−6^	1.275412 × 10^−6^	5.84073 × 10^−3^	8.313084	1.507565 × 10^−3^
3000	2.353149 × 10^−6^	1.411796 × 10^−6^	7.82569 × 10^−3^	8.305088	6.252987 × 10^−5^
5000	2.290919 × 10^−6^	1.291623 × 10^−6^	7.52114 × 10^−3^	8.312034	1.631175 × 10^−3^
10,000	2.291785 × 10^−6^	1.305035 × 10^−6^	6.40060 × 10^−3^	8.311246	1.507777 × 10^−3^
15,000	2.320758 × 10^−6^	1.387944 × 10^−6^	6.83044 × 10^−3^	8.306159	6.210229 × 10^−4^
20,000	2.377215 × 10^−6^	1.517807 × 10^−6^	7.15767 × 10^−3^	8.298835	1.838605 × 10^−4^
21,000	2.385541 × 10^−6^	1.521892 × 10^−6^	7.33644 × 10^−3^	8.298595	2.388146 × 10^−4^
23,000	2.381347 × 10^−6^	1.499912 × 10^−6^	7.19657 × 10^−3^	8.299756	1.625002 × 10^−4^
25,000	2.364198 × 10^−6^	1.448046 × 10^−6^	6.85891 × 10^−3^	8.302679	2.079057 × 10^−5^

**Table 3:** The results of simulations for double-mode Gauss-like relaxation spectrum $H\left(\tau \right)$ (60) and model $H_{K}\left(\tau ,{\bar{g}}_{N,K}^{\lambda }\right)$ (9) for the power exponential basis functions $h_{k}\left(\tau \right)$ (A1) and (A2) of the time-scale parameter $\alpha =0.04\;s^{-1}$ and K=12 model components: mean-square index $Q_{N}\left({\bar{g}}_{N,K}^{\lambda }\right)$ (19), integral approximation index $Q\left({\bar{g}}_{N,K}^{\lambda }\right)$ (29), mean relative error of the modulus approximation $Q_{Nrel}\left({\bar{g}}_{N,K}^{\lambda }\right)$ (47), norm ${\left\|{\bar{g}}_{N,K}^{\lambda }\right\|}_{2}$, and square error ERR (48) of the sampling-times-independent parameter ${\bar{g}}_{K}^{*\lambda }$ approximation for N measurements disturbed by the zero-mean normal-distribution noises of σ=0.002 Pa and regularization parameter $\lambda =3\times 10^{-6}$.

N	$Q_{N}\left({\bar{g}}_{N,K}^{\lambda }\right)$ [Pa^2^]	$Q\left({\bar{g}}_{N,K}^{\lambda }\right)$ [Pa^2^]	$Q_{Nrel}\left({\bar{g}}_{N,K}^{\lambda }\right)$ [%]	${\left\|{\bar{g}}_{N,K}^{\lambda }\right\|}_{2}$ [Pa]	ERR [%]
100	1.485209 × 10^−5^	2.387557 × 10^−5^	1.40436	1.659384	15.39930
300	1.091803 × 10^−5^	1.082619 × 10^−5^	1.30587	1.888263	41.81229
500	1.038882 × 10^−5^	2.231214 × 10^−5^	1.33031	1.835471	37.53054
1000	2.163159 × 10^−5^	1.628878 × 10^−5^	1.69906	1.725519	1.33829
1500	2.224938 × 10^−5^	1.523439 × 10^−5^	1.70042	1.725772	2.62826
2000	1.653556 × 10^−5^	1.160999 × 10^−5^	1.63416	1.767956	18.03116
5000	2.160608 × 10^−5^	1.927908 × 10^−5^	1.68327	1.847164	0.99956
10,000	2.046175 × 10^−5^	2.005121 × 10^−5^	1.73742	1.834072	1.05393
20,000	2.082989 × 10^−5^	1.816824 × 10^−5^	1.70092	1.775611	0.01183
25,000	2.081715 × 10^−5^	1.814896 × 10^−5^	1.69557	1.773708	0.01878

**Table 4:** The results of simulations for the BSW relaxation frequency spectrum $H^{M}\left(v\right)$ (63) and the series model $H_{K}^{M}\left(v,{\bar{g}}_{N,K}^{\lambda }\right)$ (10) given by Laguerre basis functions $h_{k}\left(v\right)$ (A20) of the time-scale factor α=8000 s and K=14 model components: mean-square identification index $Q_{N}\left({\bar{g}}_{N,K}^{\lambda }\right)$ (19), integral approximation index $Q\left({\bar{g}}_{N,K}^{\lambda }\right)$ (29), mean relative error of the relaxation modulus approximation $Q_{Nrel}\left({\bar{g}}_{N,K}^{\lambda }\right)$ (47), norm of the optimal model parameter ${\left\|{\bar{g}}_{N,K}^{\lambda }\right\|}_{2}$, and relative square error ERR (48) of the sampling-times-independent model parameter ${\bar{g}}_{K}^{*\lambda }$ approximation for N measurements corrupted by zero-mean normal-distribution noises of standard deviation σ=0.0005 MPa; regularization parameter $\lambda =3.2\times 10^{-11}s^{-1}$.

N	$Q_{N}\left({\bar{g}}_{N,K}^{\lambda }\right)$ [MPa^2^]	$Q\left({\bar{g}}_{N,K}^{\lambda }\right)$ [MPa^2^]	$Q_{Nrel}\left({\bar{g}}_{N,K}^{\lambda }\right)$ [%]	${\left\|{\bar{g}}_{N,K}^{\lambda }\right\|}_{2}$ [MPa·$s^{\frac{1}{2}}$]	ERR [%]
100	3.755918 × 10^−7^	1.062185 × 10^−5^	59.43101	49.287263	23.309788
200	3.071982 × 10^−7^	2.77500 × 10^−5^	94.764784	48.857821	20.045016
300	3.173889 × 10^−7^	4.678889 × 10^−6^	3.08653	55.191095	13.022671
400	2.704933 × 10^−7^	1.352739 × 10^−6^	1.91174	56.503683	5.213542
500	2.671081 × 10^−7^	1.306293 × 10^−6^	7.78426	56.362120	5.057555
1000	2.688764 × 10^−7^	6.204845 × 10^−7^	0.95187	57.343444	1.602974
2000	2.908708 × 10^−7^	1.200322 × 10^−6^	0.99676	56.541699	3.637514
5000	2.935320 × 10^−7^	1.093061 × 10^−7^	0.69706	58.286523	0.478228
7500	2.944056 × 10^−7^	8.405738 × 10^−8^	0.68399	58.046106	0.328762
10,000	2.935208 × 10^−7^	7.281316 × 10^−8^	0.69958	57.985719	0.244127
20,000	2.917343 × 10^−7^	8.063323 × 10^−8^	0.70585	58.139018	0.375008
22,000	2.931638 × 10^−7^	8.068324 × 10^−8^	0.711435	58.128401	0.367621
25,000	2.911255 × 10^−7^	7.895054 × 10^−8^	0.761685	58.228209	0.358935

## Data Availability

The original contributions presented in this study are included in the article and Supplementary Materials. Further inquiries can be directed to the corresponding author.

## Supplementary Materials

The following supporting information can be downloaded at: https://www.mdpi.com/article/10.3390/ma18184403/s1, Supplementary Materials, Section S.1. Relaxation Frequency Spectrum Model Using Chebyshev Functions; Section S.2. Relaxation Frequency Spectrum Model Using Error Functions; Section S.3. Results of the KWW Relaxation Spectrum Identification; Section S.4. Results of the Uni-Mode Gauss-Like Relaxation Spectrum Identification; Section S.5. Results of the Double-Mode Gauss-Like Relaxation Spectrum Identification; Section S.6. Results of the Baumgaertel, Schausberger, and Winter Relaxation Spectrum Identification.

## Appendix A

### Appendix A.1. Relaxation Time Spectrum Model Using Bessel Functions

Following [17], consider the following basis functions of $H_{K}\left(\tau ,g_{K}\right)$ (9):

(A1) $$h_{k}\left(\tau \right)={\left(\frac{\alpha \tau }{k-1}\right)}^{k-1}e^{-\alpha \tau +k-1},\;k=2,\;3,\;\ldots ,$$

(A2) $$h_{1}\left(\tau \right)=e^{-\alpha \tau },$$

where a time-scaling factor α>0 is expressed in $s^{-1}$. The maximum of any function $h_{k}\left(\tau \right)$ (A1) for τ≥0 is equal to 1.

According to Theorem 1 in [17], for t>0, the respective basis functions of model $G_{K}\left(t,g_{K}\right)$ (11) are as follows:

(A3) $$\phi_{k}\left(t\right)=2e^{k-1}{\left(\frac{\sqrt{\alpha t}}{k-1}\right)}^{k-1}K_{k-1}\left(2\sqrt{\alpha t}\right)\;,$$

when k≥2, the first function is given by

(A4) $$\phi_{1}\left(t\right)=2K_{0}\left(2\sqrt{\alpha t}\right)\;,$$

where $K_{k}\left(x\right)$ denotes the modified Bessel function of the second kind [75,76] and integer order k.

Note that functions $h_{k}\left(\tau \right)$ (A1), (A2) and $\phi_{k}\left(t\right)$ (A3), and (A4) are dimensionless.

The complete monotonicity and positive definiteness of $\phi_{k}\left(t\right)$ (A3) was proved in [17]. However, in view of the singularities of the Bessel functions for t→0, the following upper bounds derived in [17] (see Subsection 2.2.3, and Equations (16) and (17) in [17])

(A5) $$\phi_{1}\left(t\right)<\sqrt{\frac{\pi }{\sqrt{\alpha t}}}\;e^{-2\sqrt{\alpha t}}\;.$$

and for k≥2

(A6) $$\phi_{k}\left(t\right)<\frac{e^{k-1}}{{2\left(k-1\right)}^{k-1}}\;\frac{\Gamma \left(k-1\right)}{\sqrt{\alpha t}}.$$

are useful to estimate the norm ${\left\|\phi_{K}\left(t\right)\right\|}_{2}$; $\Gamma \left(n\right)$ denotes Euler’s gamma function [73]. According to known inequality $\Gamma \left(x\right)<x^{x-1}$ being valid for any x>1 [77] (Equation (1.5)) for k≥3 and any t∈T, we have

(A7) $$\phi_{k}\left(t\right)<\frac{e^{k-1}}{{2\left(k-1\right)}^{k-1}}\;\frac{{\left(k-1\right)}^{k-2}}{\sqrt{\alpha t_{0}}}=\frac{e^{k-1}}{2\left(k-1\right)\sqrt{\alpha t_{0}}},$$

while, from (A6), assuming that $t_{0}>0$, the following upper bound directly results for k=2 and t∈T:

(A8) $$\phi_{2}\left(t\right)<\frac{e}{2}\;\frac{\Gamma \left(1\right)}{\sqrt{\alpha t_{0}}}=\;\frac{e}{2\sqrt{\alpha t_{0}}}.$$

Since the upper bound (A5) of the first basic function $\phi_{1}\left(t\right)$ monotonically decreases for any α>0, and the upper bound in (A7) grows with increasing k≥3, combining inequalities (A5), (A7), and (A8) for t∈T yields the following property:

**Property A.1.** *Let *α>0*and *$t_{0}>0$*. The functions *$\phi_{k}\left(t\right)$*(A3), and (A4) are such that*

$$\underset{t\in T}{\sup}{\left\|\phi_{K}\left(t\right)\right\|}_{2}<M_{2}=\sqrt{\frac{\pi }{\sqrt{\alpha t_{0}}}\;e^{-4\sqrt{\alpha t_{0}}}+\frac{e^{2}}{4\alpha t_{0}}+\frac{e^{2\left(K-1\right)}\left(K-2\right)}{4{\left(K-1\right)}^{2}\alpha t_{0}}},$$

*while, for functions *$h_{k}\left(\tau \right)$ *(A1) and (A2), we have*

$$\underset{\tau \geq 0}{\sup}{\left\|k_{K}\left(\tau \right)\right\|}_{2}{=M}_{\tau }=K<\infty ,$$

*where Equation (18) defines the vector functions *$\phi_{K}\left(t\right)$ *and *$k_{K}\left(\tau \right).$

### Appendix A.2. Relaxation Frequency Spectrum Model Using Exponential Functions

For k=0, 1, … and α>0, the set of functions $\left\{e^{-\alpha kv}\right\}$ form a basis of the space $L^{2}\left(0,\right.\left.\infty \right)$ [32,78]. The modified spectrum $H^{M}\left(v\right)\in L^{2}\left(0,\right.\left.\infty \right)$, from which $H^{M}\left(v\right)\to 0$ as v→∞; therefore, we can neglect the first constant basis function. The basis functions of $H_{K}^{M}\left(v,g_{K}\right)$ (10) are

(A9) $$h_{k}\left(v\right)=e^{-\alpha kv},\;\;k=1,\;2,\;\ldots$$

with a time-scaling factor α>0. The corresponding basis functions of the relaxation time spectrum model are

(A10) $$h_{k}\left(\tau \right)=e^{-\frac{\alpha k}{\tau }}\frac{1}{\tau },\;\;k=1,\;2,\ldots .$$

The basis functions of models $G_{K}\left(t,g_{K}\right)$ (11) and (12) are given by the following formula derived in [32]

(A11) $$\phi_{k}\left(t\right)=\frac{1}{t+\alpha k}.$$

In this case, functions $h_{k}\left(v\right)=e^{-\alpha kv}$ are dimensionless, while the unit of $h_{k}\left(\tau \right)$ and $\phi_{k}\left(t\right)$ is $s^{-1}$.

Fulfillment of Assumptions 3 and 4 is resolved by the following property, where the boundness of ${\left\|k_{K}\left(\tau \right)\right\|}_{2}$ follows from the monotonicity of function $h_{k}\left(\tau \right)$ (A10), having unique maxima $1/\left(\alpha ke\right)$ at τ=αk, and which tends to zero for τ→0 and τ→∞; see also Figure 3 in [32].

**Property A.2.** 
*Let* α>0 *and* $t_{0}=0$ *. The functions* $h_{k}\left(v\right)$ *(A9),* $h_{k}\left(\tau \right)$ *(A10), and* $\phi_{k}\left(t\right)$ *(A11) are such that*

$$\underset{v\geq 0}{\sup}{\left\|h_{K}\left(v\right)\right\|}_{2}<M_{v}=K,$$

$$\underset{\tau \geq \tau_{0}>0}{\sup}{\left\|k_{K}\left(\tau \right)\right\|}_{2}{=M}_{\tau }=\frac{1}{\alpha e}\sqrt{\sum_{k=1}^{K}\frac{1}{k^{2}}}<\frac{K}{\alpha e}<\infty ,$$

$$\underset{t\in T}{\sup}{\left\|\phi_{K}\left(t\right)\right\|}_{2}<M_{2}=\frac{1}{\alpha }\sqrt{\sum_{k=1}^{K}\frac{1}{k^{2}}}<\frac{K}{\alpha }<\infty .$$

### Appendix A.3. Relaxation Frequency Spectrum Model Using Legendre Functions

Following [16], let us consider the basis functions

(A12) $$h_{k}\left(v\right)=\sqrt{2\alpha \left(2k-1\right)}\;e^{-\alpha v}P_{k-1}\left(1-2e^{-2\alpha v}\right),\;k=1,\;2,\;\ldots ,$$

where α is the time-scaling factor expressed in seconds and $P_{k}\left(x\right)$ are Legendre polynomials [79,80,81] given by recurrence Bonnet’s formula [80,81]:

$$P_{k+1}\left(x\right)=\frac{2k+1}{k+1}xP_{k}\left(x\right)-\frac{k}{k+1}P_{k-1}\left(x\right),\;k=1,\;2,\;\ldots ,$$

starting with two first polynomials:

(A13) $$P_{0}\left(x\right)=1,\;P_{1}\left(x\right)=x,$$

and forming an orthonormal basis in $L^{2}\left(0,\right.\left.\infty \right)$.

Functions $h_{k}\left(v\right)$ (A12), as the product of the polynomial of exponential argument $\left(1-2e^{-2\alpha v}\right)$, and the exponential function $e^{-\alpha v}$ tend to zero for v→∞. For v=0, by (A12) and the first equality in (A13), we have $h_{1}\left(0\right)=\sqrt{2\alpha }$, while, for k≥2, Equations (A12) and the second equality in (A13) imply $h_{k}\left(0\right)=-\sqrt{2\alpha \left(2k-1\right)}\;$. Function $h_{1}\left(v\right)$ monotonically decreases, while, for k≥2, functions $h_{k}\left(v\right)$ have local minima and maxima (c.f., Figure 1(a,b) in the paper [16]). Functions $h_{k}\left(v\right)$ (A12) are continuous for v≥0; therefore, allowing the above there exists $M_{v}$ such that

(A14) $$\underset{v\geq 0}{\sup}{\left\|h_{K}\left(v\right)\right\|}_{2}<M_{v}<\infty .$$

The basis functions $\phi_{k}\left(t\right)$ (14) related to $h_{k}\left(v\right)$ (A12) are as follows (for derivation, see [16], Appendix A.1):

(A15) $$\phi_{k}\left(t\right)=\sqrt{2\alpha \left(2k-1\right)}\;\frac{\prod_{i=0}^{k-2}\left[\left(2i+1\right)\alpha -t\right]}{\prod_{i=0}^{k-1}\left[\left(2i+1\right)\alpha +t\right]},\;k=1,\;2,\;\ldots ,$$

where $\prod_{i=0}^{p}x_{i}=1$ for p<0. The Equation (A15) can be equivalently expressed as a reccursion

(A16) $$\phi_{k+1}\left(t\right)=\phi_{k}\left(t\right)\frac{\sqrt{2k+1}}{\sqrt{2k-1}}\;\frac{\left[\left(2k-1\right)\alpha -t\right]}{\left[\left(2k+1\right)\alpha +t\right]},\;\;k=1,\;2,\;\;\ldots ,$$

starting with

(A17) $$\phi_{1}\left(t\right)=\frac{\sqrt{2\alpha }}{\alpha +t}.$$

The units of $h_{k}\left(v\right)$ and $\phi_{k}\left(t\right)$ are $s^{\frac{1}{2}}$ and $s^{-\frac{1}{2}}$, respectively. The exemplary courses of $h_{k}\left(v\right)$ and $\phi_{k}\left(t\right)$ are depicted in [16] (Figure 1). For the first positive definite basis function (A17), the following obvious inequality holds:

(A18) $$\underset{t\in T}{\sup}\phi_{1}\left(t\right)\;<\frac{\sqrt{2\alpha }}{\alpha +t_{0}}<\sqrt{\frac{2}{\alpha }}\;.$$

Functions $\phi_{k}\left(t\right)$ for k≥2 and some t∈T can take negative values (compare Figure 1c,d in the paper [16]). However, for k≥1, in view of recurrence Equation (A16), we have

(A19) $$\left|\phi_{k+1}\left(t\right)\right|=\left|\phi_{k}\left(t\right)\right|\sqrt{1+\frac{2}{2k-1}}\left|1-\frac{2\alpha +2t}{\left(2k+1\right)\alpha +t}\right|\leq \left|\phi_{k}\left(t\right)\right|\sqrt{\frac{5}{3}}\left|1-2\frac{\alpha +t}{2k\alpha +\alpha +t}\right|.$$

Since, for any t≥ 0 and α>0

$$0<\frac{\alpha +t}{2k\alpha +\alpha +t}<1,$$

the absolute value in the last factor of the right-hand side of (A19) is bounded by 1. Therefore, for k≥1,we finally obtain

$$\left|\phi_{k+1}\left(t\right)\right|\leq \left|\phi_{k}\left(t\right)\right|\sqrt{\frac{5}{3}}\;,\;$$

which, combined with (A18), implies

$$\underset{t\in T}{\sup}\left|\phi_{k}\left(t\right)\right|<\sqrt{\frac{2}{\alpha }}\;{\left(\sqrt{\frac{5}{3}}\right)}^{k-1}.$$

**Property A.3.** *Let *α>0*and *$t_{0}=0$*. The functions *$\phi_{k}\left(t\right)$*(A15) are such that*

$$\underset{t\in T}{\sup}{\left\|\phi_{K}\left(t\right)\right\|}_{2}<M_{2}=\sqrt{\frac{\frac{2}{\alpha \;}\left[1-{\left(\frac{5}{3}\right)}^{K-1}\right]}{1-\frac{5}{3}}}=\sqrt{\frac{3}{\alpha \;}\left[{\left(\frac{5}{3}\right)}^{K-1}-1\right]}.$$

*while, for the functions *$h_{k}\left(v\right)$ *(A12), the inequality (A14) holds.*

### Appendix A.4. Relaxation Frequencey Spectrum Model Using Laguerre Functions

The Laguerre polynomial, defined by the formula [79]

$$L_{k}\left(v\right)=\frac{e^{\alpha v}}{\left(k-1\right)!}\frac{d^{k-1}}{dv^{k-1}}\left(v^{k-1}e^{-\alpha v}\right),\;k=1,\;2,\;\ldots$$

multiplied by the square root of $\alpha e^{-\alpha v}$ [82], defines the continuous Laguerre function, given by the formula

(A20) $$h_{k}\left(v\right)=\sqrt{\alpha }\;e^{-\alpha v/2}L_{k}\left(v\right),\;k=1,\;2,\;\ldots ,$$

where a time-scaling factor [16,83] α>0 is measured in seconds. Similarly as for Legendre functions, functions $h_{k}\left(v\right)$ (A20), as the product of exponential function $e^{-\alpha v/2}$ and the polynomials of variable v, tend to zero asymptotically as v→∞. Since $L_{k}\left(0\right)=1$, for any k≥1, $h_{k}\left(0\right)=\sqrt{\alpha }$. However, continuous functions $h_{k}\left(v\right)$ have local minima and maxima; for exemplary functions, see Figure 2a,b in [16]. In view of the above, they are absolutely bounded for any v≥0, whence the existence of positive constant $M_{v}$, such that

(A21) $$\underset{v\geq 0}{\sup}{\left\|h_{K}\left(v\right)\right\|}_{2}<M_{v}<\infty$$

follows.

In [16] (Appendix A.2), a compact formula for the functions (14) was derived as follows:

(A22) $$\phi_{k}\left(t\right)=\frac{\sqrt{\alpha }{\left(t-\frac{\alpha }{2}\right)}^{k-1}}{{\left(t+\frac{\alpha }{2}\right)}^{k}},\;k=1,\;2,\;\ldots .$$

wherein the courses were plotted in [16] (Figure 2c,d).

The units of $h_{k}\left(v\right)$ and $\phi_{k}\left(t\right)$ are $s^{\frac{1}{2}}$ and $s^{-\frac{1}{2}}$, respectively.

Since, for $t\geq \frac{\alpha }{2}$, the basis functions are positive for any k, the following sequence of simple inequalities holds:

(A23) $$\phi_{k}\left(t\right)<\frac{\sqrt{\alpha }{\left(t-\frac{\alpha }{2}\right)}^{k-1}}{t^{k}}\leq \frac{\sqrt{\alpha }{\;t}^{k-1}}{t^{k}}=\frac{\sqrt{\alpha }}{t}\leq \frac{\sqrt{\alpha }}{\frac{\alpha }{2}}=\frac{2}{\sqrt{\alpha }},\;k=1,\;2,\;\ldots .$$

For $t<\frac{\alpha }{2}$, and even k, the basis functions (A22) are negative. Then

(A24) $$\left|\phi_{k}\left(t\right)\right|=\frac{\sqrt{\alpha }{\left(\frac{\alpha }{2}-t\right)}^{k-1}}{{\left(t+\frac{\alpha }{2}\right)}^{k}}\leq \frac{\sqrt{\alpha }{\left(\frac{\alpha }{2}\right)}^{k-1}}{{\left(t+\frac{\alpha }{2}\right)}^{k}}<\frac{\sqrt{\alpha }{\left(\frac{\alpha }{2}\right)}^{k-1}}{{\left(\frac{\alpha }{2}\right)}^{k}}=\frac{2}{\sqrt{\alpha }}.$$

If $t<\frac{\alpha }{2}$ and odd k, the basis functions (A22) are positive and the similar sequence of upper bounds is valid, as follows:

(A25) $$\phi_{k}\left(t\right)=\frac{\sqrt{\alpha }{\left(\frac{\alpha }{2}-t\right)}^{k-1}}{{\left(t+\frac{\alpha }{2}\right)}^{k}}\leq \frac{\sqrt{\alpha }{\left(\frac{\alpha }{2}\right)}^{k-1}}{{\left(t+\frac{\alpha }{2}\right)}^{k}}<\frac{\sqrt{\alpha }{\left(\frac{\alpha }{2}\right)}^{k-1}}{{\left(\frac{\alpha }{2}\right)}^{k}}=\frac{2}{\sqrt{\alpha }}.$$

Since the upper bounds in (A23)–(A25) are identical, the next property is obvious.

**Property A.4.** *Let α>0* *and $t_{0}=0$**. For the functions *$h_{k}\left(v\right)$*(A20), the upper bound (A21) is satisfied, while, for the basis functions $\phi_{k}\left(t\right)$* *(A22) of the models *$G_{K}\left(t,g_{K}\right)$*(12), the estimation*

$$\underset{t\in T}{\sup}{\left\|\phi_{K}\left(t\right)\right\|}_{2}<M_{2}=\frac{2}{\sqrt{\alpha }}$$

*holds.*

### Appendix A.5. Proof of Proposition 1

For the optimal ${\bar{g}}_{N,K}^{\lambda }$ (24), which solves the un-constrained minimization task (23), we have

(A26) $${\left\|{\bar{g}}_{N,K}^{\lambda }\right\|}_{2}^{2}={\bar{G}}_{N}^{T}{\Phi_{N,K}\left(\Phi_{N,K}^{T}\Phi_{N,K}+\lambda {NI}_{K,K}\right)}^{-2}\Phi_{N,K}^{T}{\bar{G}}_{N},$$

whence, using the right-hand side of the Rayleigh–Ritz inequalities [84]:

$$\lambda_{min}\left(A\right)w^{T}w\leq w^{T}Aw\leq \lambda_{max}\left(A\right)w^{T}w,$$

which are valid for an arbitrary $w\epsilon R^{K}$ and symmetric matrix $A=A^{T}\epsilon R^{K,K}$, with $\lambda_{min}\left(A\right)$ and $\lambda_{max}\left(A\right)$ denoting, respectively, the minimal and maximal eigenvalues of A, we obtain

(A27) $${\left\|{\bar{g}}_{N,K}^{\lambda }\right\|}_{2}^{2}\leq \;\lambda_{max}\left[{\left(\Phi_{N,K}^{T}\Phi_{N,K}+\lambda NI_{K,K}\right)}^{-2}\right]{\bar{G}}_{N}^{T}\Phi_{N,K}\Phi_{N,K}^{T}{\bar{G}}_{N}.$$

Since

(A28) $$\lambda_{max}\left[{\left(\Phi_{N,K}^{T}\Phi_{N,K}+\lambda NI_{K,K}\right)}^{-2}\right]=\frac{1}{\lambda_{min}\left[{\left(\Phi_{N,K}^{T}\Phi_{N,K}+\lambda NI_{K,K}\right)}^{2}\right]}$$

and for positive definite symmetric matrix ${\left(\Phi_{N,K}^{T}\Phi_{N,K}+\lambda NI_{K,K}\right)}^{2}$, the relation holds

$${\left(\Phi_{N,K}^{T}\Phi_{N,K}+\lambda NI_{K,K}\right)}^{2}\geq \lambda^{2}N^{2}I_{K,K},$$

which, in particular, means that

$$\lambda_{min}\left[{\left(\Phi_{N,K}^{T}\Phi_{N,K}+\lambda NI_{K,K}\right)}^{2}\right]\geq \lambda^{2}N^{2},$$

with (A28), the maximal eigenvalue of ${\left(\Phi_{N,K}^{T}\Phi_{N,K}+\lambda NI_{K,K}\right)}^{-2}$ can be estimated by

(A29) $$\lambda_{max}\left[{\left(\Phi_{N,K}^{T}\Phi_{N,K}+\lambda NI_{K,K}\right)}^{-2}\right]\leq \frac{1}{\lambda^{2}N^{2}}.$$

Simultaneously,

(A30) $${\bar{G}}_{N}^{T}\Phi_{N,K}\Phi_{N,K}^{T}{\bar{G}}_{N}={\left\|\Phi_{N,K}^{T}{\bar{G}}_{N}\right\|}_{2}^{2}\leq {\left\|\Phi_{N,K}^{T}\right\|}_{F}^{2}{\;\left\|{\bar{G}}_{N}\right\|}_{2}^{2},$$

with the square of the Frobenius norm ${\left\|\cdot \right\|}_{F}$ of $\Phi_{N,K}^{T}$ (21) given by

$${\left\|\Phi_{N,K}^{T}\right\|}_{F}^{2}=\sum_{i=1}^{N}\sum_{k=1}^{K}{\left|\phi_{k}\left(t_{i}\right)\right|}^{2},$$

which, under Assumption 3, can be estimated by

(A31) $${\left\|\Phi_{N,K}^{T}\right\|}_{F}^{2}\leq \sum_{i=1}^{N}M_{2}^{2}=NM_{2}^{2}.$$

By Assumptions 1 and 2, we have $\left|\bar{G}\left(t_{i}\right)\right|\leq M_{1}+\;\delta$ for i=1, …,N, whence

(A32) $${\;\left\|{\bar{G}}_{N}\right\|}_{2}^{2}\leq \sum_{i=1}^{N}{\left(M_{1}+\delta \right)}^{2}=N{\left(M_{1}+\delta \right)}^{2}.$$

Combining inequalities (A27) and (A29)–(A32), we obtain

$${\left\|{\bar{g}}_{N,K}^{\lambda }\right\|}_{2}^{2}\leq \;\frac{1}{\lambda^{2}}M_{2}^{2}{\left(M_{1}+\delta \right)}^{2}=M_{\lambda }^{2},$$

whence definition (26) of the constant $M_{3}$ directly follows, which completes the proof of the proposition.

## Appendix B

### Appendix B.1. Symbols

$G\left(t\right)$	relaxation modulus, Equations (1) and (2), Pa
$G\left(t_{i}\right)$	modulus arisen in the experiment for i=1,…,N, Pa
$\bar{G}\left(t_{i}\right)$	measurement of modulus $G\left(t_{i}\right)$ corrupted by noise, i=1,…,N, Pa
$G_{K}\left(t,g_{K}\right)$	model of the modulus $G\left(t\right)$, Equations (11) and (12), Pa
${\bar{G}}_{N}$	vector of the measurements $\bar{G}\left(t_{i}\right)$, Equation (21)
$g_{k}$	parameters of models $H_{K}\left(\tau ,g_{K}\right)$, $H_{K}^{M}\left(v,g_{K}\right)$, and $G_{K}\left(t,g_{K}\right)$
$g_{K}$	K-element vector of model parameters $g_{k}$
${\bar{g}}_{N,K}^{\lambda }$	vector (24) of optimal model parameters
${\bar{g}}_{K}^{*\lambda }$	optimal regularized sampling-times-independent model parameter, Equation (31)
G	compact subset of the space $R^{K}$ defined by Equation (25)
$H\left(\tau \right)$	spectrum of relaxation times τ, Equation (2), Pa
$H_{K}\left(\tau ,g_{K}\right)$	spectrum $H\left(\tau \right)$ model, Equation(9), Pa
$H\left(v\right)$	continuous spectrum of relaxation frequencies v, Equation (2), Pa
$H^{M}\left(v\right)$	modified relaxation spectrum, Equation (4), Pa·s
$H_{K}^{M}\left(v,g_{K}\right)$	spectrum $H^{M}\left(v\right)$ model, Equation (10), Pa·s
$h_{k}\left(v\right)$	basis functions of the model $H_{K}^{M}\left(v,g_{K}\right)$, Equation (10)
$h_{K}\left(v\right)$	vector of basis functions $h_{k}\left(v\right)$, Equation (18)
$h_{k}\left(\tau \right)$	basis functions of $H_{K}\left(\tau ,g_{K}\right)$, Equation (9)
$k_{K}\left(\tau \right)$	vector of basis functions $h_{k}\left(\tau \right)$, Equation (18)
$I_{K,K}$	identity matrix K×K, Equation (24)
K	number of models $H_{K}^{M}\left(v,g_{K}\right)$, $H_{K}\left(\tau ,g_{K}\right),$ and $G_{K}\left(t,g_{K}\right)$ summands, –
$M_{0}$	upper bound of the weight function $\;\rho \left(t\right)$ introduced below Equation (29)
$M_{1}$	upper bound of the relaxation modulus $G\left(t\right)$ introduced in Assumption 1
$M_{2}$	upper bound of the norm ${\left\|\phi_{K}\left(t\right)\right\|}_{2}$ introduced in Assumption 3
$M_{\tau }$, $M_{v}$	upper bounds of the norms ${\left\|k_{K}\left(\tau \right)\right\|}_{2}$ and ${\left\|h_{K}\left(v\right)\right\|}_{2}$ introduced in Assumption 4
$M_{3}$, $M_{4}$, $\hat{M}$	positive constants defined by Equations (26), (27), and (42)
n	number of the experiment repetitions for given pair $\left(N,\sigma \right)$
N	number of measurements in the experiment, –
$0_{K}$	zero vector in $R^{K}$
$Q_{N}\left(g_{K}\right)$	model $G_{K}\left(t,g_{K}\right)$ approximation index, Equation (19), ${Pa}^{2}$
$Q\left(g_{K}\right)$	global integral relaxation modulus approximation error, Equation (29), ${Pa}^{2}$
T	interval of times for which the relaxation modulus is accessible by experiment, $T=\left[t_{0},T\right]$
$t_{i}$	sampling times applied in stress relaxation experiment, s
$v_{0}$	lower bound of relaxation frequencies v for which model $H_{K}^{M}\left(v,g_{K}\right)$ is bounded, $v_{0}\geq 0$
$z\left(t_{i}\right)$	noises in relaxation module measurements, i=1,…,N, Pa
α	time-scaling factor of functions $h_{k}\left(\tau \right)$, $h_{k}\left(v\right)$ and $\phi_{k}\left(t\right)$
λ	regularization parameter, Equation (23)
δ	upper bound of measurement noises $z\left(t_{i}\right)$ introduced in Assumption 2
$\rho \left(t\right)$	weight function of the index $Q\left(g_{K}\right)$ being a density on the set T
$\sigma^{2}$	variance of the measurement noises $z\left(t_{i}\right)$
$\tau_{0}$	lower bound of relaxation times τ for which model $H_{K}\left(\tau ,g_{K}\right)$ is bounded, $\tau_{0}\geq 0$
$\Phi_{N,K}$	N×K matrix of the elements $\phi_{k}\left(t_{i}\right)$ defined by Equation (21)
$\phi_{k}\left(t\right)$	model $G_{K}\left(t,g_{K}\right)$ basis function, Equations (13) and (14)
$\phi_{K}\left(t\right)$	vector of basis functions $\phi_{k}\left(t\right)$, Equation (18)
ERR	relative error of the sampling-times-independent parameter ${\bar{g}}_{K}^{*\lambda }$ approximation, Equation (48), %
$ERRQ_{N}$	mean error of relaxation modulus approximation defined for n-element sample, Equation (49), ${Pa}^{2}$
ERRQ	mean integral square error of the true relaxation modulus approximation, Equation (50), ${Pa}^{2}$
MERR	mean relative approximation error of parameter ${\bar{g}}_{K}^{*\lambda }$, Equation (51), %
MNORM	mean square norm of regularized optimal parameter ${\bar{g}}_{N,K}^{\lambda }$, Equation (52)

### Appendix B.2. Special Functions and Mathematical Terminology

$erfc\left(x\right)$	complementary error function (59)
$\Gamma \left(n\right)$	gamma function
$L^{2}\left(0,\right.\left.\infty \right)$	space of real-valued functions square-integrable on the interval $\left(0,\right.\left.\infty \right)$
$R^{K}$	K-dimensional real Euclidean space
${\left\|\cdot \right\|}_{2}$	quadratic norm in space $R^{K}$
$1_{T}\left(t\right)$	indicator function of a subset T of the set R
w.p.1	with probability one
$\underset{x\in X}{min}f\left(x\right)$	find x∈X, which minimizes the function $f\left(x\right)$
$\underset{x\in X}{sup}f\left(x\right)$	supremum of the function $f\left(x\right)$ on the set X
arg minx∈Xfx	the value of x∈X at which $f\left(x\right)$ reaches minimal value

### Appendix B.3. Acronyms

BSW	Baumgaertel–Schausberger–Winter relaxation model
GCV	generalized cross-validation
KWW	Kohlrausch–Williams–Watts relaxation model
SVD	singular value decomposition, Equation (43)
