# Supplementary material for: On Sampling-Times-Independent Identification of Relaxation Time and Frequency Spectra Models of Viscoelastic Materials Using Stress Relaxation Experiment Data
Source: Materials (Basel). 2025 Sep 21;18(18):4403. doi: 10.3390/ma18184403 (PMC12471844; doi:10.3390/ma18184403)
Supplement: Supplementary file 1 [file materials-18-04403-s001.zip › materials-3790560-supplementary.pdf]

materials

MDPI

*Supplementary Materials*

# On Sampling-Times-Independent Identification of Relaxation Time and Frequency Spectra Models of Viscoelastic Materials Using Stress Relaxation Experiment Data

**Anna Stankiewicz [1,*], Sławomir Juściński [2] and Marzena Błażewicz-Woźniak [3]**

[1] Department of Technology Fundamentals, Faculty of Production Engineering, University of Life Sciences in Lublin, 20-612 Lublin, Poland
[2] Department of Power Engineering and Transportation, Faculty of Production Engineering, University of Life Sciences in Lublin, 20-612 Lublin, Poland; slawomir.juscinski@up.lublin.pl
[3] Institute of Horticultural Production, University of Life Sciences in Lublin, 20-612 Lublin, Poland; marzena.wozniak@up.lublin.pl
* **Correspondence:** anna.m.stankiewicz@gmail.com

## S.1. Relaxation Frequency Spectrum Model Using Chebyshev Functions

The Chebyshev functions, defined as

$$h_k(v) = 2\sqrt{\frac{\alpha}{\pi}}\,(e^{2\alpha v}-1)^{-1/4}\,T_k(1-2e^{-2\alpha v}),\ k = 2,3,\ldots, \tag{S1}$$

**Copyright:** © 2025 by the authors. Licensee MDPI, Basel, Switzerland. This article is an open access article distributed under the terms and conditions of the Creative Commons Attribution (CC BY) license (https://creativecommons.org/licenses/by/4.0/).

where $T_k(x)$ are Chebyshev polynomials of the first kind defined by the following recursion relation [82,83]:

$$T_k(x) = 2xT_{k-1}(x) - T_{k-2}(x),\ k = 3,4,\ldots, \tag{S2}$$

starting with

$$T_1(x) = 1, \qquad T_2(x) = x, \tag{S3}$$

with the first function defined as

$$h_1(v) = \sqrt{\frac{2\alpha}{\pi}}\,(e^{2\alpha v}-1)^{-1/4}, \tag{S4}$$

form an orthonormal basis in the space $\mathbb{L}^2(0,\infty)$ [16,82,83]. A positive time-scaling factor $\alpha$ is expressed in seconds.

The first basis function $h_1(v)$ (S4) monotonically exponentially decreases to zero. The next basis function $h_k(v)$, by (S1) and (S4), can be expressed as

$$h_k(v) = \sqrt{2}\,h_1(v)\,T_k(1-2e^{-2\alpha v}),\ k = 2,3,\ldots, \tag{S5}$$

where polynomials $T_k(1-2e^{-2\alpha v})$ of the exponentially decreasing argument $(1-2e^{-2\alpha v})$ tends for $v\to\infty$ to 1 for odd $k$ and to zero whenever $k$ is even (compare formulae (S2) and (S3) for the argument $x=0$). Therefore, for $v\to\infty$ functions, $h_k(v)$ decrease to zero for any $k\geq 1$.

The basis function $h_1(v)$ (S4) is singular for $v=0$. According to (S2) and (S3), for $v=0$, we have $T_{k+2}(0) = (-1)T_k(0)$, starting from $T_1(0)=1$, whenever $k$ is odd and $T_k(0)=0$ for even $k$. Therefore, functions $h_k(v)$ (S5) are also singular for $v=0$, and Assumption 4 can be satisfied only for $v_0>0$. For $v\geq v_0>0$, by (S4), we have

 https://doi.org/10.3390/ma18184403

$$\sup_{v \geq v_0} h_1(v) = \sqrt{\frac{2\alpha}{\pi}}\,(e^{2\alpha v_0} - 1)^{-1/4} = m_1. \tag{S6}$$

Simultaneously, for any $v \geq v_0 > 0$, the argument $(1 - 2e^{-2\alpha v})$ of the Chebyshev polynomials are such that

$$1 - 2e^{-2\alpha v_0} \leq 1 - 2e^{-2\alpha v} \leq 1, \tag{S7}$$

therefore, by (S2), for any $v \geq v_0 > 0$ and $k = 3,4, \dots, K$, we have

$$|T_k(1 - 2e^{-2\alpha v})| \leq 2|T_{k-1}(1 - 2e^{-2\alpha v})| + |T_{k-2}(1 - 2e^{-2\alpha v})|,\ k = 3,4, \dots,$$

whence

$$\sup_{v \geq v_0} |T_k(1 - 2e^{-2\alpha v})| = m_k = 2m_{k-1} + m_{k-2}. \tag{S8}$$

By (S5)-(S8), the following estimations hold:

$$\sup_{v \geq v_0} h_k(v) \leq \sqrt{2}\, m_1 m_k,\ k = 2,3, \dots,$$

with $m_k$ defined by (S6) and the recurrence formula given by the right-hand side of (S8).

It is proved in [16] (Appendix A.3) that the modulus basis functions $\phi_k(t)$ (14) are described by the recursive formula

$$\phi_k(t) = 2\phi_{k-1}(t) - \phi_{k-2}(t) - 4\phi_{k-1}(t + 2\alpha), \quad k = 4,5, \dots, \tag{S9}$$

and for $k = 1,2,3$ are given by

$$\phi_1(t) = \frac{1}{\sqrt{2\pi\alpha}} \frac{\Gamma\left(\frac{3}{4}\right)\Gamma\left(\frac{t}{2\alpha}+\frac{1}{4}\right)}{\Gamma\left(\frac{t}{2\alpha}+1\right)}, \tag{S10}$$

$$\phi_2(t) = \frac{(\alpha - t)}{2\alpha\sqrt{\pi\alpha}} \frac{\Gamma\left(\frac{3}{4}\right)\Gamma\left(\frac{t}{2\alpha}+\frac{1}{4}\right)}{\Gamma\left(\frac{t}{2\alpha}+2\right)}, \tag{S11}$$

$$\phi_3(t) = \frac{(2\alpha^2 + t^2 - 6\alpha t)}{4\alpha^2\sqrt{\pi\alpha}} \frac{\Gamma\left(\frac{3}{4}\right)\Gamma\left(\frac{t}{2\alpha}+\frac{1}{4}\right)}{\Gamma\left(\frac{t}{2\alpha}+3\right)}. \tag{S12}$$

A few first basis functions $\phi_k(t)$ are plotted in Figure 3(c,d) in [16]. Function $\phi_1(t)$ is non-negative for any argument, while the next functions $\phi_2(t)$, $\phi_3(t)$, …. change the sign.

Using the well-known property of the gamma function [77] (Equation (A.3))

$$\Gamma(x + 1) = x\Gamma(x),$$

for the second and third basis functions, we have

$$\phi_2(t) = \frac{(\alpha - t)}{2\alpha\sqrt{\pi\alpha}} \frac{\Gamma\left(\frac{3}{4}\right)\Gamma\left(\frac{t}{2\alpha}+\frac{1}{4}\right)}{\left(\frac{t}{2\alpha}+1\right)\Gamma\left(\frac{t}{2\alpha}+1\right)} = \frac{\Gamma\left(\frac{3}{4}\right)(\alpha - t)}{\sqrt{\pi\alpha}(t + 2\alpha)} \frac{\Gamma\left(\frac{t}{2\alpha}+\frac{1}{4}\right)}{\Gamma\left(\frac{t}{2\alpha}+1\right)}, \tag{S13}$$

$$\phi_3(t) = \frac{(2\alpha^2 + t^2 - 6\alpha t)}{4\alpha^2\sqrt{\pi\alpha}} \frac{\Gamma\left(\frac{3}{4}\right)\Gamma\left(\frac{t}{2\alpha}+\frac{1}{4}\right)}{\left(\frac{t}{2\alpha}+2\right)\left(\frac{t}{2\alpha}+1\right)\Gamma\left(\frac{t}{2\alpha}+1\right)} = \frac{\Gamma\left(\frac{3}{4}\right)(2\alpha^2 + t^2 - 6\alpha t)}{\sqrt{\pi\alpha}(8\alpha^2 + t^2 + 6\alpha t)} \frac{\Gamma\left(\frac{t}{2\alpha}+\frac{1}{4}\right)}{\Gamma\left(\frac{t}{2\alpha}+1\right)}. \tag{S14}$$

For real $x > x_0 \cong 1.4616 \dots$, the gamma function $\Gamma(x)$ is monotonically increasing, and monotonically decreasing for $0 < x < x_0$; see [77] (p. 295). Therefore, for $t \geq 2\alpha x_0 - \alpha/2 \cong 2.4232\alpha$, the following inequalities hold:

$$\phi_1(t) < \frac{1}{\sqrt{2\pi\alpha}}\,\Gamma\left(\frac{3}{4}\right) = \bar{m}_1, \tag{S15}$$

$$|\phi_2(t)| = \frac{\Gamma\left(\frac{3}{4}\right)(t - \alpha)}{\sqrt{\pi\alpha}(t + 2\alpha)} \frac{\Gamma\left(\frac{t}{2\alpha}+\frac{1}{4}\right)}{\Gamma\left(\frac{t}{2\alpha}+1\right)} < \frac{\Gamma\left(\frac{3}{4}\right)}{\sqrt{\pi\alpha}} = \bar{m}_2, \tag{S16}$$

$$|\phi_3(t)| = \frac{\Gamma\left(\frac{3}{4}\right)|2\alpha^2+t^2-6\alpha t|}{\sqrt{\pi\alpha}(8\alpha^2+t^2+6\alpha t)} \frac{\Gamma\left(\frac{t}{2\alpha}+\frac{1}{4}\right)}{\Gamma\left(\frac{t}{2\alpha}+1\right)} \leq \frac{\Gamma\left(\frac{3}{4}\right)(2\alpha^2+t^2+6\alpha t)}{\sqrt{\pi\alpha}(8\alpha^2+t^2+6\alpha t)} \frac{\Gamma\left(\frac{t}{2\alpha}+\frac{1}{4}\right)}{\Gamma\left(\frac{t}{2\alpha}+1\right)} < \frac{\Gamma\left(\frac{3}{4}\right)}{\sqrt{\pi\alpha}} = \overline{m}_3. \quad \text{(S17)}$$

By (S9), for $k \geq 4$, we have

$$|\phi_k(t)| \leq 2|\phi_{k-1}(t)| + |\phi_{k-2}(t)| + 4|\phi_{k-1}(t+2\alpha)|, \quad \text{(S18)}$$

which, combined with (S15)-(S17), immediately implies the upper bound valid for $t \geq 2\alpha x_0 - \alpha/2 \cong 2.4232\alpha$:

$$|\phi_4(t)| \leq 2|\phi_3(t)| + |\phi_2(t)| + 4|\phi_3(t+2\alpha)| < \frac{6\,\Gamma\left(\frac{3}{4}\right)}{\sqrt{\pi\alpha}} + \frac{\Gamma\left(\frac{3}{4}\right)}{\sqrt{\pi\alpha}}.$$

Generally, in view of (S18), for $k \geq 4$ and $t \geq 2\alpha x_0 - \alpha/2 \cong 2.4232\alpha$, the following recurrence formula holds:

$$|\phi_k(t)| < \overline{m}_k = 6\overline{m}_{k-1} + \overline{m}_{k-2}. \quad \text{(S19)}$$

In turn, for $t_0 \leq t < 2\alpha x_0 - \alpha/2 \cong 2.4232\alpha$, the gamma function $\Gamma\left(\frac{t}{2\alpha}+\frac{1}{4}\right)$ decreases monotonically from $\Gamma\left(\frac{t_0}{2\alpha}+\frac{1}{4}\right)$ to the minimum value of the gamma function for real positive argument, equal to $\Gamma(x_0) = 0.8856\ldots$; c.f., [77] (p. 295). Therefore, by (S10), (S13), and (S14), the following estimations hold for the first three basis functions:

$$\phi_1(t) \leq \frac{1}{\sqrt{2\pi\alpha}} \frac{\Gamma\left(\frac{3}{4}\right)\Gamma\left(\frac{t}{2\alpha}+\frac{1}{4}\right)}{\Gamma\left(\frac{t}{2\alpha}+1\right)} \leq \frac{\Gamma\left(\frac{3}{4}\right)\Gamma\left(\frac{t_0}{2\alpha}+\frac{1}{4}\right)}{\sqrt{2\pi\alpha}\Gamma(x_0)} = \overline{\overline{m}}_1, \quad \text{(S20)}$$

$$|\phi_2(t)| = \frac{\Gamma\left(\frac{3}{4}\right)|t-\alpha|}{\sqrt{\pi\alpha}(t+2\alpha)} \frac{\Gamma\left(\frac{t}{2\alpha}+\frac{1}{4}\right)}{\Gamma\left(\frac{t}{2\alpha}+1\right)} < \frac{\Gamma\left(\frac{3}{4}\right)\Gamma\left(\frac{t_0}{2\alpha}+\frac{1}{4}\right)}{\sqrt{\pi\alpha}\Gamma(x_0)} = \overline{\overline{m}}_2, \quad \text{(S21)}$$

$$|\phi_3(t)| = \frac{\Gamma\left(\frac{3}{4}\right)|2\alpha^2+t^2-6\alpha t|}{\sqrt{\pi\alpha}(8\alpha^2+t^2+6\alpha t)} \frac{\Gamma\left(\frac{t}{2\alpha}+\frac{1}{4}\right)}{\Gamma\left(\frac{t}{2\alpha}+1\right)} < \frac{\Gamma\left(\frac{3}{4}\right)\Gamma\left(\frac{t_0}{2\alpha}+\frac{1}{4}\right)}{\sqrt{\pi\alpha}\,\Gamma(x_0)} = \overline{\overline{m}}_3 \quad \text{(S22)}$$

since $|t-\alpha| < (t+2\alpha)$ and $|2\alpha^2+t^2-6\alpha t| < (8\alpha^2+t^2+6\alpha t)$. According to (S18), for $k \geq 4$, we have

$$|\phi_k(t)| \leq \overline{\overline{m}}_k = 2\overline{\overline{m}}_{k-1} + \overline{\overline{m}}_{k-2} + 4max\{\overline{\overline{m}}_{k-1}, \overline{m}_{k-1}\}, \quad \text{(S23)}$$

where the last component of the right-hand side of (S23) takes into account the shift of the function argument.

Let us consider two complementary cases: (a) $\frac{t_0}{2\alpha}+\frac{1}{4} \geq x_0$ and (b) $\frac{t_0}{2\alpha}+\frac{1}{4} < x_0$. In case (a), for any $t \in \mathcal{T}$, we have $t \geq 2\alpha x_0 - \alpha/2 \cong 2.4232\alpha$, and the estimations (S15), (S16), (S17), and (S19) hold for any $t \in \mathcal{T}$. In case (b), both $t \geq 2.4232\alpha$ and $t < 2.4232\alpha$ are possible for $t \in \mathcal{T}$. However, in view of the gamma function monotonicity for the real positive argument, in this case, $\Gamma\left(\frac{t_0}{2\alpha}+\frac{1}{4}\right) > \Gamma(x_0)$, whence, for the upper bounds in (S15)-(S17) and (S20)-(S22), the inequalities $\overline{m}_1 < \overline{\overline{m}}_1$, $\overline{m}_2 < \overline{\overline{m}}_2$, and $\overline{m}_3 < \overline{\overline{m}}_3$ hold. Whence, by (S19) and (S23), the inequality $\overline{m}_k < \overline{\overline{m}}_k$ holds for any $k \geq 4$. Fulfillment of Assumptions 3 and 4 is resolved by the next property.

**Property S.1.** *Let* $\alpha > 0$ *and* $t_0 = 0$. *The basis functions* $h_k(v)$ *(S1) and (A20) of the relaxation spectrum models* $H_K^M(v, \boldsymbol{g}_K)$ *(10) are such that*

$$\sup_{v \geq v_0 > 0} \|\boldsymbol{h}_K(v)\|_2 < M_v = m_1\sqrt{1 + 2\sum_{k=2}^K m_k^2} < \infty,$$

*where* $m_k$ *are given by (S6) and (S8). The basis functions* $\phi_k(t)$ *(S9)-(S12) of the relaxation modulus models* $G_K(t, \boldsymbol{g}_K)$ *(12) are such that*

$$\sup_{t \in \mathcal{T}} \|\boldsymbol{\phi}_K(t)\|_2 < M_2 = \sqrt{\sum_{k=1}^K \overline{m}_k^2} < \infty,$$

*if* $\frac{t_0}{2\alpha}+\frac{1}{4} \geq x_0$, *and*

$$\sup_{t\in\mathcal{T}}\|\boldsymbol{\phi}_K(t)\|_2 < M_2 = \sqrt{\sum_{k=1}^{K}\bar{\bar{m}}_k^2} < \infty,$$

*whenever* $\frac{t_0}{2\alpha}+\frac{1}{4}<x_0$, *with constants* $\bar{m}_k$ *and* $\bar{\bar{m}}_k$ *defined by (S15)-(S17), (S19), and (S20)-(S23), respectively.*

The unit of the basis functions $h_k(v)$ is $\mathrm{s}^{\frac{1}{2}}$, while the unit of the functions $\phi_k(t)$ is $\mathrm{s}^{-\frac{1}{2}}$.

**S.2. Relaxation Frequency Spectrum Model Using Error Functions**

The set of the linearly independent functions $\{e^{-\alpha v^2}, ve^{-\alpha v^2}, v^2e^{-\alpha v^2}, \ldots\}$ form a basis of the space $\mathbb{L}^2(0,\infty)$ [80]; here, $\alpha$ is a positive time-scaling factor, the unit of which is $\mathrm{s}^2$.

Since, for any fixed $\alpha$, the maximum of the function $\bar{h}_k(v) = v^{k-1}e^{-\alpha v^2}$:

$$\max_{v\geq 0} \bar{h}_k(v) = \left(\frac{k-1}{2\alpha}\right)^{\frac{k-1}{2}} e^{-\frac{(k-1)}{2}}$$

grows or decreases rapidly with $k$, depending on the value of parameter $\alpha$; the real modified relaxation spectrum $H^M(v)$ can be expanded into a series of normalized basis functions:

$$h_k(v) = \left(\frac{2\alpha e}{k-1}\right)^{\frac{k-1}{2}} v^{k-1}e^{-\alpha v^2},\ k = 2,3, \ldots, \tag{S24}$$

with the first monotonically decreasing function

$$h_1(v) = e^{-\alpha v^2}. \tag{S25}$$

Functions $h_k(v)$ (S24) take maximum equal to one for $v = \sqrt{(k-1)/2\alpha}$.

The basis functions $\phi_k(t)$ (14) of the relaxation modulus model are given by compact recursive–analytical formulae valid for $t \geq 0$, which results from Theorem 2 in [40], as follows:

$$\phi_{k+2}(t) = e\left[\frac{k(k+3)}{k+2}\right]^{\frac{k+2}{2}} \sqrt{k+3}\left[\frac{(k-1)^{\frac{k-1}{2}}}{(k+1)^{k+1}}\phi_k(t) - \frac{1}{\sqrt{2\alpha e}\, k\,(k+2)^{\frac{k+2}{2}}} t\phi_{k+1}(t)\right], \tag{S26}$$

for $k = 1,2, \ldots$, staring with

$$\phi_1(t) = \frac{\sqrt{\pi}}{2\sqrt{\alpha}}\, e^{\frac{t^2}{4\alpha}}\, erfc\left(\frac{t}{2\sqrt{\alpha}}\right), \tag{S27}$$

and

$$\phi_2(t) = \frac{3\sqrt{3}}{4\sqrt{\alpha}}[1 - t\,\phi_1(t)], \tag{S28}$$

where the complementary error function $erfc$ is defined by Equation (59).

The basis functions $h_k(v)$ (S24) and (S25) are dimensionless, while the unit of the functions $\phi_k(t)$ (S26)-(S28) is $\mathrm{s}^{-1}$.

Since the functions $\phi_k(t)$ (S26)-(S28) are non-negative definite and monotonically decrease for any $k \geq 1$ (for details see, [40]), from (S26)-(S28), the following estimations result:

$$\sup_{t\in\mathcal{T}} \phi_1(t) < \frac{\sqrt{\pi}}{2\sqrt{\alpha}}\, e^{\frac{t_0^2}{4\alpha}}\, erfc\left(\frac{t_0}{2\sqrt{\alpha}}\right) = \phi_1(t_0) \leq \phi_1(0) = \frac{\sqrt{\pi}}{2\sqrt{\alpha}} = \bar{p}_1, \tag{S29}$$

$$\sup_{t\in\mathcal{T}} \phi_2(t) < \frac{3\sqrt{3}}{4\sqrt{\alpha}} = \bar{p}_2, \tag{S30}$$

and for the next $k = 1,2, \ldots$, the following:

$$\sup_{t\in\mathcal{T}} \phi_{k+2}(t) \leq e\left[\frac{k(k+3)}{k+2}\right]^{\frac{k+2}{2}} \sqrt{k+3}\,\frac{(k-1)^{\frac{k-1}{2}}}{(k+1)^{k+1}} \sup_{t\in\mathcal{T}} \phi_k(t),$$

whence the next estimation immediately results as

$$\sup_{t\in\mathcal{T}} \phi_{k+2}(t) < e\left(\frac{k+3}{k+1}\right)^{\frac{k+3}{2}} \bar{p}_k = \bar{p}_{k+2}, \tag{S31}$$

where $\bar{p}_k$ denotes the upper bound of $\phi_k(t)$. Therefore, we obtain the property.

**Property S.2.** *Let $\alpha > 0$ and $t_0 = 0$. The basis functions $h_k(v)$ (S24), and (S25) of the relaxation frequency spectrum model are bounded, such that $\sup_{v \geq 0} \|\boldsymbol{h}_K(v)\|_2 \leq M_v = K$, while the respective basis functions $\phi_k(t)$ (S26)-(S28) of the relaxation modulus models $G_K(t, \boldsymbol{g}_K)$ (12) are such that*

$$\sup_{t \in \mathcal{T}} \|\boldsymbol{\phi}_K(t)\|_2 < M_2 = \sqrt{\sum_{k=1}^{K} p_k^2}.$$

*with constants $\bar{p}_k$ defined by (S29)-(S31).*

## S.3. Results of the KWW Relaxation Spectrum Identification

**Table S1.** The results of simulations for KWW relaxation spectrum $H^M(v)$ (55) and model $H_K^M(v, \overline{\boldsymbol{g}}_{N,K}^{\lambda})$ (10) with exponential basis functions $h_k(v)$ (A9) of the time-scale factor $\alpha = 1.97$ s and $K = 20$ model components, determined for the regularization parameter $\lambda = 8.3 \times 10^{-8}$ s$^{-2}$: mean-square identification index $Q_N(\overline{\boldsymbol{g}}_{N,K}^{\lambda})$ (19), integral approximation index $Q(\overline{\boldsymbol{g}}_{N,K}^{\lambda})$ (29), mean relative error of the relaxation modulus approximation $Q_{Nrel}(\overline{\boldsymbol{g}}_{N,K}^{\lambda})$ (47), norm of the optimal model parameter $\|\overline{\boldsymbol{g}}_{N,K}^{\lambda}\|_2$, and relative percentage square error $ERR$ (48) of the sampling-times-independent model parameter $\overline{\boldsymbol{g}}_K^{*\lambda}$ approximation for $N$ relaxation modulus measurements corrupted by additive independent noises of normal distribution with standard deviation $\sigma = 0.001$ MPa.

| $N$ | $Q_N(\overline{\boldsymbol{g}}_{N,K}^{\lambda})$ [MPa$^2$] | $Q(\overline{\boldsymbol{g}}_{N,K}^{\lambda})$ [MPa$^2$] | $Q_{Nrel}(\overline{\boldsymbol{g}}_{N,K}^{\lambda})$ [%] | $\|\overline{\boldsymbol{g}}_{N,K}^{\lambda}\|_2$[MPa · s] | $ERR$ [%] |
|---|---|---|---|---|---|
| 20 | $1.248143 \times 10^{-5}$ | $5.569827 \times 10^{-6}$ | 27.59499 | $1.598993 \times 10^{2}$ | $1.55854 \times 10^{-2}$ |
| 50 | $1.588021 \times 10^{-6}$ | $8.783205 \times 10^{-6}$ | 0.46631 | $1.599203 \times 10^{2}$ | $1.42317 \times 10^{-2}$ |
| 100 | $1.241948 \times 10^{-6}$ | $7.563286 \times 10^{-6}$ | 1.29355 | $1.599199 \times 10^{2}$ | $1.39765 \times 10^{-2}$ |
| 200 | $1.131179 \times 10^{-6}$ | $3.134989 \times 10^{-6}$ | 1.83037 | $1.598835 \times 10^{2}$ | $1.20438 \times 10^{-2}$ |
| 500 | $1.354730 \times 10^{-6}$ | $2.883161 \times 10^{-6}$ | 0.18378 | $1.598713 \times 10^{2}$ | $1.20850 \times 10^{-2}$ |
| 1000 | $2.377293 \times 10^{-6}$ | $2.581018 \times 10^{-6}$ | 0.30133 | $1.5987356 \times 10^{2}$ | $5.84503 \times 10^{-4}$ |
| 2000 | $1.627109 \times 10^{-6}$ | $2.4546375 \times 10^{-6}$ | 0.22764 | $1.598753 \times 10^{2}$ | $4.45994 \times 10^{-4}$ |
| 5000 | $3.119559 \times 10^{-6}$ | $2.473189 \times 10^{-6}$ | 0.21565 | $1.598734 \times 10^{2}$ | $2.79098 \times 10^{-4}$ |
| 7500 | $3.438031 \times 10^{-6}$ | $2.414961 \times 10^{-6}$ | 0.19876 | $1.598828 \times 10^{2}$ | $2.71473 \times 10^{-4}$ |
| 10,000 | $3.352109 \times 10^{-6}$ | $2.324826 \times 10^{-6}$ | 0.23625 | $1.598833 \times 10^{2}$ | $2.70389 \times 10^{-4}$ |

**Table S2.** The results of simulations for KWW relaxation spectrum $H^M(v)$ (55) and model $H_K^M(v, \overline{\boldsymbol{g}}_{N,K}^{\lambda})$ (10) with exponential basis functions $h_k(v)$ (A9) of the time-scale factor $\alpha = 1.97$ s and $K = 20$ model components, determined for the regularization parameter $\lambda = 8.3 \times 10^{-8}$ s$^{-2}$: mean-square identification index $Q_N(\overline{\boldsymbol{g}}_{N,K}^{\lambda})$ (19), integral approximation index $Q(\overline{\boldsymbol{g}}_{N,K}^{\lambda})$ (29), mean relative error of the relaxation modulus approximation $Q_{Nrel}(\overline{\boldsymbol{g}}_{N,K}^{\lambda})$ (47), norm of the optimal model parameter $\|\overline{\boldsymbol{g}}_{N,K}^{\lambda}\|_2$, and relative percentage square error $ERR$ (48) of the sampling-times-independent model parameter $\overline{\boldsymbol{g}}_K^{*\lambda}$ approximation for $N$ relaxation modulus measurements corrupted by additive independent noises of normal distribution with standard deviation $\sigma = 0.003$ MPa.

| $N$ | $Q_N(\overline{\boldsymbol{g}}_{N,K}^{\lambda})$ [MPa$^2$] | $Q(\overline{\boldsymbol{g}}_{N,K}^{\lambda})$ [MPa$^2$] | $Q_{Nrel}(\overline{\boldsymbol{g}}_{N,K}^{\lambda})$ [%] | $\|\overline{\boldsymbol{g}}_{N,K}^{\lambda}\|_2$[MPa · s] | $ERR$ [%] |
|---|---|---|---|---|---|
| 20 | $1.679984 \times 10^{-5}$ | $2.255015 \times 10^{-5}$ | 33.19006 | $1.598731 \times 10^{2}$ | $1.58489 \times 10^{-2}$ |
| 50 | $1.383166 \times 10^{-5}$ | $9.506957 \times 10^{-6}$ | 1.77762 | $1.599203 \times 10^{2}$ | $1.41458 \times 10^{-2}$ |
| 100 | $1.156068 \times 10^{-5}$ | $7.183035 \times 10^{-6}$ | 1.39796 | $1.599199 \times 10^{2}$ | $1.01364 \times 10^{-2}$ |
| 200 | $1.008093 \times 10^{-5}$ | $3.295252 \times 10^{-6}$ | 4.54929 | $1.598852 \times 10^{2}$ | $1.15350 \times 10^{-2}$ |
| 500 | $9.285899 \times 10^{-6}$ | $3.247222 \times 10^{-6}$ | 0.17252 | $1.598712 \times 10^{2}$ | $7.47103 \times 10^{-3}$ |
| 1000 | $1.047733 \times 10^{-5}$ | $2.667242 \times 10^{-6}$ | 0.16971 | $1.598736 \times 10^{2}$ | $4.69457 \times 10^{-3}$ |
| 2000 | $9.520827 \times 10^{-6}$ | $2.520071 \times 10^{-6}$ | 0.2613 | $1.598753 \times 10^{2}$ | $3.48999 \times 10^{-4}$ |

| | | | | | |
|---|---|---|---|---|---|
| 5000 | 1.092858 × $10^{-5}$ | 2.562305 × $10^{-6}$ | 0.72091 | 1.598734 × $10^{2}$ | 2.74303 × $10^{-4}$ |
| 7500 | 1.124156 × $10^{-5}$ | 2.441189 × $10^{-6}$ | 0.40045 | 1.598828 × $10^{2}$ | 2.75229 × $10^{-4}$ |
| 10,000 | 1.117308 × $10^{-5}$ | 2.468248 × $10^{-6}$ | 0.17854 | 1.598828 × $10^{2}$ | 2.70190 × $10^{-4}$ |

## S.4. Results of the Uni-Mode Gauss-Like Relaxation Spectrum Identification

**Table S3.** The results of simulations for uni-mode Gauss-like relaxation spectrum $H^M(v)$ (57) and model $H_K^M(v,\bar{g}_{N,K}^{\lambda})$ (10) with Legendre basis functions $h_k(v)$ (A12) of the time-scale factor $\alpha = 15$ s and $K = 12$ model components, determined for the regularization parameter $\lambda = 1 \times 10^{-6}$ s$^{-1}$: mean-square identification index $Q_N(\bar{g}_{N,K}^{\lambda})$ (19), integral approximation index $Q(\bar{g}_{N,K}^{\lambda})$ (29), mean relative error of the relaxation modulus approximation $Q_{Nrel}(\bar{g}_{N,K}^{\lambda})$ (47), norm of the optimal model parameter $\|\bar{g}_{N,K}^{\lambda}\|_2$, and relative percentage square error $ERR$ (48) of the sampling-times-independent model parameter $\bar{g}_K^{*\lambda}$ approximation for $N$ relaxation modulus measurements corrupted by additive independent noises of normal distribution with standard deviation $\sigma = 0.005$ kPa.

| $N$ | $Q_N(\bar{g}_{N,K}^{\lambda})$ [kPa$^2$] | $Q(\bar{g}_{N,K}^{\lambda})$ [kPa$^2$] | $Q_{Nrel}(\bar{g}_{N,K}^{\lambda})$ [%] | $\|\bar{g}_{N,K}^{\lambda}\|_2$ $\left[\text{kPa}\cdot\text{s}^{\frac{1}{2}}\right]$ | $ERR$ [%] |
|---|---|---|---|---|---|
| 50 | 3.884356 × $10^{-5}$ | 1.206949 × $10^{-5}$ | 2.69086 × $10^{-2}$ | 8.140549 | 0.52059 |
| 100 | 3.197490 × $10^{-5}$ | 5.3843033 × $10^{-6}$ | 1.08944 × $10^{-2}$ | 8.185458 | 0.51131 |
| 200 | 3.046429 × $10^{-5}$ | 1.543908 × $10^{-5}$ | 1.80479 × $10^{-2}$ | 8.181304 | 0.59600 |
| 300 | 2.893501 × $10^{-5}$ | 1.858860 × $10^{-6}$ | 1.59776 × $10^{-2}$ | 8.308257 | 3.73571 × $10^{-2}$ |
| 500 | 2.627055 × $10^{-5}$ | 1.510678 × $10^{-6}$ | 1.26583 × $10^{-2}$ | 8.336473 | 1.58612 × $10^{-2}$ |
| 1000 | 2.612684 × $10^{-5}$ | 1.167302 × $10^{-6}$ | 5.78978 × $10^{-3}$ | 8.329408 | 1.82014 × $10^{-2}$ |
| 3000 | 2.630861 × $10^{-5}$ | 1.583331 × $10^{-6}$ | 1.32897 × $10^{-2}$ | 8.297849 | 4.26832 × $10^{-4}$ |
| 5000 | 2.598222 × $10^{-5}$ | 1.495372 × $10^{-6}$ | 1.18986 × $10^{-2}$ | 8.301433 | 2.38169 × $10^{-3}$ |
| 10,000 | 2.585541 × $10^{-5}$ | 1.501461 × $10^{-6}$ | 5.31187 × $10^{-3}$ | 8.300005 | 2.38168 × $10^{-3}$ |
| 15,000 | 2.572096 × $10^{-5}$ | 1.565406 × $10^{-6}$ | 7.10237 × $10^{-3}$ | 8.296087 | 2.36219 × $10^{-3}$ |
| 20,000 | 2.588766 × $10^{-5}$ | 1.646480 × $10^{-6}$ | 7.91262 × $10^{-3}$ | 8.291693 | 6.81344 × $10^{-4}$ |
| 21,000 | 2.604104 × $10^{-5}$ | 1.639938 × $10^{-6}$ | 8.54798 × $10^{-3}$ | 8.292050 | 7.08004 × $10^{-4}$ |
| 23,000 | 2.607266 × $10^{-5}$ | 1.588047 × $10^{-6}$ | 7.84713 × $10^{-3}$ | 8.294845 | 3.24258 × $10^{-4}$ |
| 25,000 | 2.605394 × $10^{-5}$ | 1.502800 × $10^{-6}$ | 6.32227 × $10^{-3}$ | 8.299701 | 7.05732 × $10^{-5}$ |

**Table S4.** The results of simulations for uni-mode Gauss-like relaxation spectrum $H^M(v)$ (57) and model $H_K^M(v,\bar{g}_{N,K}^{\lambda})$ (10) with Legendre basis functions $h_k(v)$ (A12) of the time-scale factor $\alpha = 15$ s and $K = 12$ model components, determined for the regularization parameter $\lambda = 1 \times 10^{-6}$ s$^{-1}$: mean-square identification index $Q_N(\bar{g}_{N,K}^{\lambda})$ (19), integral approximation index $Q(\bar{g}_{N,K}^{\lambda})$ (29), mean relative error of the relaxation modulus approximation $Q_{Nrel}(\bar{g}_{N,K}^{\lambda})$ (47), norm of the optimal model parameter $\|\bar{g}_{N,K}^{\lambda}\|_2$, and relative percentage square error $ERR$ (48) of the sampling-times-independent model parameter $\bar{g}_K^{*\lambda}$ approximation for $N$ relaxation modulus measurements corrupted by additive independent noises of normal distribution with standard deviation $\sigma = 0.01$ kPa.

| $N$ | $Q_N(\bar{g}_{N,K}^{\lambda})$ [kPa$^2$] | $Q(\bar{g}_{N,K}^{\lambda})$ [kPa$^2$] | $Q_{Nrel}(\bar{g}_{N,K}^{\lambda})$ [%] | $\|\bar{g}_{N,K}^{\lambda}\|_2$ $\left[\text{kPa}\cdot\text{s}^{\frac{1}{2}}\right]$ | $ERR$ [%] |
|---|---|---|---|---|---|
| 50 | 1.447903 × $10^{-4}$ | 2.363657 × $10^{-5}$ | 0.12003 | 8.252211 | 2.962089 |
| 100 | 1.245555 × $10^{-4}$ | 2.645627 × $10^{-5}$ | 2.08568 × $10^{-2}$ | 8.134110 | 1.598159 |
| 200 | 1.151680 × $10^{-4}$ | 1.396517 × $10^{-5}$ | 3.92016 × $10^{-2}$ | 8.228408 | 1.368158 |
| 300 | 1.114154 × $10^{-4}$ | 3.619899 × $10^{-6}$ | 6.63557 × $10^{-2}$ | 8.334628 | 0.126927 |
| 500 | 1.004920 × $10^{-4}$ | 2.335035 × $10^{-6}$ | 2.46229 × $10^{-2}$ | 8.379937 | 0.104362 |
| 1000 | 1.003271 × $10^{-4}$ | 1.477660 × $10^{-6}$ | 1.42419 × $10^{-2}$ | 8.350289 | 6.577302 × $10^{-2}$ |

| | | | | | |
|---|---|---|---|---|---|
| 3000 | $1.012163 \times 10^{-4}$ | $1.925674 \times 10^{-6}$ | $2.44483 \times 10^{-2}$ | 8.288939 | $1.779348 \times 10^{-3}$ |
| 5000 | $1.000564 \times 10^{-4}$ | $1.835416 \times 10^{-6}$ | $2.09399 \times 10^{-2}$ | 8.288287 | $4.802743 \times 10^{-3}$ |
| 10,000 | $9.950314 \times 10^{-5}$ | $1.781121 \times 10^{-6}$ | $4.47750 \times 10^{-3}$ | 8.286036 | $1.315641 \times 10^{-2}$ |
| 15,000 | $9.888728 \times 10^{-5}$ | $1.806493 \times 10^{-6}$ | $7.70407 \times 10^{-3}$ | 8.283582 | $6.978478 \times 10^{-3}$ |
| 20,000 | $9.942019 \times 10^{-5}$ | $1.817854 \times 10^{-6}$ | $8.94249 \times 10^{-3}$ | 8.282891 | $1.924189 \times 10^{-3}$ |
| 21,000 | $1.000234 \times 10^{-4}$ | $1.797325 \times 10^{-6}$ | $1.02053 \times 10^{-2}$ | 8.2839879 | $1.9117074 \times 10^{-3}$ |
| 23,000 | $1.001685 \times 10^{-4}$ | $1.705401 \times 10^{-6}$ | $8.70900 \times 10^{-3}$ | 8.288836 | $6.945313 \times 10^{-4}$ |
| 25,000 | $1.0014475 \times 10^{-4}$ | $1.577016 \times 10^{-6}$ | $5.83176 \times 10^{-3}$ | 8.296134 | $2.007728 \times 10^{-4}$ |

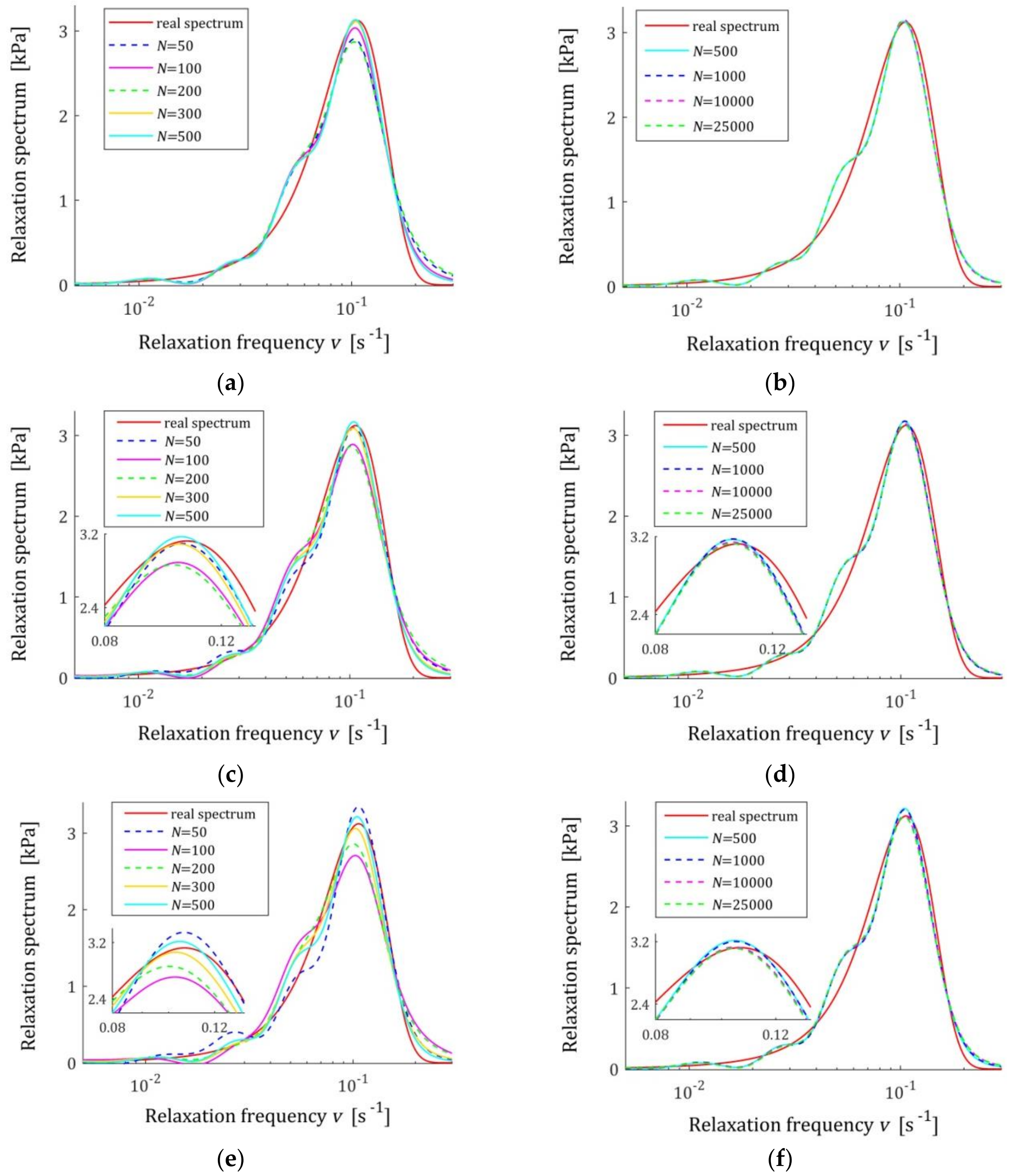


**Figure S1.** Optimal models $H_K(v, \bar{\boldsymbol{g}}_{N,K}^{\lambda}) = H_K^M(v, \bar{\boldsymbol{g}}_{N,K}^{\lambda})v$, defined by the model $H_K^M(v, \bar{\boldsymbol{g}}_{N,K}^{\lambda})$ (10), with Legendre basis functions $h_k(v)$ (A12) of the time-scale factor $\alpha = 15$ s and $K = 12$ model components of the uni-mode Gauss-like relaxation spectrum $H(v)$ (56) (solid red line), determined for the regularization parameter $\lambda = 1 \times 10^{-6}\ \mathrm{s}^{-1}$ for selected values of the number of

measurements $N$ and noises of standard deviations: (**a**,**b**) $\sigma = 0.001$ kPa; (**c**,**d**) $\sigma = 0.005$ kPa; (**e**,**f**) $\sigma = 0.01$ kPa.

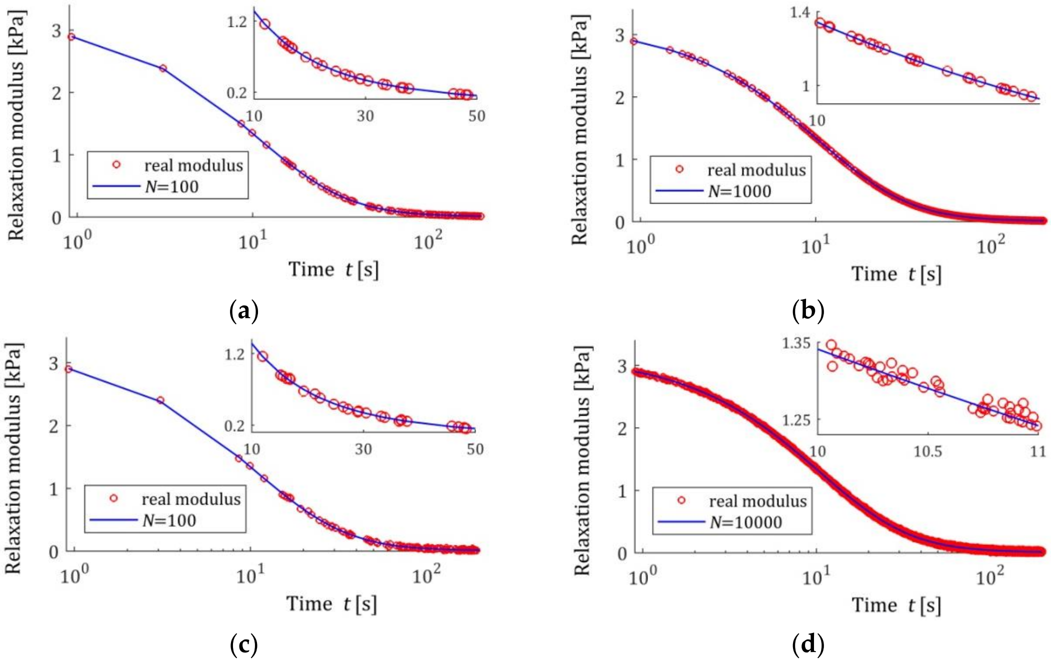


**Figure S2.** Optimal models $G_K(t,\bar{\boldsymbol{g}}_{N,K}^{\lambda})$ (15) of the relaxation modulus $G(t)$ (58), with functions $\phi_k(t)$ (A15) defined by Legendre basis functions $h_k(v)$ (A12) of the time-scale factor $\alpha = 15$ s and $K = 12$ model components, for the measurements $\bar{G}(t_i)$ marked by red points, determined for the regularization parameter $\lambda = 1 \times 10^{-6}$ s$^{-1}$ for selected values of the number of measurements $N$ and noises of standard deviations: (**a**) $\sigma = 0.001$ kPa, $N = 100$; (**b**) $\sigma = 0.001$ kPa, $N = 1000$; (**c**) $\sigma = 0.01$ kPa, $N = 100$; (**d**) $\sigma = 0.01$ kPa, $N = 10{,}000$.

## S.5. Results of the Double-Mode Gauss-Like Relaxation Spectrum Identification

**Table S5.** The results of simulations for double-mode Gauss-like relaxation spectrum $\mathcal{H}(\tau)$ (60) and the model $\mathcal{H}_K(\tau,\bar{\boldsymbol{g}}_{N,K}^{\lambda})$ (9) of power-exponential basis functions $\hbar_k(\tau)$ (A1) and (A2) of the time-scale factor $\alpha = 0.04$ s$^{-1}$ and $K = 12$ model components, determined with the regularization parameter $\lambda = 3 \times 10^{-6}$: mean-square identification index $Q_N(\bar{\boldsymbol{g}}_{N,K}^{\lambda})$ (19), integral approximation index $Q(\bar{\boldsymbol{g}}_{N,K}^{\lambda})$ (29), mean relative error of the relaxation modulus approximation $Q_{Nrel}(\bar{\boldsymbol{g}}_{N,K}^{\lambda})$ (47), norm of the optimal model parameter $\|\bar{\boldsymbol{g}}_{N,K}^{\lambda}\|_2$, and relative percentage square error $ERR$ (48) of the sampling-times-independent model parameter $\bar{\boldsymbol{g}}_K^{*\lambda}$ approximation for $N$ relaxation modulus measurements corrupted by additive independent noises of normal distribution with standard deviation $\sigma = 0.005$ Pa.

| $N$ | $Q_N(\bar{\boldsymbol{g}}_{N,K}^{\lambda})$ [Pa$^2$] | $Q(\bar{\boldsymbol{g}}_{N,K}^{\lambda})$ [Pa$^2$] | $Q_{Nrel}(\bar{\boldsymbol{g}}_{N,K}^{\lambda})$ [%] | $\|\bar{\boldsymbol{g}}_{N,K}^{\lambda}\|_2$ [Pa] | $ERR$ [%] |
|---|---|---|---|---|---|
| 100 | $4.360503 \times 10^{-5}$ | $1.645256 \times 10^{-5}$ | 1.54208 | 1.751621 | 17.50690 |
| 300 | $3.315613 \times 10^{-5}$ | $1.172133 \times 10^{-5}$ | 1.35761 | 1.917469 | 48.02838 |
| 500 | $3.026897 \times 10^{-5}$ | $2.232346 \times 10^{-5}$ | 1.36248 | 1.816546 | 36.45916 |
| 1000 | $4.277883 \times 10^{-5}$ | $1.646511 \times 10^{-5}$ | 1.73460 | 1.734257 | 0.88689 |
| 1500 | $4.468538 \times 10^{-5}$ | $1.507243 \times 10^{-5}$ | 1.71094 | 1.735802 | 1.005259 |
| 2000 | $3.816352 \times 10^{-5}$ | $1.1491724 \times 10^{-5}$ | 1.62869 | 1.775343 | 0.935363 |
| 5000 | $4.846333 \times 10^{-5}$ | $1.933840 \times 10^{-5}$ | 1.65578 | 1.844926 | 0.90564 |
| 10,000 | $4.660876 \times 10^{-5}$ | $2.007246 \times 10^{-5}$ | 1.73926 | 1.831342 | 1.02915 |
| 20,000 | $4.540960 \times 10^{-5}$ | $1.824487 \times 10^{-5}$ | 1.70935 | 1.774687 | 0.01011 |

| | | | | | |
|---|---|---|---|---|---|
| 25,000 | 4.545074 × 10⁻⁵ | 1.822641 × 10⁻⁵ | 1.70079 | 1.772688 | 0.01668 |

**Table S6.** The results of simulations for double-mode Gauss-like relaxation spectrum $\mathcal{H}(\tau)$ (60) and the model $\mathcal{H}_K(\tau,\overline{\boldsymbol{g}}_{N,K}^{\lambda})$ (9) of power-exponential basis functions $\hbar_k(\tau)$ (A1) and (A2) of the time-scale factor $\alpha = 0.04\ \mathrm{s}^{-1}$ and $K = 12$ model components, determined with the regularization parameter $\lambda = 3 \times 10^{-6}$: mean-square identification index $Q_N(\overline{\boldsymbol{g}}_{N,K}^{\lambda})$ (19), integral approximation index $Q(\overline{\boldsymbol{g}}_{N,K}^{\lambda})$ (29), mean relative error of the relaxation modulus approximation $Q_{Nrel}(\overline{\boldsymbol{g}}_{N,K}^{\lambda})$ (47), norm of the optimal model parameter $\|\overline{\boldsymbol{g}}_{N,K}^{\lambda}\|_2$, and relative percentage square error $ERR$ (48) of the sampling-times-independent model parameter $\overline{\boldsymbol{g}}_K^{*\lambda}$ approximation for $N$ relaxation modulus measurements corrupted by additive independent noises of normal distribution with standard deviation $\sigma = 0.01$ Pa.

| $\boldsymbol{N}$ | $\boldsymbol{Q_N(\overline{g}_{N,K}^{\lambda})}$ **[Pa²]** | $\boldsymbol{Q(\overline{g}_{N,K}^{\lambda})}$ **[Pa²]** | $Q_{Nrel}(\overline{\boldsymbol{g}}_{N,K}^{\lambda})$ [%] | $\|\overline{\boldsymbol{g}}_{N,K}^{\lambda}\|_2$ **[Pa]** | $\boldsymbol{ERR}$ [%] |
|---|---|---|---|---|---|
| 100 | 1.406405 × 10⁻⁴ | 2.289508 × 10⁻⁵ | 1.791218 | 1.940812 | 26.52683 |
| 300 | 1.140565 × 10⁻⁴ | 1.469913 × 10⁻⁵ | 1.448913 | 1.973891 | 59.45085 |
| 500 | 1.028987 × 10⁻⁴ | 2.317399 × 10⁻⁵ | 1.418018 | 1.785461 | 34.78308 |
| 1000 | 1.174649 × 10⁻⁴ | 1.723875 × 10⁻⁵ | 1.795107 | 1.751317 | 0.46220 |
| 1500 | 1.219549 × 10⁻⁴ | 1.511789 × 10⁻⁵ | 1.728634 | 1.753357 | 1.325442 |
| 2000 | 1.139231 × 10⁻⁴ | 1.151067 × 10⁻⁵ | 1.619612 | 1.787797 | 0.975623 |
| 5000 | 4.846333 × 10⁻⁴ | 1.951114 × 10⁻⁵ | 1.610727 | 1.841494 | 0.7748977 |
| 10,000 | 3.284507 × 10⁻⁴ | 2.014147 × 10⁻⁵ | 1.742352 | 1.826875 | 0.99747 |
| 20,000 | 2.785074 × 10⁻⁴ | 1.838739 × 10⁻⁵ | 1.723477 | 1.773160 | 8.50947 × 10⁻³ |
| 25,000 | 2.695074 × 10⁻⁴ | 1.826178 × 10⁻⁵ | 1.709533 | 1.772877 | 9.07737 × 10⁻³ |

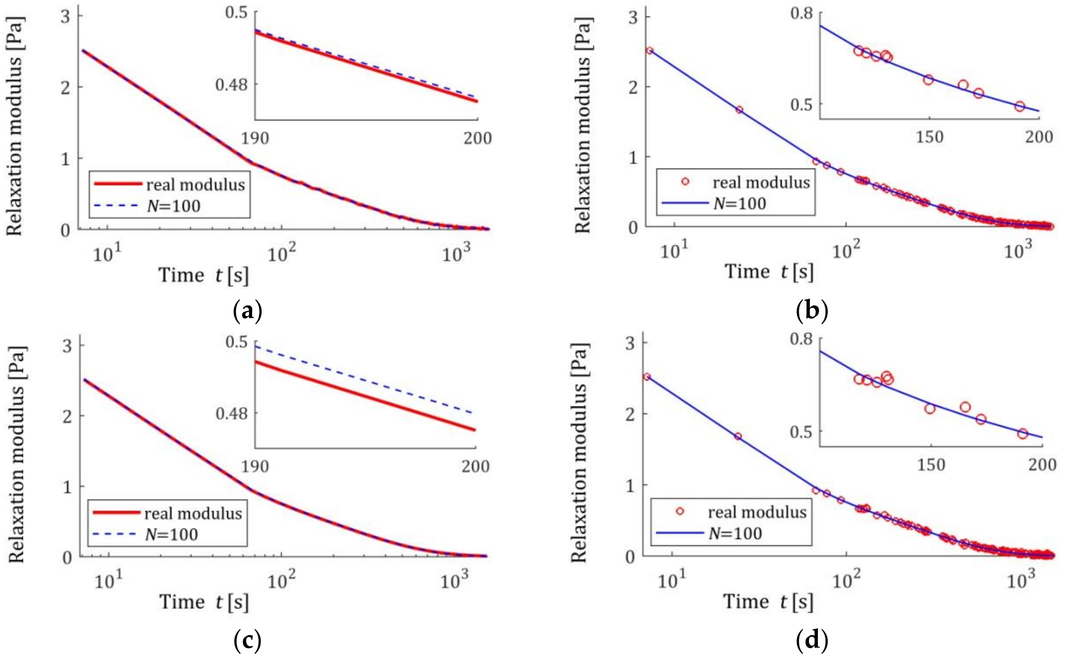


**Figure S3.** Optimal models $G_K(t,\overline{\boldsymbol{g}}_{N,K}^{\lambda})$ (15) with basis functions $\phi_k(t)$ (A3) and (A4) using Bessel functions of the time-scale factor $\alpha = 0.04\ \mathrm{s}^{-1}$ and $K = 12$ model components, determined with the regularization parameter $\lambda = 3 \times 10^{-6}$ approximating for $N = 100$ the measurements $\bar{G}(t_i)$ (red points) of the real relaxation modulus $G(t)$ (61) (red sold line) for noises of standard deviations: (**a**,**b**) $\sigma = 0.005$ Pa; (**c**,**d**) $\sigma = 0.01$ Pa.

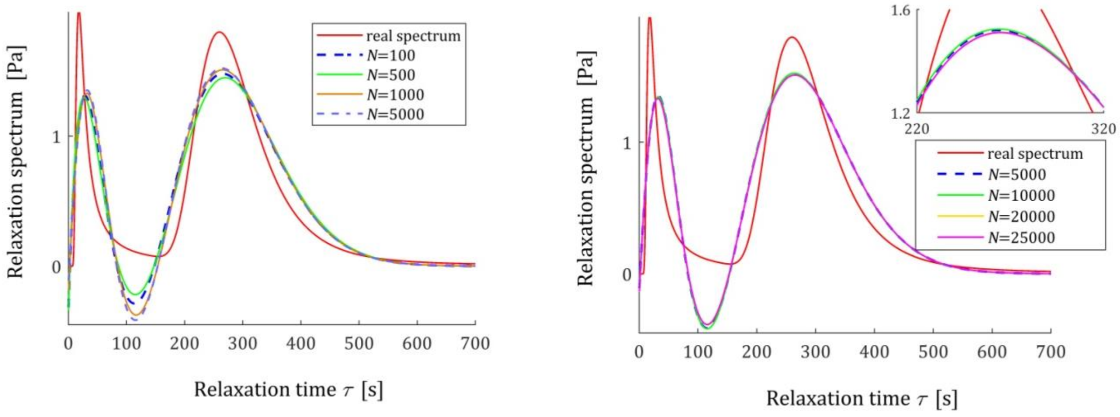


(**a**) (**b**)

**Figure S4.** Double-mode Gauss-like relaxation spectrum $\mathcal{H}(\tau)$ (60) and the model $\mathcal{H}_K(\tau, \bar{\boldsymbol{g}}_{N,K}^{\lambda})$ (9) with power-exponential basis functions $\hbar_k(\tau)$ (A1) and (A2) of the time-scale factor $\alpha = 0.04\ \mathrm{s}^{-1}$ and $K = 12$ model components, determined with the regularization parameter $\lambda = 3 \times 10^{-6}$ for noises of standard deviation $\sigma = 0.005$ Pa and selected values of the number of measurements $N$: (**a**) $N \leq 5000$; (**b**) $N \geq 5000$.

## S.6. Results of the Baumgaertel, Schausberger, and Winter Relaxation Spectrum Identification

**Table S7.** The results of simulations for BSW relaxation frequency spectrum $H^M(v)$ (63) and the series model $H_K^M(v, \bar{\boldsymbol{g}}_{N,K}^{\lambda})$ (10) given by Laguerre basis functions $h_k(v)$ (A20) of the time-scale factor $\alpha = 8000$ s and $K = 14$ model components, determined with the regularization parameter $\lambda = 3.2 \times 10^{-11}\ \mathrm{s}^{-1}$: mean-square identification index $Q_N(\bar{\boldsymbol{g}}_{N,K}^{\lambda})$ (19), integral approximation index $Q(\bar{\boldsymbol{g}}_{N,K}^{\lambda})$ (29), mean relative error of the relaxation modulus approximation $Q_{Nrel}(\bar{\boldsymbol{g}}_{N,K}^{\lambda})$ (47), norm of the optimal model parameter $\|\bar{\boldsymbol{g}}_{N,K}^{\lambda}\|_2$, and relative percentage square error $ERR$ (48) of the sampling-times-independent model parameter $\bar{\boldsymbol{g}}_K^{*\lambda}$ approximation for $N$ relaxation modulus measurements corrupted by additive independent zero-mean noises of normal distribution with standard deviation $\sigma = 0.001$ MPa.

| $N$ | $Q_N(\bar{\boldsymbol{g}}_{N,K}^{\lambda})$ [MPa$^2$] | $Q(\bar{\boldsymbol{g}}_{N,K}^{\lambda})$ [MPa$^2$] | $Q_{Nrel}(\bar{\boldsymbol{g}}_{N,K}^{\lambda})$ [%] | $\|\bar{\boldsymbol{g}}_{N,K}^{\lambda}\|_2$ $[\mathrm{MPa} \cdot \mathrm{s}^{\frac{1}{2}}]$ | $ERR$ [%] |
|---|---|---|---|---|---|
| 100 | $1.353654 \times 10^{-6}$ | $1.099095 \times 10^{-5}$ | 57.28617 | 48.347366 | 23.606876 |
| 200 | $1.136200 \times 10^{-6}$ | $5.178168 \times 10^{-6}$ | 11.61216 | 55.486986 | 14.601011 |
| 300 | $1.165213 \times 10^{-6}$ | $4.983198 \times 10^{-6}$ | 8.57459 | 55.342830 | 13.988701 |
| 400 | $1.031271 \times 10^{-6}$ | $1.568865 \times 10^{-6}$ | 6.31509 | 56.715463 | 7.256989 |
| 500 | $1.006658 \times 10^{-6}$ | $1.243756 \times 10^{-6}$ | 2.06716 | 56.348762 | 5.188832 |
| 1000 | $9.948613 \times 10^{-7}$ | $6.116877 \times 10^{-7}$ | 1.35007 | 56.940807 | 2.539559 |
| 2000 | $1.035191 \times 10^{-6}$ | $1.220737 \times 10^{-6}$ | 0.88295 | 56.502217 | 4.931318 |
| 5000 | $1.035498 \times 10^{-6}$ | $1.223309 \times 10^{-7}$ | 0.63136 | 58.508683 | 0.652769 |
| 7500 | $1.036118 \times 10^{-6}$ | $6.931035 \times 10^{-8}$ | 0.65882 | 58.116923 | 0.358382 |
| 10,000 | $1.032360 \times 10^{-6}$ | $6.310996 \times 10^{-8}$ | 0.68173 | 57.978012 | 0.364197 |
| 20,000 | $1.029144 \times 10^{-6}$ | $5.699619 \times 10^{-8}$ | 0.71162 | 58.158770 | 0.476305 |
| 22,000 | $1.031899 \times 10^{-6}$ | $5.431262 \times 10^{-8}$ | 0.77220 | 58.152254 | 0.404587 |
| 25,000 | $1.032999 \times 10^{-6}$ | $5.151278 \times 10^{-8}$ | 0.76893 | 58.319383 | 0.402530 |

**Table S8.** The results of simulations for BSW relaxation frequency spectrum $H^M(v)$ (63) and the series model $H_K^M(v, \bar{\boldsymbol{g}}_{N,K}^{\lambda})$ (10) given by Laguerre basis functions $h_k(v)$ (A20) of the time-scale factor $\alpha = 8000$ s and $K = 14$ model components, determined with the regularization parameter $\lambda = 3.2 \times 10^{-11}\ \mathrm{s}^{-1}$: mean-square identification index $Q_N(\bar{\boldsymbol{g}}_{N,K}^{\lambda})$ (19), integral approximation index

$Q(\bar{\boldsymbol{g}}_{N,K}^{\lambda})$ (29), mean relative error of the relaxation modulus approximation $Q_{Nrel}(\bar{\boldsymbol{g}}_{N,K}^{\lambda})$ (47), norm of the optimal model parameter $\|\bar{\boldsymbol{g}}_{N,K}^{\lambda}\|_2$, and relative percentage square error $ERR$ (48) of the sampling-times-independent model parameter $\bar{\boldsymbol{g}}_{K}^{*\lambda}$ approximation for $N$ relaxation modulus measurements corrupted by additive independent zero-mean noises of normal distribution with standard deviation $\sigma = 0.003$ MPa.

| $\boldsymbol{N}$ | $\boldsymbol{Q_N(\bar{g}_{N,K}^{\lambda})}$ [MPa$^2$] | $\boldsymbol{Q(\bar{g}_{N,K}^{\lambda})}$ [MPa$^2$] | $Q_{Nrel}(\bar{\boldsymbol{g}}_{N,K}^{\lambda})$ [%] | $\|\bar{\boldsymbol{g}}_{N,K}^{\lambda}\|_2$ $\left[\mathbf{MPa\cdot s^{\frac{1}{2}}}\right]$ | $\boldsymbol{ERR}$ [%] |
|---|---|---|---|---|---|
| 100 | $1.173492 \times 10^{-5}$ | $1.263655 \times 10^{-5}$ | 43.4038 | 44.660836 | 25.339363 |
| 200 | $9.906564 \times 10^{-6}$ | $6.525830 \times 10^{-6}$ | 10.82046 | 56.818537 | 20.472011 |
| 300 | $1.015736 \times 10^{-5}$ | $6.356028 \times 10^{-6}$ | 31.69834 | 56.499781 | 19.282792 |
| 400 | $9.306854 \times 10^{-6}$ | $2.677661 \times 10^{-6}$ | 17.31309 | 59.241692 | 20.859666 |
| 500 | $8.971746 \times 10^{-6}$ | $1.254865 \times 10^{-6}$ | 2.80543 | 56.299687 | 5.362578 |
| 1000 | $8.850062 \times 10^{-6}$ | $8.927024 \times 10^{-7}$ | 4.62234 | 57.542442 | 14.868648 |
| 2000 | $8.962701 \times 10^{-6}$ | $1.337214 \times 10^{-6}$ | 0.53522 | 57.601056 | 15.064858 |
| 5000 | $8.935624 \times 10^{-6}$ | $2.206824 \times 10^{-7}$ | 0.40774 | 59.6366167 | 2.297015 |
| 7500 | $8.932851 \times 10^{-6}$ | $4.499785 \times 10^{-8}$ | 0.54701 | 58.645175 | 2.105314 |
| 10,000 | $8.893653 \times 10^{-6}$ | $5.030301 \times 10^{-8}$ | 0.59893 | 58.192912 | 2.657495 |
| 20,000 | $8.881447 \times 10^{-6}$ | $1.153928 \times 10^{-7}$ | 0.69942 | 58.322803 | 1.407958 |
| 22,000 | $8.942604 \times 10^{-6}$ | $1.135189 \times 10^{-7}$ | 0.71438 | 58.353846 | 1.312924 |
| 25,000 | $8.940558 \times 10^{-6}$ | $6.403005 \times 10^{-8}$ | 0.76213 | 58.772360 | 0.716059 |

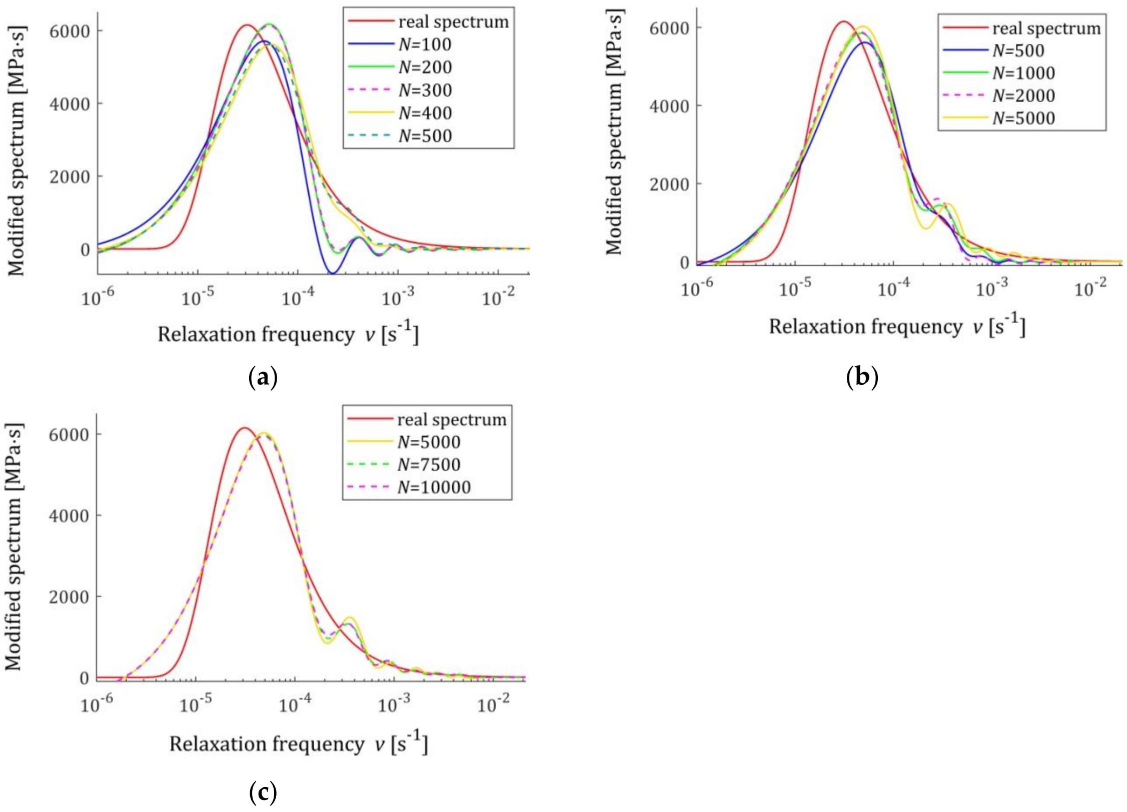


**Figure S5.** Optimal models $H_K^M(v, \bar{\boldsymbol{g}}_{N,K}^{\lambda})$ (10) defined by Laguerre basis functions $h_k(v)$ (A20) of the time-scale factor $\alpha = 8000$ s and $K = 14$ model components, determined with the regularization parameter $\lambda = 3.2 \times 10^{-11}$ s$^{-1}$, and the BSW relaxation spectrum $H^M(v)$ (63) (solid red line) for noises of standard deviation $\sigma = 0.001$ MPa and selected values of the number of measurements $N$: (**a**) $N \le 500$; (**b**) $500 \le N \le 5000$; and (**c**) $N \ge 5000$..

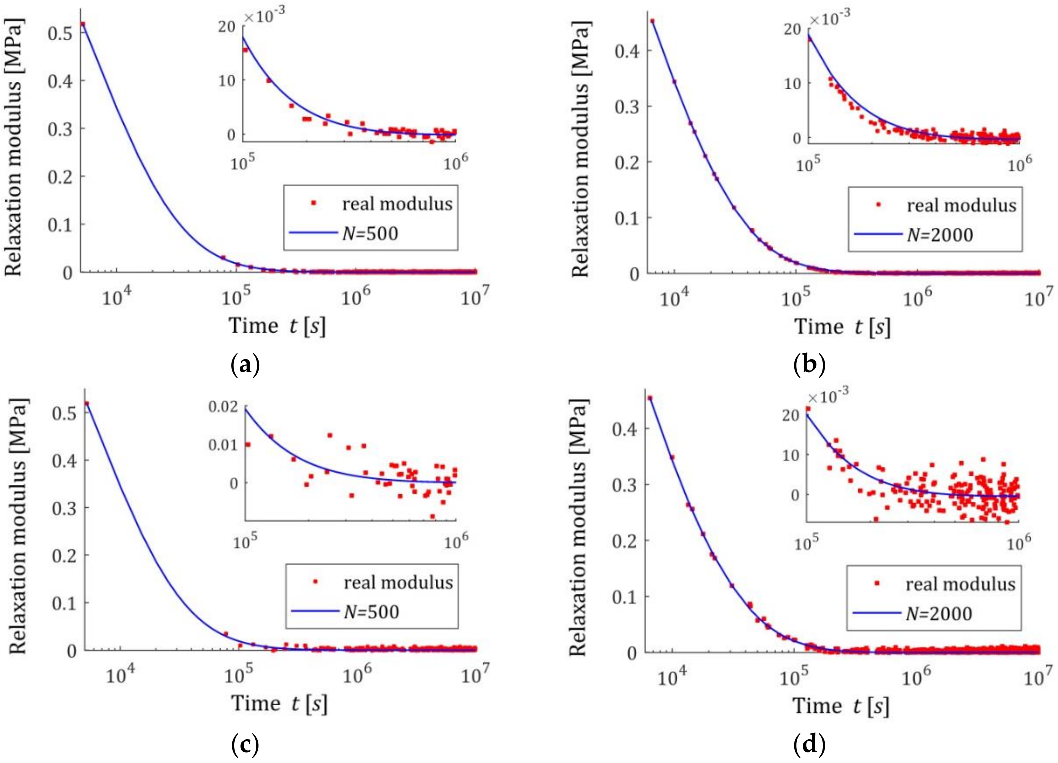


**Figure S6.** Optimal models $G_K(t, \bar{\boldsymbol{g}}_{N,K}^{\lambda})$ (15) with basis functions $\phi_k(t)$ (A22) corresponding to Laguerre functions $h_k(v)$ (A20) of the time-scale factor $\alpha = 8000$ s and $K = 14$ model components, determined with the regularization parameter $\lambda = 3.2 \times 10^{-11}$ s$^{-1}$, approximating the measurements $\bar{G}(t_i)$ (red points) of the BSW relaxation modulus (related to the relaxation spectrum $H^M(v)$ (63)) for selected values of the number of measurements $N$, indicated in the plots and noises of standard deviations: (**a**,**b**) $\sigma = 0.0005$ MPa; (**c**,**d**) $\sigma = 0.003$ MPa.
